# A self-triggering and fully sustainable gastric device enabling long-acting oral delivery of macromolecules

**DOI:** 10.1126/sciadv.aef9186

**Published:** 2026-08-28

**Authors:** Chengbang Lu, Zede Yi, Hongyan Yuan, Haoying Wang, Kewen Lei, Weijie Li, Xianglin Wu, Weixuan Liu, Yunxiang Zhang, Teng Dong, Kai Wu, Yaoben Wang, Shuquan Cui, Yue Zhao, Yingning He, U. Kei Cheang, Tian Liu, Yancong Zhang, Tianyuan Ci, Yiyuan Yang, Xiangyu Liang

**Affiliations:** ^1^ Agricultural Genomics Institute at Shenzhen, Chinese Academy of Agricultural Sciences, Shenzhen 518000, China.; ^2^ The N.1 Institute for Health, National University of Singapore, Singapore, Singapore.; ^3^Institute of Bast Fiber Crops and Center of Southern Economic Crops, Chinese Academy of Agricultural Sciences, Changsha 410205, China.; ^4^ Department of Biomedical Engineering, National University of Singapore, Singapore, Singapore.; ^5^ Shenzhen Hospital, University of Chinese Academy of Sciences, Shenzhen 518000, China.; ^6^ Department of Mechanical and Energy Engineering, Southern University of Science and Technology, Shenzhen 518055, China.; ^7^ Department of Biological Engineering, Massachusetts Institute of Technology, Cambridge, MA 02139, USA.; ^8^ Department of Materials Engineering, University of California, Los Angeles, CA 90095, USA.; ^9^ School of Physics and Optoelectronics, Xiangtan University, Xiangtan, Hunan 411105, China.; ^10^ Department of Chemical Engineering, University College London, London WC1E 7JE, UK.; ^11^ School of Bioengineering and School of Physics, Dalian University of Technology, Dalian 116024, China.; ^12^ State Key Laboratory of Molecular Engineering of Polymers, Department of Macromolecular Science, Fudan University, Shanghai 200438, China.; ^13^ Department of Chemistry, University of Minnesota, Minneapolis, MN 55455, USA.; ^14^ Department of Laboratory Medicine, Chongqing General Hospital School of Medicine Chongqing University, Chongqing 401147, China.; ^15^ School of Pharmacy, Shanghai University of Traditional Chinese Medicine, Shanghai 201203, China.; ^16^ Institute for Digital Medicine, Yong Loo Lin School of Medicine, National University of Singapore, Singapore, Singapore.; ^17^ National University of Singapore Guangzhou Research Translation and Innovation Institute, Guangzhou 510700, China.

## Abstract

Long-acting oral delivery of macromolecular therapeutics remains difficult, as most oral delivery devices focus on improving gastrointestinal absorption but do not adequately address sustained drug release and frequent dosing. In parallel, many gastric-retentive systems rely on nondegradable structural components, raising concerns regarding retained-device burden and postuse medical waste. Here, we report a biodegradable oral capsule device for long-lasting drug delivery, termed the sustainable gastric self-triggered device (SGSTD), inspired by the stinging mechanism of *Apis mellifera*. After reaching the stomach, SGSTD autonomously deploys a biodegradable barbed drug-loaded needle into the gastric wall, while the remaining carrier body is subsequently excreted, establishing a bioinspired “deploy-and-separate” strategy for long-acting gastric delivery. The embedded needle enables microchannel-mediated sustained release, with structurally tunable release behavior. Ex vivo and in vivo porcine studies confirmed gastric deployment, tissue retention, systemic safety, and localized tissue repair around the insertion region. In vivo, SGSTD maintained insulin delivery for over 7 days, achieving a relative bioavailability of 63.8%, approximately 17.2-fold higher than conventional oral administration. SGSTD is cost-effective, scalable, and composed of biodegradable materials, providing a sustainable strategy for long-acting oral biologics delivery.

## INTRODUCTION

Since the advent of macromolecular therapeutics, including peptides and proteins, injection and infusion remain the primary modes for administering macromolecular therapeutics (*1*), yet they are invasive and frequently associated with pain, fear, and poor patient adherence, particularly for chronic conditions such as diabetes and autoimmune diseases (*2*, *3*). In high-income countries, adherence to biologic treatments among patients with chronic diseases averages only ∼50% (*3*, *4*), and this figure is even lower in resource-limited settings. Oral administration, by contrast, is overwhelmingly preferred by patients due to its convenience, accessibility, and cost-effectiveness (*5*, *6*). However, oral administration of biologics faces formidable gastrointestinal (GI) barriers, including enzymatic degradation, harsh physicochemical conditions, and limited mucosal permeability (*7*, *8*). Despite decades of effort involving permeation enhancers and enteric coatings, macromolecular bioavailability remains below 1% (*9*, *10*).

Prolonging GI residence has emerged as a critical strategy for enhancing oral drug delivery by extending local retention, improving absorption opportunities, and supporting sustained therapeutic exposure (*11*–*13*). Recent engineering platforms (*14*, *15*), including self-orienting gastric systems, mucosal adhesive interfaces, and gastric-retentive robotic capsules, have further demonstrated the value of prolonged GI residence for local retention and responsive oral delivery. However, most existing systems still mainly focus on improving delivery efficiency after oral administration, whereas the pharmacodynamic duration of orally delivered macromolecules remains limited. As a result, frequent administration is still required in many chronic treatment scenarios, which does not fundamentally resolve the adherence burden associated with repeated dosing (*16*). Moreover, frequent use of gastric-retentive devices raises an additional but often overlooked sustainability concern: many systems rely on nondegradable plastic or metallic components, and frequent dosing may further increase postuse medical waste and retained-device burden. These limitations highlight the need for long-acting oral delivery platforms that can achieve sustained drug release to reduce dosing frequency, minimize whole-device retention to lower obstruction risk, and use fully biodegradable materials to alleviate environmental burden. To address these multifaceted challenges, we propose a structurally simple, sustainable, oral platform for long-acting macromolecular delivery.

**Fig. 1.**
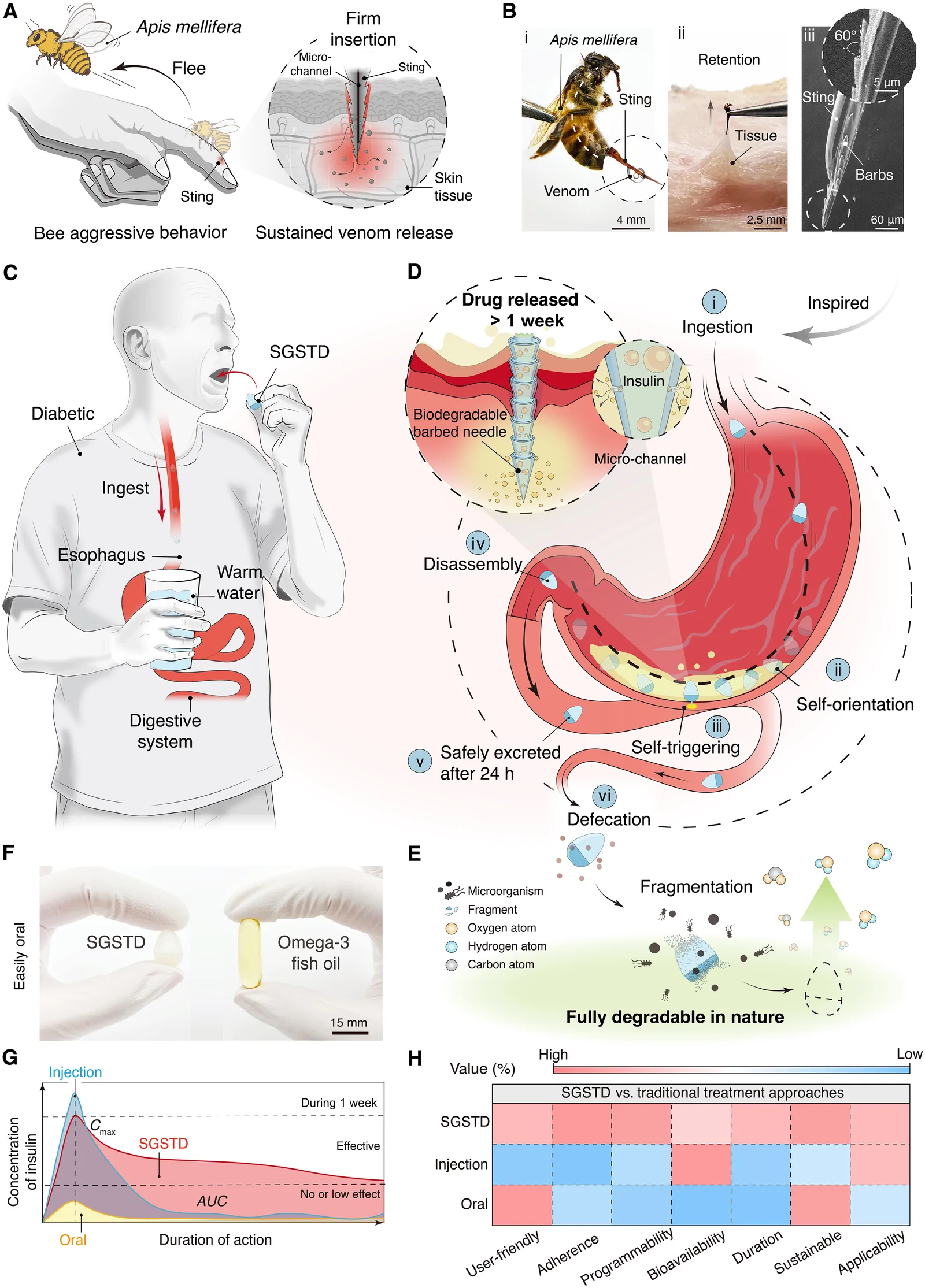
Design and execution route of SGSTD. (**A**) The aggressive behavior of *A. mellifera* during a threat. Although *A. mellifera* flees after stinging, its barbed sting remains firmly embedded into the skin tissue. Venom is transported through internal microchannels within the sting and sustained released along the barbed edges. (**B**) Structure and retention effect of the *A. mellifera*’s sting: (i) image of sting with venom release; (ii) sting retention effect during extraction; (iii) SEM of barb structure of sting. (**C**) Oral administration of SGSTD in a patient with diabetes. Inspired by the particular behavior of *A. mellifera* and its barb structure of sting, SGSTD is an oral drug delivery device designed to reach the stomach for sustained release. (**D** and **E**) Schematic of SGSTD execution route: (i) ingestion; (ii) self-orientation; (iii) self-triggering in the acidic environment; (iv) disassembly between the biodegradable barb-shaped needle and device, allowing prolonged insulin release through microchannels for more than 1 week; (v) safely excreted after 24 hours; (vi) defecation. The remaining device is excreted and eventually all biodegrades in natural soil. (**F**) Comparison of SGSTD with commercial omega-3 fish oil capsule. (**G** and **H**) Comparative analysis of SGSTD and traditional diabetic treatments (injection and oral administration), showing insulin concentration dynamics over 1 week (G) and benchmark-based evaluation across seven metrics (H). In (H), “high” indicates painless administration, lower dosing frequency, tunable release/dosing, higher relative bioavailability, longer duration, degradable materials, or applicability to macromolecular drugs; “low” indicates the opposite. Detailed criteria are summarized in table S1 and (*7*, *9*, *10*, *50*–*54*).

In searching for innovative solutions, the natural world offers valuable inspiration for engineering innovations (*17*–*20*). *Apis mellifera* uses a sacrificial defense mechanism during an attack (*21*). Before stinging, abdominal muscle contraction stores energy to drive the stinger into the skin like a bow and arrow (*22*), leaving the barbed stinger and venom sac embedded to sustain venom delivery after escape (*21*, *23*). The barbed structure ensures unidirectional penetration and resists extraction, while internal microchannels direct venom flow for prolonged release (Fig. 1, A and B) (*24*).

Drawing inspiration from the honeybee’s defense mechanism, we have developed an orally administrated, capsule-form drug delivery system, termed the sustainable gastric self-triggered device (SGSTD) (Fig. 1, C and D). Upon reaching the stomach, a pH-responsive trigger, mimicking the bow-and-arrow mechanism of *A. mellifera*, autonomously activates and ejects a biodegradable barbed drug-loaded needle into the gastric mucosa. Mirroring the bee’s sting separation mechanism, the needle separates from the device upon insertion while the carrier is excreted, eliminating the risk of GI obstruction. The embedded needle channels facilitate prolonged drug release through a microchannel diffusion mechanism, achieving blood glucose control effects comparable to subcutaneous administration. Manufactured using scalable technologies and US Food and Drug Administration (FDA)–approved, biodegradable, and biocompatible materials, the SGSTD ensures both efficacy and environmental sustainability (Fig. 1E). It is smaller than standard commercial omega-3 fish oil capsules (Fig. 1F) and the FDA-approved once-daily osmotic controlled-release oral delivery system (OROS, Ø 9 mm by 15 mm). OROS, a nondegradable drug delivery capsule, has a reported obstruction rate of only 1 in 50 million cases (*25*). In porcine models, the device enabled sustained oral delivery of insulin for over 7 days with superior relative bioavailability (63.8%, ∼17.2× increase), substantially outperforming both conventional oral and subcutaneous methods (Fig. 1G). By integrating patient-friendly, cost-effective, personalized and environmentally responsible features into a single orally administrated platform, the SGSTD represents a transformative advancement in oral biologics delivery, with the potential for broad therapeutic and global health impact (Fig. 1H).

## RESULTS

### Self-orientation mechanisms and safety assessment

**Fig. 2.**
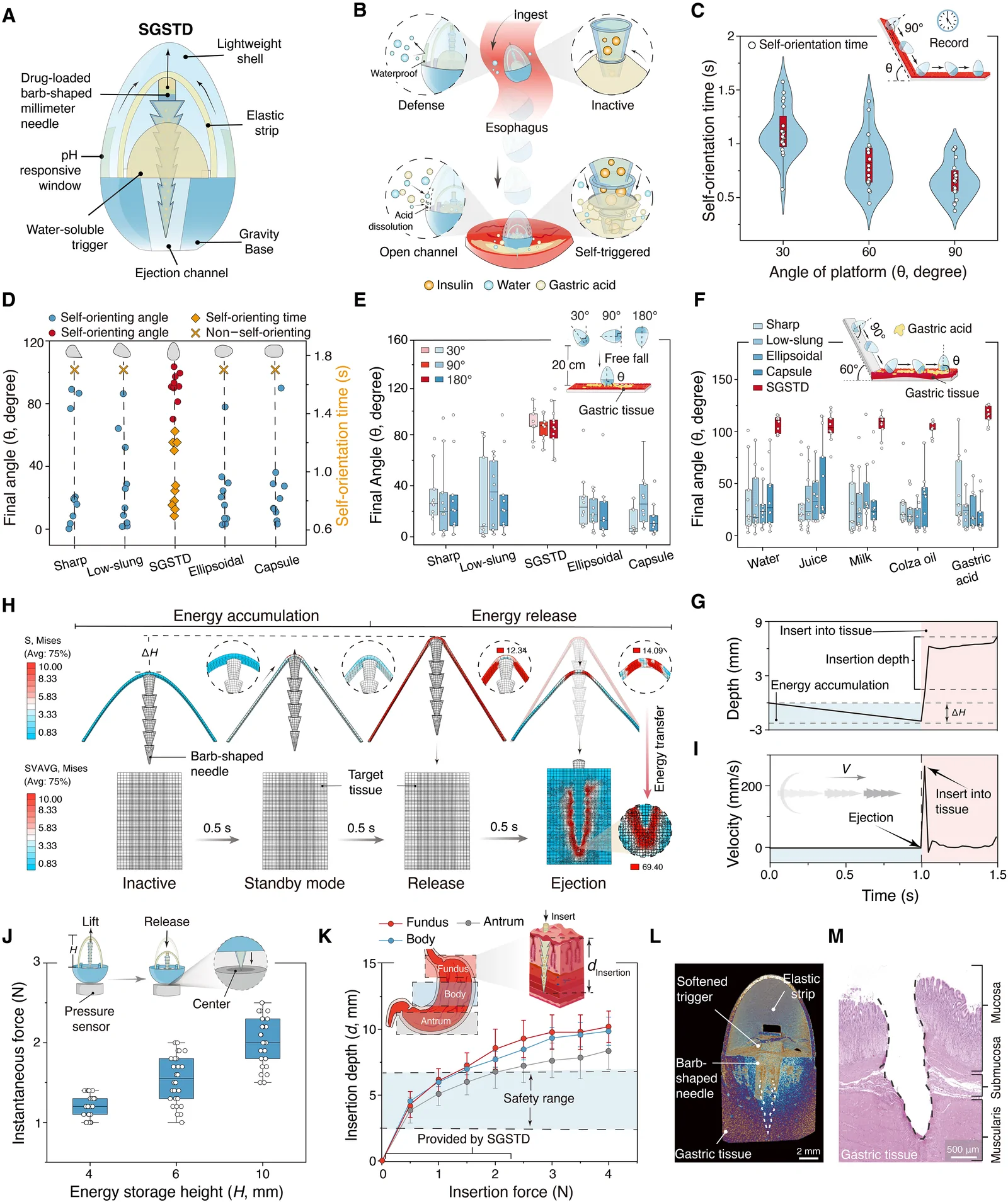
Self-orientation and safety evaluation of SGSTD. (**A** and **B**) The structure and main components of SGSTD, and schematic diagram of the SGSTD’s anti–false-touch mechanism and pH-responsive ejection system. (**C**) Self-orientation time required of SGSTD freely rolling on ex vivo gastric tissue from different initiating angles. (**D**) Mechanical interference. (**E**) Self-orientation angles of devices with different shapes freely dropping onto the tissue from different initiating angles. (**F**) Self-orientation angle of devices under the different liquid environment in the stomach. (**G** to **I**) Mechanical simulation of the ejection mechanism for barb-shaped needle. (G) The process of energy accumulation, release, and transfer of barb-shaped needle, accompanied by the actions (inactive, standby, release, and ejection) in order. (H) The depth-time curve of the barb-shaped needle during energy storage and release, showing insertion depth and tissue contact. (I) The instantaneous velocity-time curve of the barb-shaped needle during ejection, highlighting the peak speed upon tissue insertion. (**J**) The correlation between the height and the instantaneous elastic force of the barb-shaped needle during ejection. Pressure sensor for measuring the instantaneous elastic force of the barb-shaped needle (inset diagram). (**K**) Assessing the variation in penetration depth of barb-shaped needles in different locations of ex vivo stomach tissue under varying insertion forces. The diagram illustrates the safety testing procedure for measuring the depth of insertion. (**L** and **M**) Micro-CT images of SGSTD after self-triggering on ex vivo stomach tissue and histological analysis of the insertion depth.

The SGSTD comprises five main components: shell, protective film, pH-responsive ejection system, barb-shaped needle, and base (Fig. 2A). According to the FDA’s MAUDE database, more than 2 million injury reports annually result from medical device misuse or design flaws (*26*). To address this, the SGSTD incorporates a chitosan-based protective barrier that prevents premature needle release potentially triggered by esophageal fluids (Fig. 2B). Here, “self-triggered” refers to an autonomous activation process in which the device remains mechanically locked during esophageal transit but is activated by the acidic gastric environment without external manipulation. Mechanistically, the chitosan-based pH-responsive film remains stable in the near-neutral esophageal environment, thereby shielding the internal gelatin-based trigger and maintaining the constrained state of the ejection system (fig. S1A). After the device enters the acidic stomach, the protective film gradually dissolves and exposes the water-soluble gelatin trigger, which subsequently swells, hydrates, and disintegrates. This acid-triggered loss of constraint releases the elastic restoring force stored in the preloaded ejection system and converts it into a downward insertion force, driving deployment of the barb-shaped needle into the gastric wall (fig. S1B). The SGSTD is fabricated using fully biodegradable materials through three-dimensional (3D) printing (movie S1), making it both eco-friendly and customizable.

The highly dynamic gastric environment, characterized by continuous peristaltic movements, irregular folding of gastric tissues, and varied fluid flow, necessitates stable device orientation to consistently position the drug-loaded needle toward the gastric wall for precise insertion (*27*, *28*). To address this requirement, the SGSTD was engineered with a gravity-driven self-orienting design that leverages the physical principle of roly-poly toy, where a low-offset center of gravity beneath a curved upper shell generates a restorative torque that promotes upright realignment after perturbation. Specifically, positioning the center of mass notably below the geometric midpoint allows the device to inherently counteract overturning forces, facilitating automatic reorientation after disturbances. A systematic optimization process involving five geometric variants (sharp, low-slung, SGSTD, ellipsoidal, and capsuled) identified the SGSTD as optimal, featuring the smallest upper curvature radius (*R* = 0.1), which maximizes restorative torque during angular displacement (fig. S2 and tables S2 to S6). Computational analyses further showed that SGSTD had both the lowest vertical center of mass and the largest principal moments of inertia (*I_xx_* = 8.34, *I_yy_* = 8.34, *I_zz_* = 9.28) among all tested designs (fig. S3), providing stronger resistance to angular acceleration and enhancing stability after oral administration. In comparison to flat tablets, which are prone to esophageal adherence and potential injury, the quasi-elliptical design of the SGSTD enhances ease of swallowing, improving patient comfort and safety (*29*).

To assess the self-orientation capability of the SGSTD, free-fall tests (*n* = 100) were initially conducted on a flat plane and within an in vitro stomach model (movies S2 and S3), resulting in successful self-orientation (≥90°) in 495 out of 500 trials (99%). When rolled onto ex vivo gastric tissue from an adjustable inclined platform, the SGSTD reliably self-oriented within 0.69 to 1.35 s (Fig. 2C and movie S4), further underscoring its capability for rapid stabilization following gastric entry. To further evaluate device stability under conditions mimicking gastric peristalsis, ex vivo porcine gastric tissue was placed on a shaker platform and subjected to continuous rotational shaking at 70 rpm for 20 s (Fig. 2D, fig. S4, and movie S5). Under this dynamic condition, the SGSTD consistently achieved self-orientation to an angle of ≥90° within 0.98 ± 0.21 s, outperforming other tested geometries. This rapid orientation is critical to minimize delays between device deployment and stable needle alignment, reducing the risk of misalignment and facilitating accurate, timely drug delivery. Physical simulations were conducted to mimic the SGSTD’s free-fall motion as if released from the pyloric region, demonstrating consistent self-orientation angles of ≥90° upon landing from various initial positions (Fig. 2E and movie S6). Last, tests in simulated gastric fluid environments demonstrated that the SGSTD retained robust orientation performance, even on liquid-covered mucosal surfaces (Fig. 2F, fig. S5, and movie S7). Collectively, these findings confirm that the SGSTD’s optimized low center of gravity and large moment of inertia ensure rapid and stable orientation, consistently positioning the device perpendicular to the gastric mucosa for spatiotemporally precise and sustained therapeutic administration.

The acid-triggered activation behavior of SGSTD was first supported by degradation tests of the pH-responsive window and water-soluble interceptor in artificial gastric fluid, which confirmed that both components lost structural integrity under acidic conditions (fig. S6). These results provide the material basis for the self-triggered activation of SGSTD. Considering the complex gastric environment in vivo, including heterogeneous fluid access, tissue contact, and dynamic gastric motion, we further evaluated whether this material-driven activation process could be translated into reliable needle deployment in the stomach. Fixed–time point endoscopic assessment was therefore performed using an interval-bracketing strategy (fig. S7A). SGSTD remained unactivated at 10 s, whereas activation began to appear at 40 s. Complete activation was observed at both 55 and 70 s, while two of three devices were activated at 100 s, with one failure likely caused by interference from solid gastric contents. Overall, the activation success rate was 75.0% within the tested 40- to 100-s interval and increased to 88.9% within the 55- to 100-s interval. These results indicate that SGSTD activation begins between 40 and 55 s and occurs mainly within 55 to 100 s after administration (fig. S7B). The observed variability may arise from differences in gastric fluid entry and device wetting, which are influenced by interfacial liquid tension and local heterogeneity of the gastric environment. Together, these results show that acid-triggered material disintegration enables timely in vivo needle deployment for localized gastric insertion and subsequent sustained release, while reducing the risk of premature activation.

Drug delivery devices that administer therapeutics via submucosal injection have been demonstrated as a safe and effective approach for localized delivery in the GI tract (*30*). Clinical evidence, including the use of 5-mm 25-gauge Carr-Locke needles, further supports the safety of this approach (*31*). With a thickness of 4 to 8 mm, the stomach wall is a safer and more suitable target for physical drug delivery compared to the thinner intestinal wall (0.5 to 2 mm) (*32*). To evaluate the safety and efficacy of the SGSTD’s barb-shaped needle, mechanical simulations of the pH-responsive ejection system were conducted (Fig. 2, H and I; fig. S8; and movie S8), alongside experimental measurements of insertion force and penetration depth (Fig. 2J). The SGSTD’s needle ejects with a controlled force of 1.2 ± 0.14 N to 2.0 ± 0.32 N, achieving insertion depths of 4.45 ± 0.24 mm in the fundus, 5.36 ± 0.37 mm in the body, and 5.55 ± 0.77 mm in the antrum, all within the safe range (Fig. 2K) (*32*). Micro–computed tomography (CT) imaging of SGSTD activation on ex vivo gastric tissue in simulated acidic gastric fluid (Fig. 2L and fig. S9) confirmed reliable, perpendicular needle insertion without perforation. Repeated trials and histological analysis (Fig. 2M) further validated safe insertion depths of 2.5 to 6 mm in porcine gastric tissue, with no evidence of perforation.

### Design, fabrication, and evaluation of barb-shaped needle

**Fig. 3.**
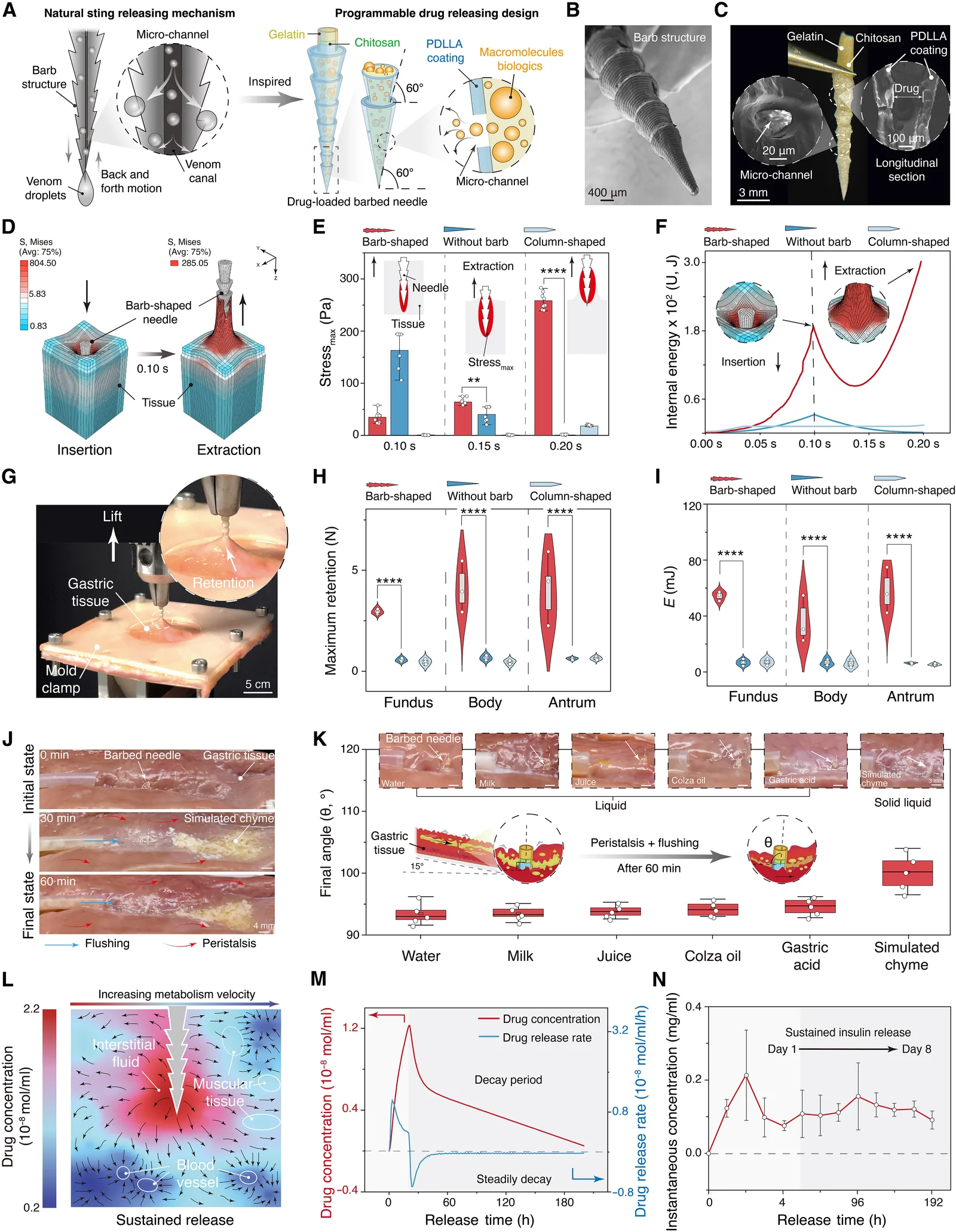
Fabrication and functional characterization of drug-loaded barb-shaped needles. (**A**) Inspired by barbed bee stingers, barb-shaped needle was designed with microchannels and degradable coatings to achieve controlled release of macromolecular biologics. (**B** and **C**) SEM images showing the barb structure, microchannels, and sectional morphology. (**D**) Mechanical simulation of retention tissue and stress distribution. (**E**) The maximum stresses during the various needle shapes being lifted in the tissue model, showing the stress changes in the tissue during the extraction (inset figure). (**F**) Internal energy change curves of tissues during the insertion and extraction of needles with various shapes. Cloud diagrams of the stress distribution from the finite element simulation were shown as insets. (**G**) Retention effect of barb-shaped needles in porcine gastric tissue. (**H**) The maximum retention (*F*_max_) generated by the barb-shaped needle extraction from various porcine stomach positions. (**I**) Different fluids and flow rates were used to assess barb-shaped needle movement while it was inserted vertically into ex vivo tissue. (**J** and **K**) Long-duration cyclic mechanical loading test under solid-liquid mixed gastric conditions. Representative images showing the barb-shaped needle retained in gastric tissue during 60 min of cyclic peristalsis flushing with simulated chyme. Blue arrows indicate fluid flushing, and red arrows indicate peristaltic-like deformation (J). The final needle angle after 60 min of cyclic mechanical loading under different liquid and solid-liquid media, including water, milk, juice, colza oil, gastric acid, and simulated chyme (K) (*n* = 5). (**L** and **M**) Drug controlling release simulation of drug-loaded needle in stomach tissues. Drug concentration distribution of barb-shaped needle with microchannels while it was inserted into stomach tissue (L). The arrows indicate the direction of drug diffusion. Drug concentration and release rate change over time curves (M). (**N**) In vitro insulin controlling release curves of barb-shaped needle for 8 days (*n* = 5).

To achieve long-term gastric tissue retention and sustained drug release, we drew inspiration from the stinger of *A. mellifera*, which firmly anchors into tissue via backward-facing barbs and delivers venom through internal microchannels during insertion. Mimicking this natural structure and function mechanism, we designed a drug-loaded needle with a fully barbed architecture for mechanical anchoring and two lateral microchannels to enable controlled diffusion of macromolecular biologics (Fig. 3A). The needle was fabricated using a template molding method (fig. S10) and consists of a gelatin-based drug matrix and an outer coating of poly-d,l-lactic acid (PDLLA). The gelatin, blended with the active pharmaceutical ingredient (API), forms a rapidly dissolving core. In contrast, PDLLA, a widely used FDA-approved biocompatible polymer for controlled drug delivery, serves as a slow-degrading coating. The key to sustained release lies in the degradation rate difference of the above materials: while gelatin dissolves relatively quickly in the gastric environment, the denser PDLLA shell acts as a physical barrier that limits the exposure of the inner drug matrix. To allow for controlled release, two lateral microchannels were incorporated into the PDLLA coating, enabling the gelatin-API mixture to diffuse outward in a gradual and sustained manner (Fig. 3A and fig. S10) (*33*). Scanning electron microscope (SEM) imaging revealed the distinct barbed structure, PDLLA coating, and microchannels (Fig. 3, B and C, and fig. S11). Micromechanical properties were evaluated and compared before and after insulin incorporation. Nanoindentation at a 1-μm depth revealed that the reduced modulus of gelatin-insulin formulations (220 ± 20 MPa) was comparable to that of pure gelatin (180 ± 10 MPa), indicating that drug loading did not affect the matrix stiffness (fig. S12). In addition, force-displacement curves showed similar trajectories and peak forces (∼5000 μN), further confirming the consistency of the material’s mechanical response (fig. S12).

Finite element analysis (FEA) elucidated the role of the geometrical designs of the needle with respect to the retention period. Barbed structures generated a distinctive volcano-shaped stress contour during extraction and exhibited substantially higher internal energy accumulation than cylindrical and nonbarbed designs (Fig. 3, D to F; fig. S13; and movie S9), resulting in drastically elevated tissue anchoring that required ∼30 times more energy for detachment compared to nonbarbed structures. In vitro insertion and extraction studies on retention efficacy across different gastric regions (fundus, body, and antrum) (Fig. 3G, fig. S14, and movie S10) further confirmed our FEA analysis. Barb-shaped needles demonstrated obvious retention enhancement compared to cylindrical and nonbarbed needles (Fig. 3, H and I, and fig. S14), with a maximum extraction force of 3.9 ± 0.37 N and extraction energy of 60.67 ± 11.73 mJ, approximately 6.74 times and 7.83 times higher than cylindrical (9.0 ± 0.82 mJ) and nonbarbed needles (8.33 ± 1.25 mJ), respectively.

Documented studies have reported that the stomach generates peristaltic “mixing waves” producing mechanical forces of ∼2 N, posing a substantial challenge to drug-loaded device retention. The barbed configuration provides a retention force (3.9 ± 0.37 N) far exceeding this physiological threshold, ensuring robust mechanical interlocking with tissue (*34*). Given the dynamic and fluid-rich gastric environment during daily intake, we evaluated the retention stability of the barb-shaped needles under both short-term liquid flushing and long-duration cyclic mechanical loading. In the 20-s flushing tests with different liquid types and flow rates, the needles showed only limited angular deviation (0.64° ± 1.87° to 10.73° ± 5.63°), with no observed detachment (fig. S15 and movie S11). To further approximate gastric peristalsis and chyme-associated disturbance, we performed a 60-min cyclic mechanical loading test using peristalsis-assisted flushing with solid-liquid simulated chyme (Fig. 3, J and K, and movie S12). Under sustained peristaltic loading and flushing, the barb-shaped needles remained embedded in the ex vivo gastric tissue. Even in the solid-liquid simulated chyme group, the final needle angle remained close to the initial reference angle, with only a limited angular deviation of approximately 10.14° ± 3.01° from the initial 90° reference angle after 60 min. These results support the robust antidetachment stability of the barb-shaped structure under prolonged gastric-like mechanical disturbance.

Controlled drug release at target rate is mandatory for optimized therapeutic efficacy. Our needle can achieve such controlled drug release via tunable microchannel numbers and environmental temperature. Adjusting the number of microchannels provides a simple structural approach to modulate the in vitro release rate of the loaded drug, which may help guide the future design of release rate, release duration, and dosing amount for different therapeutic requirements (fig. S16). The drug release rate can be further controlled via external temperature. Our in vitro diffusion studies, conducted across a range of externally achievable and physiologically safe temperatures (15° to 65°C) (fig. S17) guided by commonly accepted warm beverage intake temperatures (∼65°C) (*35*, *36*), demonstrated that elevated temperatures accelerated drug diffusion. Thermal cycling (e.g., 37° to 55°C) produced modulated release profiles, enabling temperature-responsive control over drug release rates (fig. S17). This tunability offers a promising strategy for aligning drug release with the temporal and dosage requirements for personalized disease treatments. However, considering that gastric temperature is homeostatically regulated (*37*) and that ingestion of very hot liquids may raise mucosal safety concerns, we do not propose hot-liquid ingestion as a routine method for regulating drug release. Instead, these results provide an experimental basis for future externally controlled release strategies, such as localized endoscopic thermal input or other controlled heating approaches, which will require further safety and feasibility evaluation.

Furthermore, FEA was used to simulate the release of insulin through microchannels in barb-shaped needles (Fig. 3, L and M, and movie S13) to explore the drug diffusion in tissue. The simulation focused on local drug concentration changes within the tissue, revealing the temporal drug release pattern during initial burst and stabilized phases. In vitro simulated release studies using insulin-loaded barb-shaped needles were performed to evaluate the sustained-release behavior of insulin as the intended macromolecular drug. High performance liquid chromatography quantification showed approximately 20% insulin release within the first 5 hours and sustained release over 8 days (Fig. 3N and fig. S18). The discrepancies on the trend of dosage between the experiment and FEA simulation can be explained by the simplification of dynamic fluid flow, enzymatic degradation, and heterogeneous tissue permeability. These results collectively demonstrate the programmable release kinetics via the synergistic interplay of microchannel geometry, thermal responsiveness, and material biodegradation within the needle. This tunable system provides a robust framework for long-acting drug delivery and lays the groundwork for customizable, controlled-release therapeutics. Such modularity is applicable across a broad class of biologics and disease indications, highlighting the system’s versatility and translational potential.

### Long-acting drug delivery in vivo and safety assessment

**Fig. 4.**
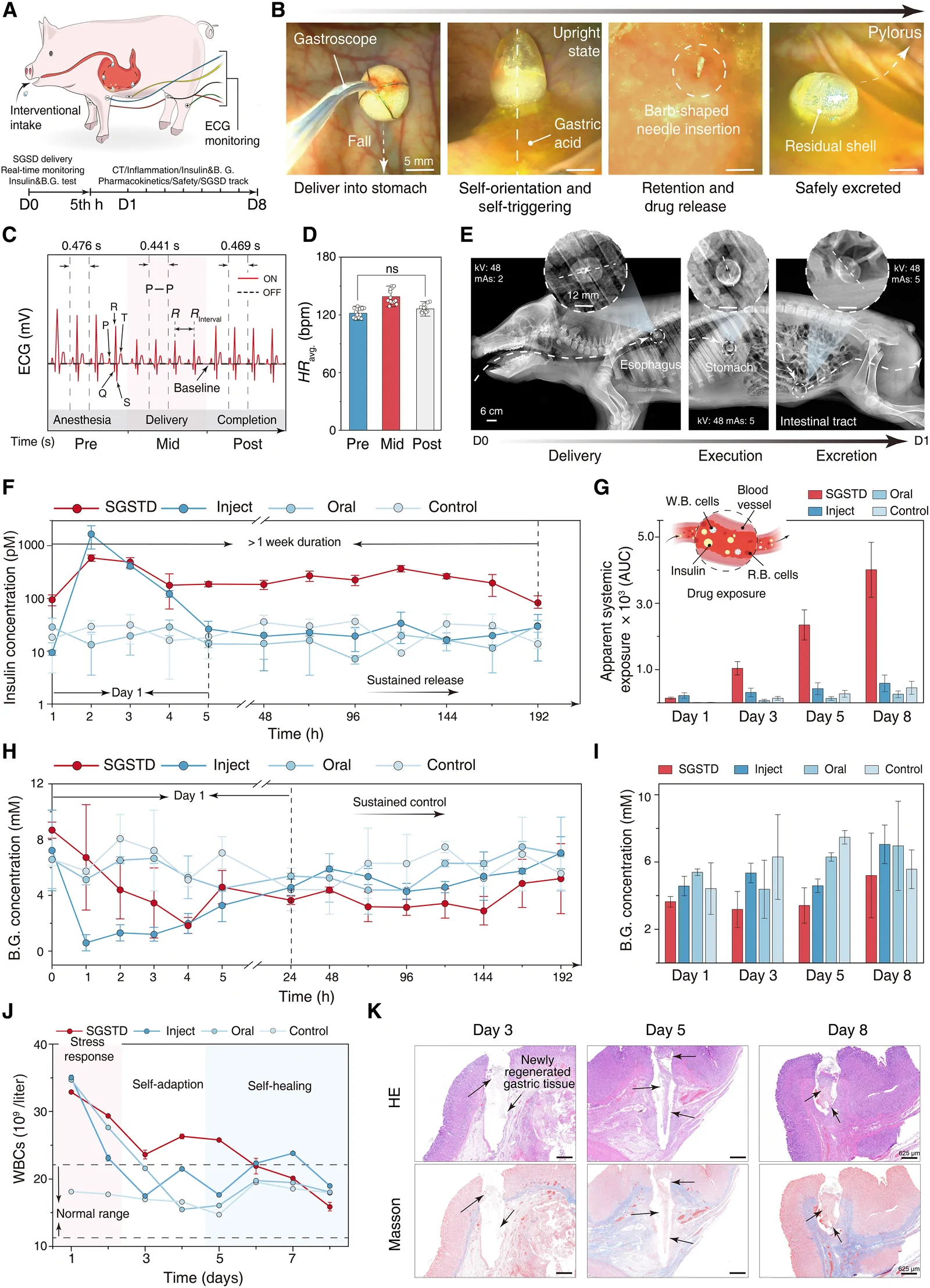
Pharmacodynamic assessment in large mammals. (**A**) Schematic representation of in vivo experiments using the pig model. (**B**) Endoscopic images showing the complete process of SGSTD administration in the pig stomach. (**C** and **D**) Continuous heart rate monitoring during SGSTD administration, illustrating heart rate stability. (**E**) X-ray tracing of SGSTD movement through the gastrointestinal tract, capturing gastric retention, drug release, and excretion phases. (**F** and **G**) Blood insulin levels and drug exposure (AUC) during in vivo experiments, showing sustained high insulin concentrations over 1 week of SGSTD administration. The inset highlights drug exposure effects in blood vessels. (**H** and **I**) Continuous blood glucose monitoring across four dosing regimens, with average blood glucose levels analyzed on days 1, 3, 5, and 8. (**J**) Inflammation assessment via white blood cell (WBC) counts at different stages (stress response, self-adaptation, and self-healing) over 1 week (*n* = 3). (**K**) HE and Masson staining of gastric tissues from the insertion region collected on days 3, 5, and 8. The arrow indicates the newly regenerated gastric tissue.

Cytocompatibility assessments of individual SGSTD components were conducted using cultured human gastric mucosal epithelial cells (*GES-1*). Fluorescent staining and cell viability assays indicated overall acceptable cytocompatibility of these components, with the day 8 viability results comparable to those of the control group (fig. S19). Bama pig models were selected for in vivo evaluation due to their anatomical resemblance to human stomach. As pigs typically chew when self-administering, SGSTD was delivered to the esophagus via an endoscope equipped with a foreign body clip (Fig. 4A). Endoscopic imaging confirmed successful deployment and stable positioning of SGSTD in the stomach (Fig. 4B). Stable heart rate throughout the procedure indicated good tolerability without signs of discomfort (Fig. 4, C and D). Subsequent x-ray imaging verified smooth excretion of SGSTD through the GI tract (Fig. 4E). By the following day, SGSTD was undetectable, indicating a gastric retention time of less than 24 hours.

SGSTD’s efficacy in delivering peptide drugs was further assessed in vivo using a human recombinant insulin model. Before evaluating the pharmacokinetic and glucose-lowering outcomes, we first verified whether the separated drug-loaded needle could remain at the gastric wall for the intended release period. Periodic endoscopic observation showed that the barb-shaped needle remained visible in the gastric wall on days 3, 5, and 8 (fig. S20), confirming local retention for more than 1 week. This result indicates that, although the SGSTD body was excreted within 24 hours, the insulin-loaded (0.5 mg) barb-shaped needle remained locally anchored in the gastric wall and functioned as a tissue-embedded depot for sustained release. Consistent with this structural retention, pigs receiving SGSTD maintained low average blood glucose levels (3.87 ± 0.93 mM) and high yet stable insulin levels (229.58 ± 66.26 pM) from 24 to 192 hours after administration (Fig. 4, F to J). On day 1, SGSTD achieved a relative bioavailability of 63.8%, approximately 17.2-fold higher than oral administration (3.7%). Over the 0- to 192-hour observation window, SGSTD maintained prolonged apparent systemic insulin exposure, characterized by sustained measurable insulin concentrations and continuous glucose-lowering effects throughout the 1-week study period (Fig. 4, F and G). SGSTD also showed slower plasma drug decline, as reflected by the reduced apparent terminal rate constant and extended mean residence time compared with subcutaneous injection (Fig. 4J and fig. S21), supporting its prolonged drug action.

Throughout the 1-week study, the pigs’ daily activities were closely monitored, including blood routine indicators (Fig. 4J and fig. S22) and behavioral pattern (table S7). Hematological indicators such as white blood cell, monocyte, and lymphocyte counts showed transient fluctuations on days 1 to 3, which are commonly attributed to physiological stress responses. All values returned to within normal reference ranges by day 7. In addition to systemic safety evaluation, we further examined the local tissue response at the needle insertion site. Histopathological evaluations of gastric tissues collected on days 3, 5, and 8 showed that the insertion remained localized within the gastric wall without evidence of perforation, severe ulceration, or progressive tissue destruction (Fig. 4K). The surrounding tissue displayed a localized reparative response, including newly regenerated gastric tissue and gradual structural recovery around the insertion tract, supporting acceptable local tissue safety during the 1-week retention period. No signs of inflammation, abnormal feces, or feeding irregularities were observed throughout the observation period. Notably, the SGSTD shell was observed in the pigs’ excretion within 24 hours of ingestion (fig. S23). Gastric peristalsis (∼3 contractions per minute) and intestinal radial contractions (∼10 times per minute; compressive force, 0.18 to 0.5 N/cm) (*38*) facilitated the rapid separation and safe excretion of the SGSTD shell. These implanted needles are composed of biodegradable gelatin and PDLLA. Gelatin can gradually swell, dissolve, and undergo enzymatic degradation in vivo, whereas PDLLA is a biodegradable polymer that degrades through hydrolysis in the body (*39*, *40*). Therefore, based on the material composition and the results of local retention and histopathological analysis, we reasonably anticipate that the implanted needle will gradually degrade within the body, and the likelihood of it leaving permanent residues is low. Following 1 week of blood monitoring, histological analysis of major organs posteuthanasia (fig. S24) confirmed the absence of toxic effects, demonstrating the safety of SGSTD for oral delivery.

### SGSTD’s biodegradability and sustainability

**Fig. 5.**
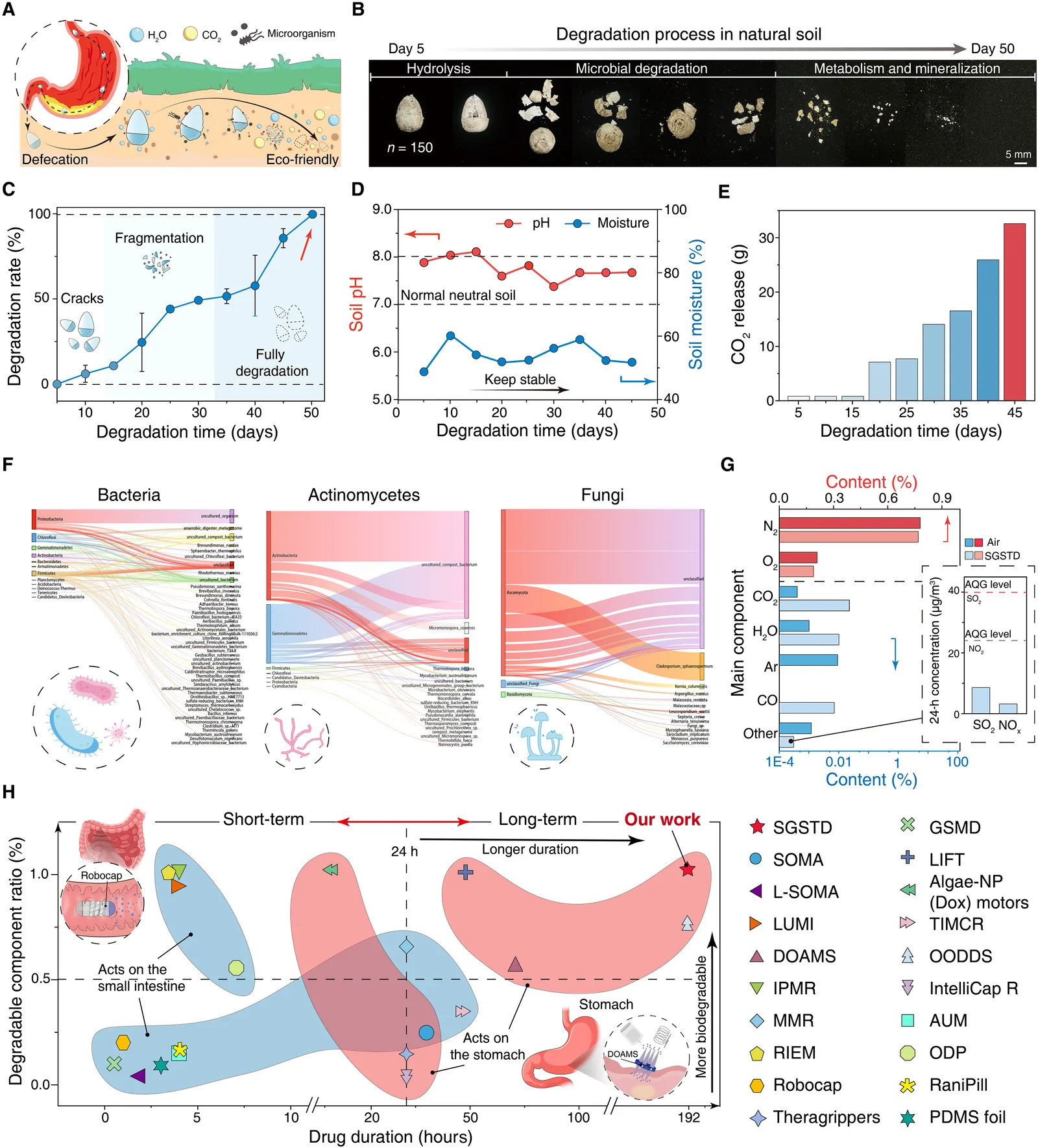
SGSTD’s biodegradability and environmental impact assessment. (**A**) Schematic representation of the complete biodegradation process of SGSTD. (**B**) Soil biodegradability tests showing full degradation of SGSTD within 50 days. Fragmentation begins on day 30, and no residual fragments remain by day 50. (**C**) Degradation rate curve illustrating the mass transition of SGSTD from fragmentation on day 30 to complete disappearance by day 50. (**D**) Soil pH and moisture conditions during the degradation process. (**E**) CO_2_ emissions from 150 SGSTD units during degradation, with each unit releasing ∼0.21 g of CO_2_ over 45 days. (**F**) Soil microbiome analysis post-SGSTD degradation using high-throughput sequencing, focusing on bacteria, radiolarians, and fungi. A Sankey diagram illustrates the distribution of species across phylum domains. (**G**) Medical waste incineration testing of SGSTD, with gas composition analysis comparing incineration emissions to ambient air. Zoomed-in graphs show SO_2_ and NO_x_ levels, both substantially below WHO air quality standards. (**H**) Comparison of the degradable component ratios and drug delivery strategies across systems. Devices are categorized by therapeutic approach: fast-acting systems (short-term release) and sustained systems (long-term release). Blue regions represent small intestine-targeted devices, while red regions represent stomach-targeted devices. Insets illustrate drug delivery to these regions. References: (*5*, *11*–*13*, *27*, *28*, *30*, *55*–*67*)*.*

Although the US FDA permits the disposal of nonbiodegradable solid dosage forms (e.g., OROS systems) via wastewater treatment (*9*, *25*), these materials may ultimately accumulate in the environment as pollutants. Building on prior validation of safety and efficacy, we assessed the environmental degradability of SGSTD, from material-level composition and biodegradation behavior to ecological and environmental impact. On the basis of its material composition, infrared spectroscopy was used to identify characteristic functional groups of PDLLA, chitosan, and gelatin, all of which are widely recognized as biodegradable polymers. PDLLA shell exhibited characteristic C─H and C═O peaks at 3000 to 2800 and 1750 cm^−1^ (fig. S25A); chitosan showed peaks for N─H/O─H and C─H at 3359 and 2867 cm^−1^ (fig. S25B); and gelatin displayed O─H and C─H peaks at 3300 and 2873 cm^−1^ (fig. S25C). Compost degradation tests using 300 SGSTD units (Fig. 5A) demonstrated complete biodegradation within 50 days (Fig. 5B). Fragmentation began on day 30, reaching 50% degradation by day 35, and the material completely disappeared into the soil by day 50 (Fig. 5C). Throughout the degradation period, soil pH (7.76 ± 0.23) and moisture content (55.02 ± 3.40) remained stable (Fig. 5D), and the main by-products were CO_2_ and H_2_O, indicating no adverse environmental impacts (Fig. 5E). Microbial viability in the soil, including bacteria, actinomycetes, and fungi, was evaluated via high-throughput sequencing. The microbial community structure and activity remained unchanged after SGSTD degradation, with stable overall abundance (Fig. 5F). These findings confirm that SGSTD decomposition is environmentally benign, leaving soil ecology undisturbed.

Recognizing that incineration accounts for 12% of conventional medical waste disposal methods (*40*), the environmental impact of SGSTD was further evaluated through incineration experiments and gas composition analysis (Fig. 5G). The primary constituents of the emitted gases, N_2_ (77.1%) and O_2_ (18.90%), closely matched atmospheric levels (N_2_: 78.08%, O_2_: 20.94%) (*41*). Sulfur and nitrogen compound emissions were substantially below WHO’s Air Quality Guidelines limits (*42*). Gas toxicity testing in mice models (movie S14) and subsequent histological analysis (fig. S26) confirmed the minimal environmental impact of SGSTD incineration. These results further reinforce the sustainability of SGSTD as an environmentally friendly medical device.

Last, given that SGSTD is constructed from biodegradable materials, we explored its potential environmental implications in long-acting macromolecular therapy under an idealized application scenario. On the basis of reported health care–emission data, diabetes prevalence projections, and simplified life-cycle assumptions (*43*–*47*), we estimated the possible effects of replacing injection method medical waste with the SGSTD platform on global warming potential and acidification potential (fig. S27). This analysis suggests that biodegradable long-acting delivery devices help reduce waste-related environmental burdens in high-frequency chronic treatment settings. Notably, given the complexity of real-world clinical implementation, these results should be interpreted as preliminary scenario-based estimates supporting the sustainability potential of SGSTD, rather than as direct predictions of clinical replacement.

We summarized the efficacy and degradable component ratios of various oral drug delivery devices (Fig. 5H and table S8). SGSTD demonstrated superior performance in prolonged drug delivery within the stomach, achieving sustained drug release for more than 1 week (192 hours) with a 100% degradable component ratio. These results highlight SGSTD’s substantial advantages in delivery duration and environmental sustainability, enabling long-term drug release while minimizing environmental contamination and safety risks from residual materials. Unlike devices that rely on external stimuli (e.g., magnetic fields) or internal batteries and electronic circuitry, SGSTD operates autonomously, eliminating the safety hazards and environmental impact associated with complex electronic components. This simplified design enhances safety and sustainability, offering a practical and eco-friendly solution for clinical applications.

## DISCUSSION

This study introduces SGSTD, a fully sustainable, self-triggering gastric device that enables long-acting oral delivery of macromolecular therapeutics. Inspired by the sting mechanism of *A. mellifera*, SGSTD autonomously deploys a barbed, drug-loaded needle into the gastric mucosa via a pH-triggered propulsion system. Its mechanical stability achieved through a low center of gravity and high rotational inertia ensures accurate tissue engagement, while safety is guaranteed by a pH-responsive window and water-soluble trigger that prevent premature activation. The embedded needle, engineered with customizable microchannels, differential degradation rates, and temperature-sensitive materials, enables programmable and sustained drug release tailored to therapeutic needs. In porcine models, SGSTD exhibited excellent biocompatibility, extended insulin delivery over 7 days with markedly enhanced oral bioavailability, and demonstrated complete degradation of all components, eliminating residual medical waste. Critically, SGSTD unites programmable pharmacokinetics, patient convenience, and environmental responsibility in a scalable platform. Its modular, biodegradable design extends beyond insulin to deliver a broad range of biologics including peptides, proteins, and nucleic acids. By offering an effective, eco-conscious, and clinically actionable alternative to injections, SGSTD represents a transformative platform with substantial potential to reshape global biologics delivery and sustainable health care.

SGSTD offers distinct advantages over conventional in vivo drug delivery systems, combining high delivery efficiency, extended release duration, and environmental sustainability. A key strength lies in its cost-effective and scalable manufacturing process, bringing it closer to commercial viability. Drug-loaded needles are fabricated via a batch casting and drying method, which preserves the structural integrity of fragile biomolecules and minimizes drug deterioration. The total production cost including materials, processing, and transportation was estimated at $2.31 per unit, making SGSTD particularly well suited for deployment in low-resource settings, where 75% of the global diabetic population resides, according to the International Diabetes Federation (*47*). Beyond low-cost fabrication, SGSTD may further alleviate long-term treatment burden and improve therapeutic accessibility by reducing dosing frequency. Furthermore, the device is composed entirely of biodegradable materials, eliminating long-term medical waste and minimizing environmental impact. This positions SGSTD as a practical and sustainable alternative to nondegradable devices for widespread clinical use.

Despite these advantages, several limitations remain. Although SGSTD demonstrated weeklong drug release in large-animal (porcine) models, long-term in vivo studies are still necessary to evaluate potential effects of localized degradation and tissue-level exposure, which will be critical for guiding future clinical trials. While the device’s temperature-responsive release system is promising, its functional reliability in complex biological environments has yet to be established. Future work will explore remotely triggered thermal modulation to enable real-time, spatially precise control over drug release. Key challenges including heat dissipation, uniform thermal distribution, and biological responsiveness within the GI tract must be addressed to fully realize this capability. Additional investigations will also evaluate the formulation’s robustness under conditions simulating extended transport, storage, and refrigeration, which are key factors in eventual clinical translation.

In his 1959 lecture *There’s Plenty of Room at the Bottom*, Richard Feynman envisioned microscale systems operating within the human body, a concept SGSTD is now progressively bringing to life (*48*). Beyond insulin, this platform may support sustained oral delivery of diverse macromolecular therapies, including anti–tumor necrosis factor–α agents, anti–interleukin-6 receptor inhibitors, and monoclonal antibodies. Such applications are particularly relevant for chronic conditions like ulcerative colitis, Crohn’s disease, and systemic lupus erythematosus (*49*). The device’s microchannel architecture and external temperature tunability offer patient-specific drug release profiles, potentially transforming chronic disease management. Critically, SGSTD’s complete biodegradability aligns with emerging priorities in sustainable medicine. Its environmentally responsible design reduces long-term medical waste and lowers disposal costs, enabling sustainable deployment even in resource-constrained health systems. Ongoing refinement of degradation kinetics and waste processing strategies may further enhance its ecological footprint. As a versatile, cost-effective, and eco-conscious platform, SGSTD exemplifies a previously unknown class of oral medical devices poised to address global health and environmental challenges in parallel.

## MATERIALS AND METHODS

### Materials and chemicals

Unless otherwise stated, the materials and chemicals used in this study were purchased from Sigma-Aldrich, Greagent, or Macklin and were used without further purification. PLA (Natural PLA, Polymaker) was used in the preparation of the device shell and base. A range of materials were used in the preparation of the drug-loaded barbed needles, including gelatin, human recombinant insulin (Yeasen), PLA (USA), and chitosan. Purified water (Yibao, CRC), pure milk (Anchor, New Zealand), fruit juice (Suntory, Japan), canola oil (COFCO), and simulated gastric fluid (Genfeng) were used to aid in the in vitro simulation of the complex gastric environment. An amount of BaSO_4_ (Macklin) was used to assist imaging during x-ray fluoroscopy.

### Design and fabrication of SGSTD

SGSTD’s upper shell and gravity base were built in SolidWorks (Dassault Systemes, France) and subsequently converted into STL format. The model was sliced into G-code format using Cura 2018 (Ultimaker) software, and subsequently 3D-printed using a fused deposition modeling 3D printer (Ultimaker). The elastic strap, with dimensions of 3 mm (length) by 10 mm (width) by 0.3 mm (thickness), was fastened to the gravity base. The experiment involved the insertion of barb-shaped needles with microchannels into the holes of the cowl-type gelatine trigger. The barb-shaped needle was positioned at a specific height from the gravity base, as well as underneath the elastic strap. The barb-shaped needle’s lifting height and obstruction caused by the hood-type gelatine trigger provide a certain stored energy to the elastic strip, resulting in a favorable ejection for barb-shaped needle. Ultimately, the upper shell is securely attached to the gravitational base and subsequently sealed by a protective chitosan film at all the apertures of the device, resulting in an SGSTD.

### Self-orientation evaluation

The following experiments were performed to evaluate the behavior of different shaped devices in a simulated stomach environment. The sharp, low-slung, ellipsoidal, capsule, and SGSTD were released at an angle of 90° in a 3D-printed human stomach model to reach the lowest part of the stomach. The ex vivo porcine stomach tissue was fixed on a rotary shaker and the self-orientation time was recorded after rotating at 70 rpm for 20 s. The self-orientation time of the SGSTD was tested on an inclined 30°, 60°, and 90° platform. Initial and final self-orientation angles were measured on ex vivo porcine gastric tissue by releasing the device at 30°, 90°, and 180° angles. Last, five devices were released from 30°, 60°, and 90° angles on ex vivo porcine stomach tissues containing different fluids in a simulated stomach environment to record the self-orientation process and final angle.

### Retention effect evaluation

To assess retention stability under more complex mechanical loading, a long-duration dynamic flushing platform was established. Barb-shaped needles were vertically inserted into porcine gastric tissue with an initial angle of 90° relative to the tissue plane. Cyclic tissue deformation was generated using a mechanical steering servo to mimic gastric peristalsis, while test media were continuously flushed across the tissue surface at 60 ml/min. The tested media included water, milk, juice, oil, artificial gastric fluid, and a solid-liquid simulated chyme prepared by dispersing soft food-like particles in artificial gastric fluid. The assay was conducted for 60 min to provide sustained peristaltic-like loading and fluid shear; in the simulated chyme group, the solid particles further introduced frictional and collision loading. Images were recorded during the assay, and the final needle angle was quantified to evaluate retention stability under prolonged gastric-like mechanical disturbance.

### In vivo evaluation experiments

All procedures involving animals were conducted following the ethical guidelines and with the approval of the Animal Care Committee at Shenzhen Hospital, University of Chinese Academy of Sciences (no. LL-KT-2023-060) and Shenzhen University of Advanced Technology General Hospital (no. LL-KT-2026057). The experimental subjects consisted of randomly selected Bama pigs weighing around 35 to 40 kg. Before the experiment, the diet was modified to a liquid diet and the subjects refrained from eating overnight. Sedation was induced using atropine (Qsllour) and isoflurane (Ringpu). The heart rate of the experimental animals was monitored for stability using an electrocardiographic monitor (JH-8000VET, MINGRUITONG). The device was introduced into the pyloric area using an endoscope (XICHUAN, CMD-310A) while the stomach was expanded, and the movement of the device was observed. The entire treatment was devoid of any negative consequences, and the experimental pigs returned to their regular activities and diet within 6 hours following the procedure. More detailed experimental procedures are provided in the Supplementary Materials.

### X-ray imaging

To evaluate the retention time of SGSTD in vivo, x-ray imaging was conducted on experimental pigs administered SGSTD. Following anesthesia with isoflurane (Ringpu), the SGSTD, tagged with BaSO_4_ for radiographic visibility, was delivered into the esophagus via an endoscopic device. Images were acquired at preset intervals using a digital radiography system (Kampo Medical, DR71TK).

### Relative bioavailability assessment

To calculate the relative bioavailability (*F*_rel_) of SGSTD, the systemic exposure (AUC) of the drug at the same dose was compared between SGSTD administration and subcutaneous injection (reference standard). *F*_rel_ is the fraction of the area AUC under the plasma concentration-time curve for the two modes of administration, as follows: $F\text{rel}=\frac{{\left(\text{AUC}\right)}_{\text{SGSD}}\times D_{\text{SC}}}{{\left(\text{AUC}\right)}_{\text{SC}}\times D_{\text{SGSD}}}\times 100\%$, where ${\left(\text{AUC}\right)}_{\text{SGSD}}$ and ${\left(\text{AUC}\right)}_{\text{SC}}$ are the total area under the plasma concentration-time curve after SGSTD administration and subcutaneous injection, respectively, $D_{\text{SGSD}}$ and $D_{\text{SC}}$ are the dose size for SGSTD administration and subcutaneous injection administration, respectively.

### Compost biodegradation testing

An automated system was used to assess the biodegradability of the devices under controlled composting conditions. SGSTD samples were composted with an inoculum of vermiculite, garden soil, nutrients, and microbial broth (16:1 dry weight ratio) for 50 days at 58° ± 2°C in a 3-liter container, and bacterial cellulose was used to aid in degradation. Degradation rate was the percentage reduction in initial mass over time, where $M_{0}$ is the initial mass and $M_{t}$ is the mass after elapsed time. Data collection included CO_2_ emissions, mass changes, and environmental parameters, as follows: $D\;\left(\%\right)=\left(\frac{M_{0}-M_{t}}{M_{0}}\right)\times 100\%$, where $M_{0}$ is the initial mass of the sample and $M_{t}$ is the mass of the sample at time *t.* For soil microbiome analysis, soil DNA was extracted using the Mo-Bio PowerSoil DNA isolation kit according to the manufacturer’s protocol, and DNA quality was assessed by agarose gel electrophoresis. High-throughput sequencing of bacteria, actinomycetes, and fungi was then performed using biphasic PCR amplification and the Illumina NovaSeq 6000 platform.

### Statistical analysis

All the results presented are means ± SD. Statistical analysis was performed using two-sided Student’s *t* test and two-way analysis of variance (ANOVA) test with the software GraphPad Prism. The differences between experimental groups and control groups were considered significant at *P* < 0.05.

## Supplementary Materials

### The PDF file includes:

Supplementary Text Figs. S1 to S27 Tables S1 to S8 Legends for movies S1 to S15 References
