# Supplementary material for: A self-triggering and fully sustainable gastric device enabling long-acting oral delivery of macromolecules
Source: Sci Adv. 2026 Aug 28;12(35):eaef9186. doi: 10.1126/sciadv.aef9186 (PMC13524043; doi:10.1126/sciadv.aef9186)
Supplement: Supplementary file 1 [file sciadv.aef9186_sm.pdf]

Science Advances
AAAS

Supplementary Materials for

A self-triggering and fully sustainable gastric device enabling long-acting oral delivery of macromolecules

Chengbang Lu *et al.*

Corresponding author: Yiyuan Yang, yyy@nus.edu.sg; Xiangyu Liang, liangxiangyu@caas.cn

Sci. Adv. **12**, eaef9186 (2026)
DOI: 10.1126/sciadv.aef9186

The PDF file includes:

Supplementary Text
Figs. S1 to S27
Tables S1 to S8
Legends for movies S1 to S15
References

Other Supplementary Material for this manuscript includes the following:

Movies S1 to S15

SUPPLEMENTARY NOTE 1

Materials and chemicals

The materials and chemicals utilized in this investigation were procured from Sigma-Aldrich, Greagent, or Macklin, unless explicitly specified differently. These substances were employed in their original form without undergoing any further purifying procedures. The SGSTD housing and gravity base were SD-printed using fully-biodegradable polylactic acid (DL-PLA) filaments (made from corn starch). Polydimethylsiloxane (PDMS, Sylgard 184, Dow Corning, USA) was used to create the negative moulds of bioinspired millimeter needles. The preparation of barb-shaped needles involved the utilization of gelatine (made from pigskin, 250 g Bloom, Aladdin) combined with human recombinant insulin (Yeasen). PDLLA were dissolved in dichloromethane (CH_2Cl_2) and subsequently employed in the barb-shaped needle coating. Chitosan (100-200 mPaS) was utilized in the device protection module. The elastic drive module of SGSTD can be manufactured using biodegradable thermoplastic urethanes elastomer (TPU). Several studies were conducted to replicate the stomach environment, utilizing filtered water (Yibao, CRC), whole milk (Anchor, New Zealand), orange juice (Suntory, Japan), canola oil (COFCO), and artificial simulated gastric fluid (Genfeng).

SGSTD structure design and fabrication

SGSTD's upper shell and gravity base were built in SolidWorks (Dassault Systemes, France) and subsequently converted into STL format. The model was sliced into G-code format using Cura 2018 (Ultimaker) software, and subsequently 3D-printed using a fused deposition modelling (FDM) 3D printer (Ultimaker). The TPU elastic strap, with dimensions of 3 mm (length) × 10 mm (width) × 0.3 mm (thickness), was fastened to the gravity base. The experiment involved the insertion of barb-shaped barb-shaped needles with microchannels (*D*: 0.055 mm) into the holes of the cowl-type gelatine trigger. The barb-shaped needle was positioned at a specific height from the gravity base, as well as underneath the elastic strap. The barb-shaped needle's lifting height and obstruction caused by the hood-type gelatine trigger provides a certain stored energy to the elastic strip, resulting in a favourable ejection for barb-shaped needle. Ultimately, the upper shell is securely attached to the gravitational base and subsequently sealed by a protective chitosan film at all the apertures of the device, resulting a sustainable gastric self-triggered device (SGSTD).

We fabricated five different devices using similar method above, including Sharp, Low-slung, Ellipsoidal, Capsule, and SGSTD. The design parameters of those five devices are shown in Figure S2-3.

Geometric analysis optimisation

The distribution of curvature is directly related to the stress distribution and stability of the structure (*68*), and the curvature analysis is performed on the upper shell profiles of different devices, which in turn optimizes the shape of the device to explore the best stability. At any point x, the curvature K of the plane curve $f(\mathrm{x})$, the following *Equation* (1) is used:

$$K(x) = \frac{|f(\ddot{x})|}{(1+f(\dot{x})^2)^{\frac{3}{2}}} \tag{1}$$

where $f(\dot{x})$ is the first-order derivative of the curve, representing the slope;$f(\ddot{x})$is the second-order derivative of the curve, representing the rate of change of the slope; The curvature $K(x)$describes the degree of bending at the point x. The greater the curvature, the more drastically the curve bends at that point, and each curvature corresponds to a radius of curvature R. From the circle of curvature, the degree of curvature of the curve can be evaluated more intuitively, the following *Equation* (2) is used:

$$R = \frac{1}{K(x)} \tag{2}$$

R is the radius of curvature.

Center of mass calculation

In the realm of physics, the center of mass of a body represents the mean location of the mass distribution throughout the object (*69*). For a body with a continuous distribution of mass, the centroidal point can be derived via the volumetric integral of the product of the mass density function and the position vector across the body's volume, the following *Equation* (3) is used:

$$\vec{c} = \frac{1}{M}\int_V \underset{r}{\rightarrow} \rho\left(\underset{r}{\rightarrow}\right) dV \tag{3}$$

where $\vec{c}$ is the position vector of the center of mass, M is the total mass of the object, the following *Equation* (4) is used:

$$M = \int_V \rho\left(\underset{r}{\rightarrow}\right) dV \tag{4}$$

where $\underset{r}{\rightarrow}$ is the position vector from the origin of the reference coordinate system to the volume element dV, $\rho\left(\underset{r}{\rightarrow}\right)$ is the mass density function which describes the mass density at position $\underset{r}{\rightarrow}$, $dV$is the tiny volume element. This process can be effectively

accomplished by applying numerical methods to discretize the volume of the model into many small volume elements. The mass of each discrete element is determined by the product of its volume and the material density. Subsequently, the overall center of mass is calculated by summing up the mass moments of all volume elements (defined as the product of the mass of each element and its corresponding position vector), the following *Equation* (5) is used:

$$\vec{c} = \frac{1}{M}\sum_i \underset{r_i}{\rightarrow} \Delta m_i \tag{5}$$

where the mass of the ith volume element, is the position vector pointing to the ith volume element. The total mass M is the sum of the masses of all the volume elements.

Barb-shaped needle structure and preparation process

Degradable barb-shaped needles for insulin delivery were fabricated using an inverted-mold method. Briefly, barb-shaped needle templates were replicated in polydimethylsiloxane (PDMS) to obtain negative molds. A 35 wt% gelatin solution was mixed with human recombinant insulin powder at room temperature, cast into the PDMS molds, and dried at 2–15 °C to form the insulin-loaded barb-shaped needle cores. The needle cores were then coated with PDLLA solution and dried at 2–15 °C. The coating step was repeated to ensure complete and uniform surface coverage; Microchannels with a diameter of approximately 0.055 mm were subsequently generated in the PDLLA-coated barb-shaped needles by laser machining. All fabrication procedures were performed under dust-free and sterile conditions to maintain the quality and sterility of the needles.

Barb-shaped needle ejection system

Barb-shaped needle ejection system, akin to the mechanics of a bow and arrow, employs a degradable elastic strap (3 mm (length) × 10 mm (width) × 0.3 mm (thickness)) as the propulsive element. The elastic strap was inserted into dual channels on the SGSTD's gravity base and securely anchored with PDLLA blocks. This assembly ensured the elastic strap was firmly embedded within the grooved surface, reinforcing the SGSTD's reliability and structural integrity.

Protection design

In order to address the issue of premature activation of the device's elastic system, a protective strategy utilizing a chitosan film was engineered, which involved the utilization of a chitosan coating. A Petri dish was filled with a 0.5 wt.% chitosan solution and subjected to a heat drying at a temperature of 80 °C, resulting in the formation of a thin film. The film was subsequently trimmed to a size of 6 mm (length)

× 4 mm (width) × 0.03 mm (thickness) and securely attached to the lateral and basal openings of the device using a biocompatible glue, thus providing protection against accidental activation.

pH responsive ejection system

Given the nature of the barb-shaped needles, we employed a clever design that leveraged the interception capability of the hooded gelatine trigger to position the barb-shaped needles in a state of energy storage. The ejection of the needles was successfully intercepted by creating holes in the hooded gelatine that are slightly smaller than the diameter of the specific barb. Once an external acidic liquid permeates the apertures on both sides of the apparatus, the gelatine interceptor undergoes a progressive dissolution process, resulting in the immediate released and ejected of the intercepted barb-shaped needles, which are in a condition of energy storage, from the SGSTD.

SUPPLEMENTARY NOTE 2

Free-fall test *in-vitro* stomach model

To assess the ultimate outcomes of differently shaped devices during their free-fall within the stomach, experiments were performed using a simulation in a 3D-printed human stomach model. Diverse device shapes, including Sharp, Low-slung, Ellipsoidal, Capsule, and SGSTD, were individually released from the same position above the esophagus within the model, descending at a 90° angle (the angle between the device's central axis and the horizontal plane). This ensures that each device eventually reaches the lowest part of the stomach.

Post-disturbance stability assessment

To delve into the self-orientation effects of various as-designed devices under perturbing conditions *(55)*, *ex-vivo* pig gastric tissues (gastric corpus portion) with an area of approximately 80 cm^2 were employed. These tissues were secured to a rotating vibrator with a circular, highly transparent plastic container (*D*: 10 cm, *H*: 5 cm). The rotary shaker (DLAB, China) was rotated at 70 rpm for 20 s. After stopping the rotational motion, the self-orientation time of the devices was recorded. This process continued until the device came to a complete stop. Until the device stops moving, the angle formed by the center axis of the device and the horizontal plane is selected as the angle of final self-orientation, which is measured electronically by taking the final captured photographs.

Post-roll stability assessment

To assess the self-orientation time of SGSTD within gastric tissue, the SGSTD was released from fixed positions on platforms inclined at angles of 30°, 60°, and 90° relative to the plane of gastric tissue. The SGSTD was allowed to freely roll on the surface of the gastric tissue upon release. Timing commenced from the moment of SGSTD release and continued until it came to a halt. The angle greater than 75° formed between the SGSTD's central axis and the horizontal plane was considered to be an applicable self-orientation.

Post-free-fall stability assessment

To evaluate the falling effect of different shaped devices at various angles in gastric tissue *(27)*, *ex-vivo* porcine gastric tissue was used and uniformly coated with approximately 10 mL of artificial simulated gastric fluid. Devices of different shapes were positioned at a vertical distance of 15 cm and released freely at three different angles (30°, 90°, and 180°), allowing them to fall onto the liquid-covered gastric tissue. The initial drop angle (θ_0) was defined as the angle formed by the vertical line from the center axis of the device to the horizontal plane, and the final self-orientation angle (θ_f) was determined by the angle between the center axis of the device and the horizontal plane. These angles were measured electronically using the final captured photograph.

Self-orientation test in a simulated gastric environment

To investigate the self-orientation effects of different shaped devices within the gastric environment, Sony cameras were employed to record the self-orientation process at a frame rate of one frame per second. Five different devices were released from a fixed position at angles of 30°, 60°, and 90° relative to the platform containing gastric tissues, maintaining a vertical orientation relative to the plane. The dimensions of this platform were 30 cm (length) × 8 cm (width) × 2 cm (thickness). These devices freely rolled onto tissues containing approximately 10 mL of various liquids, including purified water, full-fat milk, orange juice, rapeseed oil, and artificial simulated gastric fluid. The *ex-vivo* gastric tissues were derived from the stomach of pigs, specifically, the gastric corpus and antrum sections, each dimension of 30 cm (length) × 8 cm (width) × 1.3 cm (thickness). It's worth noting that the *ex-vivo* pig stomach naturally retains the secretion of reconstituted mucus, eliminating the need for additional mucus supplementation. To establish a reference baseline for subsequent measurements of device orientation, a horizontal tissue platform was employed. After releasing the devices from their fixed positions, they freely rolled onto gastric tissues containing different liquids until coming to rest. The final self-orientation angle, formed between the device's central axis and the horizontal plane, was measured using captured photographs.

Evaluation of trigger time

Tests were performed using 20 mL of artificial gastric fluid (pH = 1.5) in order to assess the trigger mechanism's activation timing, which consists of protective films and interceptors. Chitosan film with a dimension of 6 mm (length) × 4 mm (width) × 0.03mm (thickness) and a hemispheric-shaped gelatin trigger with a diameter of 6 mm were placed in 20 mL Petri dishes. The dissolution process was timed to stop when the films and interceptors completely disintegrated and vanished.

Micro-computed tomography observation

To explore the effects of SGSTD on the tissue, non-destructive observations were performed on a high-resolution microcomputed tomography (micro-CT) imaging system (Bruker SkyScan 1172, Germany). The microcomputed tomography tests were performed in the device state after the barb-shaped needle was successfully ejected to puncture the surface of the isolated gastric tissue. The isolated gastric tissue (in which the barb-shaped needle had been inserted into the tissue) was fixed in a centrifuge tube for subsequent microcomputed tomography observation. The scanning parameters were set as follows: spatial resolution of 20 μm pixel size, X-ray tube voltage of 60 kV, X-ray tube current of 90 μA, no optical filter mode, scanning angle range of 180° and rotational step of 0.4°. The CT volume of the samples was reconstructed using the GPUReconServer software and pseudo-colors were added using the CTvox software.

SEM observation

To observe the microstructure of barb-shaped needle in more detail, an Field-Emission Scanning Electron Microscopy (FE-SEM, Merlin, Zeiss) were employed. The fabricated barb-shaped needle samples were firmly fixed on the sample stage by conductive adhesive, and then platinum sputtering was carried out using an ion sputtering device (Q150TES, Quorum), then the barbed microstructure, microchannel and degradable coating of barb-shaped needle samples were further observed using an FE-SEM microscope with an accelerating voltage of 5 kV and a current of 100 pA.

Nanoindentation test

A nanoindentation instrument (Hysitron TI950, USA) with a Berkovich needle tip (TI-0039, Bruker) was used to characterize the modulus of elasticity of random gelatin and gelatin-insulin samples (*70*). The maximum force was set to 1000 μN and the maximum depth was set to 5 μm. A flat sample with dimensions of 5 mm (length) × 5 mm (width) × 0.8 μm (thickness) was fixed to the sample stage using hot-melt adhesive, ensuring a tilting angle of less than 1°. The standard load function mode was selected and the test

results were calculated based on the force versus indentation depth curve as shown in Figure S9, the following *Equation* (6), (7) and (8) is used:

$$h_c = h - \varepsilon \frac{P}{S} \tag{6}$$

$$A = f(h_c) \tag{7}$$

$$E_r = \frac{\pi^{\frac{1}{2}} \times S}{2\beta \times A^{\frac{1}{2}}} \tag{8}$$

where h_c is the contact depth, h is the maximum indentation depth, ε is a correction constant (0.75), β is a constant (depending on the indenter), A is the surface area of the indentation, P is the loading force, E_r is the reduction modulus, and S is the contact stiffness. After the indentation test, the surface morphology and modulus distribution were studied using the built-in imaging module.

We used nanoindentation analysis to assess local plasticity variations between samples, as previously reported. The plasticity index (ψ) is used to characterize the plastic deformation properties of a material under external loading, the following *Equation* (9) is used:

$$\psi = \frac{A_1 - A_2}{A_1} \tag{9}$$

where A_1 and A_2 are the areas under the loading and unloading curves, respectively, as shown in Supplementary Figure S9. The closer the value of ψ is to 1, the greater the proportion of plastic deformation.

Mechanics simulation

All mechanics simulations in this study were conducted using the commercial finite element software ABAQUS 2020 (SIMULIA, France). In the software, we constructed models for the elastic strap, barbed needle, and biological tissue. These models were defined with experimentally obtained material parameters to ensure realistic simulation outcomes. To model the pH-responsive ejection system, we applied the Ogden hyperelastic model (*71*) as the constitutive model to accurately capture the nonlinear deformation behavior of soft tissue. The strain energy potential W in the Ogden model is given by the following *Equation* 10):

$$W = \sum_{i=1}^{N} \frac{\mu_i}{\alpha_i} \left(\lambda_1^{\alpha_i} + \lambda_2^{\alpha_i} + \lambda_3^{\alpha_i} - 3\right) + \sum_{i=1}^{N} \frac{1}{D_i} (J - 1)^{2i} \tag{10}$$

where μ_i and α_i are material parameters, λ_1, λ_2, and λ_3 represent the principal stretches, and$J$is the determinant of the deformation gradient. This model is particularly suitable for soft tissue, providing a precise representation of its mechanical response. For the

elastic strap, the accumulation and release of energy were critical to simulating the ejection process triggered by pH changes.

The elastic strain energy U_e stored in the strap was calculated as the following *Equation* (11):

$$U_e = \frac{1}{2}\int_V \sigma : \epsilon dV \quad (11)$$

where σ and ϵ denote stress and strain tensors, respectively, and V is the volume of the elastic strap. This energy calculation was essential for evaluating how the strap accumulates and releases energy, driving the needle's ejection as shown in Video S8.

For the retention simulation, we focused on assessing the effectiveness of different needle structures (e.g., barb-shaped, non-barbed, and column-shaped) in retaining within the tissue. The Von Mises yield criterion (*72*) was employed to model the plastic behavior of the barbed needle during insertion and retraction from the tissue. The equivalent stress σ_v is calculated as the following *Equation* (12):

$$\sigma = \sqrt{\frac{1}{2}[(\sigma_1 - \sigma_2)^2 + (\sigma_2 - \sigma_3)^2 + (\sigma_3 - \sigma_1)^2]} \quad (12)$$

where σ_1, σ_2, and σ_3 are the principal stresses. Yielding is assumed to occur when σ_x reaches the yield stressσ_y, allowing us to analyze how well the needle resists retraction forces.

To simulate the insertion and withdrawal of the barbed needle into the tissue and the instantaneous response upon ejection, we used ABAQUS 2020 to collect and analyse key mechanical parameters, including stress, strain, internal energy and velocity. This data helped us clearly quantify the retention force generated by the barbed needle during insertion and retraction as it interacts with the tissue. For example, for the retention force during insertion, we assessed the energy accumulation of the needle at different depths by analysing the change in internal energy, and combined with the stress and velocity data, accurately described the stress distribution characteristics at the contact surface between the needle and the tissue. These data further revealed the impact of needle design on the retention effect, allowing us to compare the retention effects of different needles under a variety of mechanical conditions, and in particular to analyse the retention ability of barbed structures during retraction.

We specifically evaluated the retention force of the barb-shaped needle during retraction as shown in Movie S10. The total energyE_k, reflecting the sum of energy involved in the needle's ejection and retention force during retraction, the following *Equation* (13) is used:

$$E_k = E_a + E_r \tag{13}$$

where E_a is the energy associated with the initial ejection force, and E_r represents the energy related to the retention force. This calculation allowed us to compare different needle designs' retention efficacy in tissue, particularly highlighting the effectiveness of barbed structures in maintaining position under pulling forces.

Insertion and extraction tests

To assess the feasibility of needles with a barbed structure, insertion and extraction tests were conducted on various *ex-vivo* pig gastric tissues (10 cm (length) × 10 cm (width) × 0.5 cm (thickness)), including sections from the gastric fundus, corpus, and antrum. These as-prepared tissues were affixed to a fixture with a central hole of 1.5 cm in diameter. The fixture, fabricated through 3D printing, was assembled and secured using 304 stainless steel hexagonal screws (D: 3 mm, L: 20 mm). The assembled fixture was affixed to the fixed end of mechanical universal testing machine (New Sansi, 6000 series, China). Concurrently, different needles (comprising barb-shaped, without barbed, and column-shaped) were affixed to a load cell and subjected to mechanical puncturing and pulling at a speed of 5 mm/min. The procedure commenced by lowering the device from a height of 1 cm above the tissue, followed by a tissue insertion of the barb-shaped needle. When a distinct inflection point appeared on the displacement-stress curve in the monitoring software (Multi-Channel Digital Closed-Loop Electronic Universal Measuring Hole System), the movement of the barb-shaped needle was halted. Subsequently, the needle was pulled upward at the same speed until a complete detachment from the tissue.

Retention effect in fluids

An improved adjustable-tilt model was used to evaluate the retention capability of barb-shaped needles under liquid flushing. Isolated porcine gastric tissue (30 cm × 9.5 cm) was fixed to the model with 304 stainless-steel screws, and barb-shaped needles were inserted to protrude approximately 1.5 mm from the tissue surface. Different liquids, including water, whole milk, orange juice, rapeseed oil, and artificial gastric fluid, were delivered using a peristaltic pump to simulate gastric flushing. The model was tilted at 30°, 60°, and 90°, and flushing was performed at 90 mL/min for 20 s. To assess the influence of flow rate, additional tests were conducted at 60, 90, and 120 mL/min under the same setup. Needle angular deviation relative to the tissue plane was quantified from images captured after flushing.

To further assess retention under more complex mechanical loading, a long-duration dynamic flushing platform was established. Barb-shaped needles were vertically

inserted into porcine gastric tissue with an initial angle of 90° relative to the tissue plane. Cyclic tissue deformation was generated by a mechanical steering servo to mimic gastric peristalsis, while test media were continuously flushed across the tissue surface at 60 mL/min. The tested media included the liquids described above and a solid–liquid simulated chyme prepared by dispersing soft food-like particles in artificial gastric fluid. The assay was performed for 60 min to provide sustained peristaltic-like loading and fluid shear; in the simulated chyme group, solid particles further introduced frictional and collision loading. Images were recorded during the test, and the final needle angle was quantified to evaluate retention stability under prolonged gastric-like mechanical disturbance.

Controlled slow release exploration of barb-shaped needles

To investigate the drug release effect of barb-shaped needles, a series with varying microchannel numbers (0, 1, 3, 5, 7, 9) were prepared. Methyl blue (0.5 g) was dissolved in 5 mL of 35% gelatin solution, molded into needles, air-dried, coated with PDLLA, and microchannels were fabricated. For release testing, needles were placed in PBS (pH = 7) at 37 °C, 70 rpm. Samples (1 mL) were collected hourly for 5 hours on day 1, then daily, with absorbance measured at 665 nm (NucleoTech). Following the microchannel-controlled release study, a temperature-controlled release experiment was conducted using barb-shaped needles with two microchannels. These needles were placed in PBS and incubated at 15, 37, 45, 55, and 65 °C under 70 rpm shaking. Samples were collected at set intervals to evaluate the effect of temperature on drug release.

Simulation of sustained drug release *in-vivo*

In this study, COMSOL Multiphysics was employed to numerically simulate the hypothetical diffusion and sustained-release dynamics of drugs within tissues in a barb-shaped needle drug delivery system. This approach aims to establish a theoretical model for controlling drug release. The simulation framework is based on the convection-diffusion equation (*73*), providing a conceptual basis for describing drug transport dynamics within biological tissue, the following *Equation* (14) is used:

$$\frac{\partial C_i}{\partial t} + \nabla \cdot J_i + u \cdot \nabla C_i = R_i \tag{14}$$

where $J_i = - D_i \nabla C_i$ represents the diffusion flux, D_i is the diffusion coefficient, u is the fluid velocity field, ∇ represents the gradient operator, and R_i is the chemical reaction term in Equation (14). Under the assumption that the main transport mechanism is diffusion, this equation can be further simplified to the following *Equation* (15):

$$\frac{\partial C_i}{\partial t} = \nabla \cdot (D_i \nabla C_i) \tag{15}$$

where C_i represents the concentration of species i, $\frac{\partial C_i}{\partial t}$ is the rate of change of concentration over time, D_i is the diffusion coefficient, and $\nabla \cdot (D_i \nabla C_i)$ describes the divergence of the diffusion flux, representing how the concentration spreads in space.

High-performance liquid chromatography test

To further evaluate the sustained-release behavior of insulin-loaded barb-shaped needles in a tissue-mimicking environment, agar hydrogel was used as a simplified hydrated tissue model. Recombinant human insulin was loaded into barb-shaped needles containing two microchannels, and the needles were inserted into preformed agar blocks. The agar samples containing inserted needles were maintained at 37 °C in an air-bath shaker at 70 rpm to simulate a hydrated and mildly dynamic tissue environment. At predetermined time points, insulin released into the agar matrix was extracted using ethanol. The extracts were centrifuged and filtered before HPLC analysis. Insulin concentration was quantified using high-performance liquid chromatography (HPLC, Agilent 1260, USA) equipped with a C18 column (250 × 4.0 mm, 5.0 μm particles). The mobile phase consisted of ACN:H_2O (60:40) containing 0.15% (v/v) trifluoroacetic acid, with a flow rate of 0.5 mL/min and UV detection at 214 nm.

Raman spectroscopy

Using a spectrometer (Renishaw) fitted with a 785 nm near-infrared laser, raman spectral data were obtained. Gelatin integration was required for sample preparation, which was then followed by ambient desiccation and curing. Spectroscopic analysis was conducted using an integration period of 15 s per spectrum, covering a spectral domain spanning from 400 to 4000 cm^{-1}. Each documented spectrum is the average of three successive scans to improve analytical accuracy.

High-resolution morphological observations

By using a confocal microscope (VK-X series, KEYENCE), we performed a high-resolution morphological study of barb-shaped needle after nanoindentation test. First, barb-shaped needle samples were placed on an optical stage and a 5 × objective lens was used to collect morphological information of the barb-shaped needle samples. Subsequently, the built-in VK Analyzer software was utilized to acquire 2D and 3D morphology images of the barb-shaped needle samples, along with the corresponding height map curves.

Cytotoxicity Evaluation

The cytotoxicity of five biomaterials (shell, barb-shaped needles, elastic strap, chitosan film, and gelatin support inner shell) on human gastric mucosal epithelial cells (*GES-1*) was evaluated using in-vitro 3-(4, 5-dimethylthiazolyl-2)-2, 5-diphenyltetrazolium bromide (MTT) assays. After UV sterilization, biomaterials were leached in RPMI1640 medium (0.01 g/mL) for an hour to generate extracts. Log-phase *GES-1* cells, seeded at a predetermined density in 96-well plates, were exposed to these extracts. Cell viability was quantified with the MTT assay, comparing the cytotoxic impact of each material against a control group (n = 3).

Additionally, the cytotoxicity of the shell was specifically assessed via a viability assay of live/dead staining. After ultraviolet (UV) sterilization and leaching in RPMI1640 medium, the extract was sterile-filtered (0.22 μm) and introduced to log-phase *GES-1* cells for 24-hour incubation at 37 °C in 5% CO_2. Viability post-treatment was visualized and quantified using confocal microscopy (Olympus, FV1200), combining both quantitative and qualitative measures to provide a comprehensive evaluation of material biocompatibility.

Biocompatibility Assessment

To determine the biocompatibility of barb-shaped needles with human gastric mucosal epithelial cells (*GES-1*), cultures were exposed to barb-shaped needle specimens over 1, 3, 5, and 7 days. Pre-exposure, barb-shaped needles were sterilized under UV light for 30 min and placed into 48-well plates. *GES-1* cells, seeded at 6 × 103 cells/well, were incubated at 37 °C in a 5% CO_2 atmosphere. Cell viability was assessed via the MTT assay at specified intervals. After incubation, the medium was removed, and wells were washed with PBS. Subsequently, MTT solution (0.5 mg/mL, 500 μL) was added to each well for 4 h, followed by dimethylsulfoxide (DMSO, 500 μL) to solubilize formazan crystals, with absorbance at 570 nm determining cell viability relative to controls (n = 3). In a separate assay, *GES-1* cells were directly seeded onto barb-shaped needle surfaces and cultured in a 5% CO_2 environment at 37 °C. On 7th day, cell labeling was performed using 4′, 6-diamidino-2-phenylindole (DAPI) for nuclear staining and fluorescent dyes for cytoskeletal visualization. Confocal microscopy (Olympus, FV1200) provided fluorescent imaging to analyze cell adhesion and proliferation on the barb-shaped needle surfaces.

X-ray observation

To further evaluate the retention time of SGSTD in the body, X-ray observations of experimental pigs taking SGSTD were conducted. Initially, the experimental pigs were anesthetized using isoflurane (Ringpu). Subsequently, the device (with a certain amount

of $BaSO_4$ (Macklin) attached internally to assist X-ray imaging) was delivered into the esophagus of the pig through an endoscope and then observed using a digital radiography system (Kampo Medical, DR71TK). To determine the residence time of SGSTD in the pig, X-ray observations were conducted at approximately 5-hour intervals after administration of SGSTD. It should be emphasized that the experimental pigs maintained normal feeding and defecation after administration.

In-vivo evaluation experiments

All procedures involving animals were conducted following the ethical guidelines and with the approval of Animal Care Committee at Shenzhen Hospital, University of Chinese Academy of Sciences (No. LL-KT-2023-060) and Shenzhen University of Advanced Technology General Hospital (No. LL-KT-2026057). To facilitate the smooth transportation and clear visualization of the device, experimental miniature pigs were allowed to adapt to the feeding environment for a week, then they were restricted to a liquid diet 24 h prior to experimentation and fasted overnight. In the *in vivo* trials, healthy Bama minipigs weighing approximately 35 kg were sedated with isoflurane (Ringpu, veterinary use). Each SGSTD contained a barb-shaped needle loaded with 0.5 mg of human recombinant insulin. Following confirmation of a stable heart rate using a cardiogram monitor (JH-8000VET, MINGRUITONG), the insulin-loaded SGSTD was delivered to the pyloric region using a gastroscope (XICHUAN, CMD-312) outfitted with a delivery mechanism in a pre-inflated stomach. The activation process of SGSTD was then monitored with a specialized gastroscope until the SGSTD and the barb-shaped needle were successfully separated. For the control group concerning administering the drug by direct injection, and human recombinant insulin solution with same dose (2 mL, 0.25 mg/mL) was administered subcutaneously. For the control group concerning oral drug delivery, a commercial capsule shell with same dose (0.5 mg) of human recombinant insulin were orally administered. For the blank control group, those experimental pigs were left untreated. Throughout the experimental procedures, no adverse reactions were observed, and all experimental pigs resumed feeding on a diet consisting of veterinary milk replacer and leafy green vegetables within 6 h post-operatively. At the end of the experiment, the equipment in the feces of piglets taking SGSTD was collected and photographed, followed by medical waste disposal.

EDG monitoring

To assess the post-anesthesia pig's cardiovascular system's reaction to external stimuli and ensure its safety, electrocardiogram (ECG) technology is employed to track its heart rate. To maximize the acquisition of the cardiac signals, the monitor (JH-8000VET, MINGRUITONG) and the ECG electrode sticker are positioned in the middle of the

chest and on either side of the ribs. The pigs' ears are fitted with pulse oxymoron sensors, which allow for real-time monitoring of blood oxygen saturation.

Assessment of drug exposure

After the experimental pigs receive the insulin delivery device and wake up safely from anesthesia without incident, the plan involves collecting venous blood for the study. To ensure a seamless sampling process, all experimental pigs are temporarily sedated with isoflurane (1～2 mg/kg) half an hour before sampling. For the first 5 h of the trial, blood samples were collected hourly from both experimental and control groups. Starting from the next day, blood samples were taken every 24 h for 7 days. After the blood sample was collected, it was left at room temperature for 10 to 15 min and centrifuged at 2 to 8 degrees. Blood samples were collected in tubes containing EDTAK2 anticoagulant (HUABO) and tubes containing separation gelling agent (HUABO) to ensure sample integrity and analysis accuracy. The samples were centrifuged at 3,000 g centrifugal force for 10 min and then placed in an incubator. The haematology department of Shenzhen Hospital of the University of Chinese Academy of Sciences assessed the insulin concentration, blood sugar levels and blood routine of each sample. During the blood assessment, close observation was maintained on the diet and bowel habits of all experimental pigs, while vigilance was maintained for any signs of discomfort or adverse reactions to SGSTD. When intervention is warranted, immediate action is taken.

Pharmacokinetic analysis

Based on pharmacokinetic analysis, systemic drug exposure (*AUC*), area under the moment curve (*AUMC*), relative bioavailability (F_{rel}), apparent clearance, and mean residence time (*MRT*) were calculated to further evaluate the sustained-release performance of SGSTD *in vivo*. Pharmacokinetic analyses were performed in experimental pigs receiving subcutaneous injection or SGSTD administration at the same insulin dose. Because subcutaneous injection was used as the reference in this study, the calculated bioavailability of SGSTD was defined as relative bioavailability (F_{rel}) (*74, 75*). The area under the blood drug concentration–time curve (*AUC*) was calculated using the trapezoidal method according to *Equation* (16):

$$AUC = \sum_{i=1}^{n} \left(\frac{C_i + C_{i+1}}{2}\right) \times (t_{i+1} - t_i) \tag{16}$$

where C_i and C_{i+1} are the blood concentrations at two consecutive time points, and t_{i+1} and t_i are the corresponding time points, respectively. Further, F_{rel} is the fraction of the area under the plasma concentration-time curve for the two modes of administration *(13)*, the following *Equation* (17) is used:

$$F_{rel} = \frac{(AUC)_{SGSD} \times D_{S.C}}{(AUC)_{S.C} \times D_{SGSD}} \times 100\% \quad (17)$$

where $D_{S.C}$ and D_{SGSD} represent the dose sizes for SGSTD administration and subcutaneous injection administration, respectively, and $(AUC)_{SGSD}$ and $(AUC)_{S.C}$ represent the total area under the plasma concentration-time curve following SGSTD administration and subcutaneous injection, respectively.

Apparent clearance was calculated as the administered dose divided by the *AUC* of the insulin concentration–time curve. Because SGSTD administration and subcutaneous injection are both non-intravenous routes and the systemic bioavailability (*F*) is unknown, this parameter represents apparent clearance rather than true systemic clearance. The following *Equation* (18) is used:

$$\text{Apparent clearance} = \frac{D_{dose}}{AUC_{0-\infty}} \quad (18)$$

where D_{dose} is the administered dose and $AUC_{0-\infty}$ is the overall exposure of the drug. Mean residence time of the drug in the body was measured by drug residence time, the following *Equation* (19) is used:

$$MRT = \frac{AUMC_{0-\infty}}{AUC_{0-\infty}} \quad (19)$$

where $AUC_{0-\infty}$ is the area under the drug concentration-time curve from the moment of administration to time infinity and consists of two components, AUC_{0-t} up to the last measured time point and $AUC_{t-\infty}$ from the last measured time point to infinity, reflecting the overall exposure to the drug, the following *Equation* (20), (21), and (22) is used:

$$AUC_{0-\infty} = AUC_{0-t} + AUC_{t-\infty} \quad (20)$$

$$AUC_{0-t} = \sum_{i=1}^{n-1} \frac{(C_i + C_{i+1})}{2} \times (t_{i+1} - t_i) \quad (21)$$

$$AUC_{t-\infty} = \frac{C_t}{\lambda_Z} \quad (22)$$

where C_t is the concentration at the last measurement time point t and λ_Z is the apparent terminal rate constant.

$AUMC_{0-\infty}$. represents the Area Under the Moment Curve (*AUMC*) from the start of drug administration to infinite time, which is related to the moments of the time-concentration curve of the drug in the body and consists of two parts $AUMC_{0-t}$.*and* $AUMC_{t-\infty}$, the following *Equation* (23) is used:

$$AUMC_{0-\infty} = AUMC_{0-t} + AUMC_{t-\infty} \quad (23)$$

$AUMC_{0-t}$ is calculated using the Trapezoidal Rule, where the area of each small trapezoid is multiplied by its time midpoint t_{mid} (i.e., the average of the two time points), the following *Equation* (24) is used:

$$AUMC_{0-t} = \sum_{i=1}^{n} \frac{(C_i+C_{i-1})\times(t_i-t_{i-1})\times(t_i+t_{i-1})}{2} \tag{24}$$

The computation of $AUMC_{t-\infty}$ from the last measured point t to infinity can be approximated by the following equation, the following *Equation* (25) is used:

$$AUMC_{t-\infty} = \frac{C_t \times t + \frac{C_t}{\lambda_Z}}{\lambda_Z} \tag{25}$$

Elimination rate constant is a pharmacokinetic indicator describing the rate of elimination of a drug from the body, using direct log linear regression, the data points of the elimination phase of a drug are selected, and their blood concentrations are logarithmically analysed by linear regression, where the negative slope of the regression line is the elimination rate constant λ_z, the following *Equation* (26) is used:

$$ln\,(C) = -\lambda_z \times t + ln\,(C_0) \tag{26}$$

where t is time, $ln\,(C)$is the natural logarithm of the drug concentration C, $ln\,(C_0)$ is the intercept of the fit, and C_0 is the drug concentration at $t = 0.$

Histological analysis

In histological experiments, stomach, heart, liver, spleen, lung and kidney tissue samples of euthanized animals were first fixed in 10% buffered formalin for more than 24 h. Subsequently, hematoxylin-eosin staining (HE) and Masson staining experiments were performed. The processed tissue samples were embedded in paraffin after gradient alcohol dehydration and cut into 3 ~ 4 μm thickness. These paraffin sections were stained histochemically with a stainer (Thermo, Gemini AS) and then dehydrated and sealed. Finally, histological observations and analysis were performed using a scanning slicer (3D, Pannoramic MIDI).

Fourier-transform infrared spectroscopy test

All samples were freeze-dried and ground with a 1:10 addition of potassium bromide support. Testing was conducted using a Fourier-transform infrared spectrometer (FTIR, Spectrum Two, PerkinElmer, USA).

Biodegradation assessment of post-use SGSTD

To assess the biodegradation performance of post-use SGSTD under controlled composting conditions, the experiment utilized the DKR-DA18 automated biodegradability testing system. The biodegradation capacity of SGSTD samples was determined by measuring the emission of carbon dioxide. Inoculum comprising vermiculite, nutritional soil, nutrients, and microbial broth was mixed with the samples at a dry weight ratio of 16:1. Samples were co-composted with 800 g of inoculum in 3L containers at a constant temperature of 58 ± 2 °C for a period of 50 days. Bacterial

cellulose is used as an auxiliary degradatio, where degradation rate (D) (*76*) is the percentage reduction in mass of the original mass as a function of time, the following *Equation* (27) is used:

$$D(\%) = \left(\frac{M_0 - M_t}{M_0}\right) \times 100\% \qquad (27)$$

where M_0 is the mass of the substance at the start of the experiment. M_t is the mass of the substance after a time period t.

Data collection included CO_2 emission, mass change, and environmental parameters, ensuring precision and reproducibility of results.

Soil microbiome analysis

In microbiome analyses, the initial step involves the extraction and quality assessment of DNA from samples. Fecal samples are processed using the QIAamp DNA Stool Mini Kit (QIAGEN, Hilden, Germany); soil samples are handled with the MO-BIO PowerSoil DNA Isolation Kit (Mo Bio Laboratories, Carlsbad, CA, USA); and swab or tissue samples are treated through a proteinase K digestion combined with phenol-chloroform extraction method. Subsequent quality verification of the extracted genomic DNA is conducted via 1.2% agarose gel electrophoresis. The amplification and sequencing of target sequences proceed through a biphasic PCR method. The initial amplification utilizes universal primers specific to the target regions, followed by selective recovery and purification of the target DNA fragments through gel electrophoresis. These fragments undergo a second round of PCR amplification incorporating barcodes, preparing them for high-throughput sequencing on the Illumina platform. This process employs the NovaSeq 6000 SP 500 Cycle Reagent Kit (Illumina, USA) to ensure high-quality data acquisition.

Soil DNA extraction

Genomic DNA extraction from soil samples is meticulously performed according to the MO-BIO PowerSoil DNA Isolation Kit (Mo Bio Laboratories, Carlsbad, CA, USA) protocol. Initially, 0.25 g of soil sample is introduced into PowerBead Tubes followed by gentle vortexing for homogenization. Subsequently, a defined volume of Solution C1 is added, and the mixture is heated at 60 °C until any precipitate fully dissolves. The sample then undergoes high-speed centrifugation to segregate DNA, followed by a series of solution treatments and centrifugation steps to eliminate contaminants, culminating in the purification of DNA. Critical throughout this procedure are the vortexing of samples, the addition of specific solutions, and centrifugation, ensuring the effective extraction and purification of DNA. The resultant DNA is immediately

suitable for subsequent high-throughput sequencing library preparation, obviating the need for further purification steps.

Incineration gas analysis

Samples are burned in a closed setup with oxygen supplied, and the resulting gases are collected through an exhaust into a gas bag. These gases are then filtered and analyzed using a quadrupole (NETZSCH, QMS403C) mass spectrometer.

Toxicological evaluation of incineration byproducts

Two experimental mice (25 g, random gender) were exposed to the gas environment collected following the incineration of SGSTD for a duration of 30 minutes, during which their behavioral characteristics were systematically recorded. Then, they were returned to their normal feeding environment. After 30 days, the two mice were euthanized. Subsequently, experimental autopsies were performed, including sample sampling of the heart, liver, spleen, lungs, and kidneys. These samples were fixed in 10% formalin, followed by HE staining and Masson staining experiments. Ultimately, pathological analysis was performed to check for any abnormalities.

Life-cycle assessment (LCA)

The usage of SimaPro Analyst 9.3.0.3 software and Ecoinvent 3.8 database complies with the specifications GB/T 24040-2008 and GB/T 24044-2008. Utilizing the ReCiPe 2016 Midpoint (H) V1.06 approach for Life Cycle Assessment (LCA) (*77*). The study used the kilogram as the operational unit, and the system boundary encompasses three stages: raw material production, transportation, and product production from cradle to gate. Environmental impacts are evaluated by analyzing input and output data related to the consumption of raw materials, distance and method of transportation, and production processes. The data selection concept disregards the environmental impact of less than 1% of the overall input and output, as long as the entire quantity does not surpass 5%. The environmental impact of producing raw materials for low-value trash is minimal, whereas the environmental impact of using recycled raw materials is estimated to be 50% lower than using alternative primary resources.

Environmental assessment of SGSTD applications

In order to evaluate the ecological consequences of SGSTD in the worldwide market, we developed an environmental impact assessment model utilizing IDF prediction data, T1D index, and Life Cycle Assessment (LCA) (*78, 79*). The model postulates that individuals diagnosed with type 1 diabetes necessitate daily administration of insulin injections. Due to the prolonged duration of action of SGSTD (7 days), each patient

required an average of 53 SGSTD devices each year, resulting in a reduction of about 313 devices, or 85.7%, compared to the previous requirement of 365 disposable syringes per year. We employ the demand growth model (*Sigmoid curve)* (*80*), in conjunction with market penetration and market capacity data, to conduct a quantitative analysis of the projected market demand in the upcoming years.

Initially, we ascertain the market capacity of each country, which is established by the quantity of patients and the projected demand for that particular year. To forecast market demand using the demand growth model (*Sigmoid curve*), the following *Equation* (28) is used:

$$D(t) = K \times \left(\frac{1}{1+e^{-r(t-t_m)}}\right) \quad (28)$$

D(t) represents the demand at a specific time, *K* represents the maximum market capacity, r represents the constant rate of increase, t_m represents the time when the demand reaches its highest point, and t represents the time period between the base year and the forecast year.

Considering that the SGSTD treatment involves both biodegradation and incineration, we utilized comprehensive consumption data to compute the environmental consequences, such as global warming potential (GWP) and acidification potential (AP), of disposable syringes and SGSTD utilization in patients diagnosed with type 1 diabetes. We conducted a thorough analysis comparing the environmental effects of regular medical disposable syringes with SGSTD. Our evaluation focused on the ability of SGSTD to effectively reduce carbon emissions and soil acidification. An analysis was conducted to evaluate the environmental improvements and examine the prevalence and growth rates of diabetes in eight regions of the world in 2040. In order to assess the many indirect economic advantages, we conduct a simulation to determine the cost savings for countries that have implemented an Emissions Trading System (ETS) (*81*). This analysis is based on the weighted carbon pricing of these nations. Ultimately, the model assesses the global prevalence of type 1 diabetes in 2040, ranks the number of affected individuals, identifies the top 10 nations with the highest prevalence, and thoroughly examines the impact of SGSTD on reducing the risk of developing the disease, taking into account income levels.

Utilize R Studio to graphically represent data on GWP and AP improvement, and display the data on a global scale using the r natural earth package. The code files can be found at following website: https://github.com/domelxb/Environment-assessment-of-SGSTD-application.

Photograph and video recording

In the absence of specific instructions to the contrary, all video recordings and photographic documentation in this research were executed using a high-resolution camera (SONY, A7R IV, Japan) coupled with a zoom lens.

Statistical analysis

All presented results are expressed as mean values ± standard deviation. Statistical analyses were conducted using Origin 2022 software, employing two-tailed Student's *t*-tests or analysis of variance (ANOVA). Results were considered statistically significant at $P < 0.05$.

SUPPLEMENTARY FIGURES

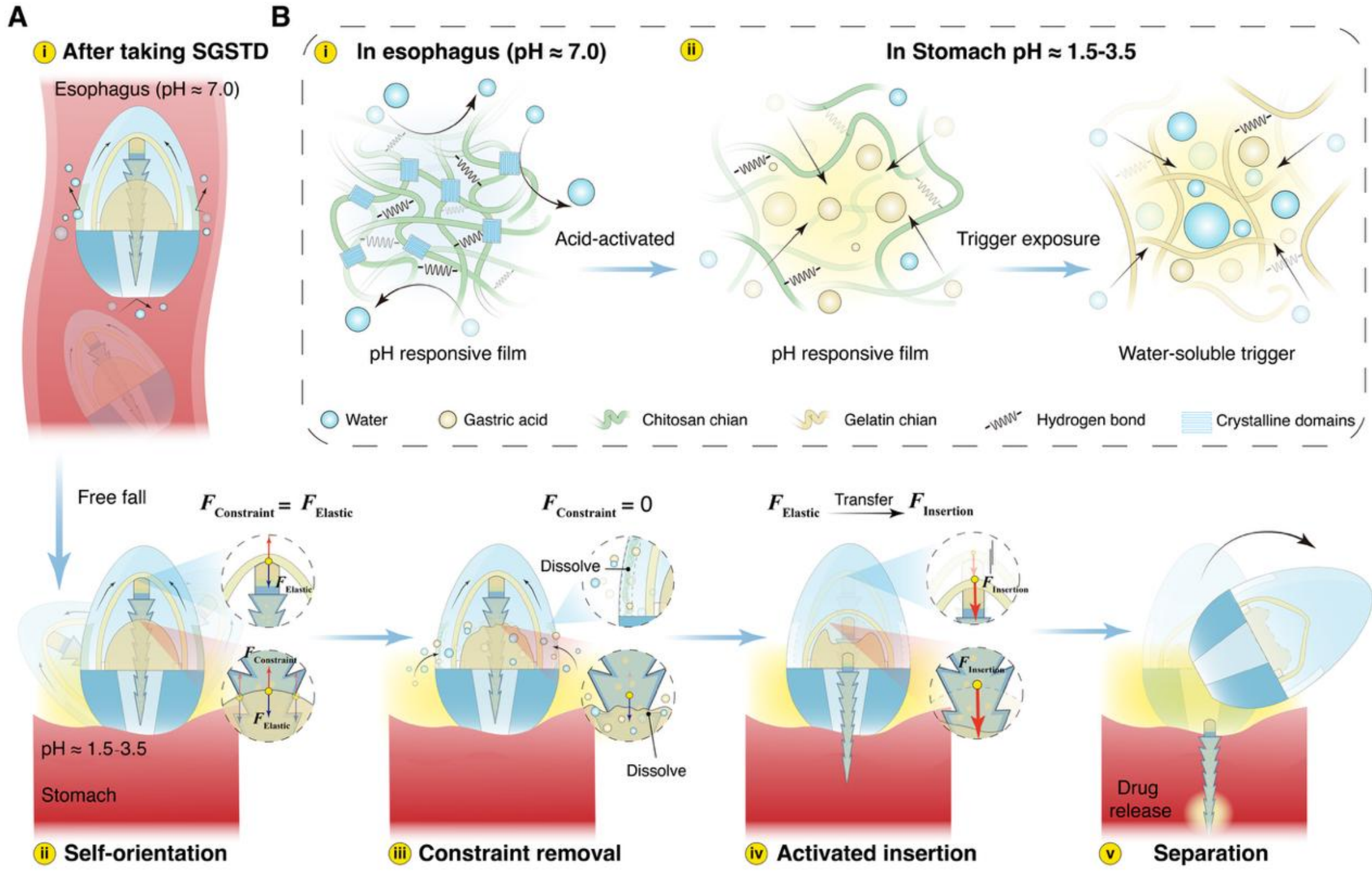

Fig. S1. The entire operation process and self-triggering mechanism of SGSTD. (A) Schematic of the self-triggered deployment of SGSTD after oral administration, showing esophageal transit, gastric self-orientation, acid-triggered constraint removal, activated insertion of the barb-shaped needle, and subsequent device–needle separation for sustained drug release. (B) Molecular and structural basis of acid-triggered constraint removal. The chitosan-based pH-responsive film remains stable in the near-neutral esophagus but dissolves in the acidic stomach, exposing the gelatin-based trigger, which subsequently swells and disintegrates to release the mechanical constraint for needle deployment.

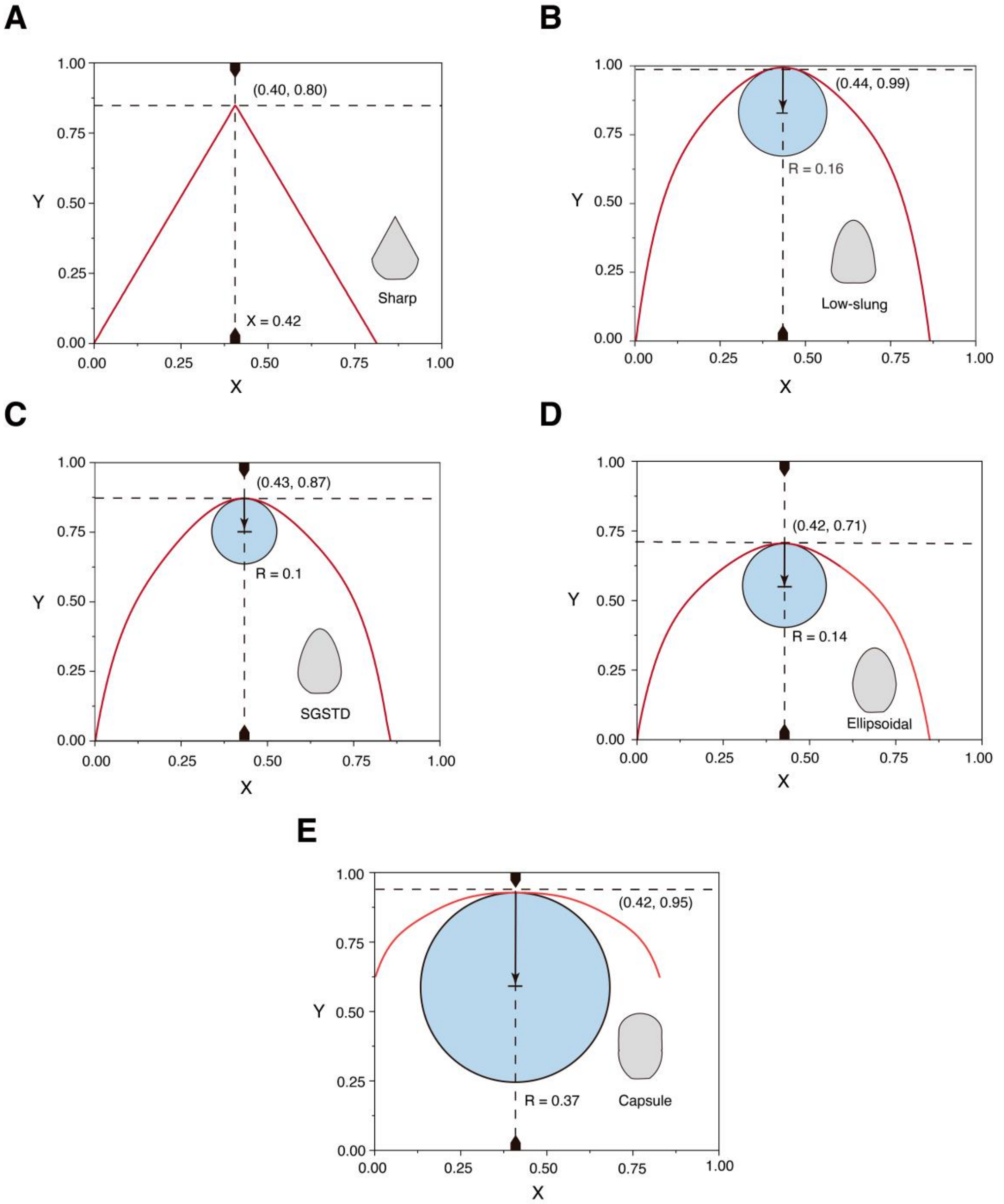

Fig. S2. The optimization function depicted reveals that among the five device shapes. (A-E) Five device optimization functions with different curvature profiles, where SGSTD model, with its minimal curvature circle radius of $R = 0.1$, demonstrates an enhanced self-orienting effect. This diminutive radius signifies an optimally positioned center of mass that efficiently harnesses torque generated by rotational disturbances during descent. The torque thus provides a restorative force that pivots the device towards an upright orientation. Consequently, the minimal radius of the curvature circle, indicative of a low and centralized mass distribution, correlates directly with the device's autonomous postural correction capability, thereby ensuring stable reorientation under the influence of gravity. The relevant parameters of the specific model functions can be found in **Table S2- S5.**

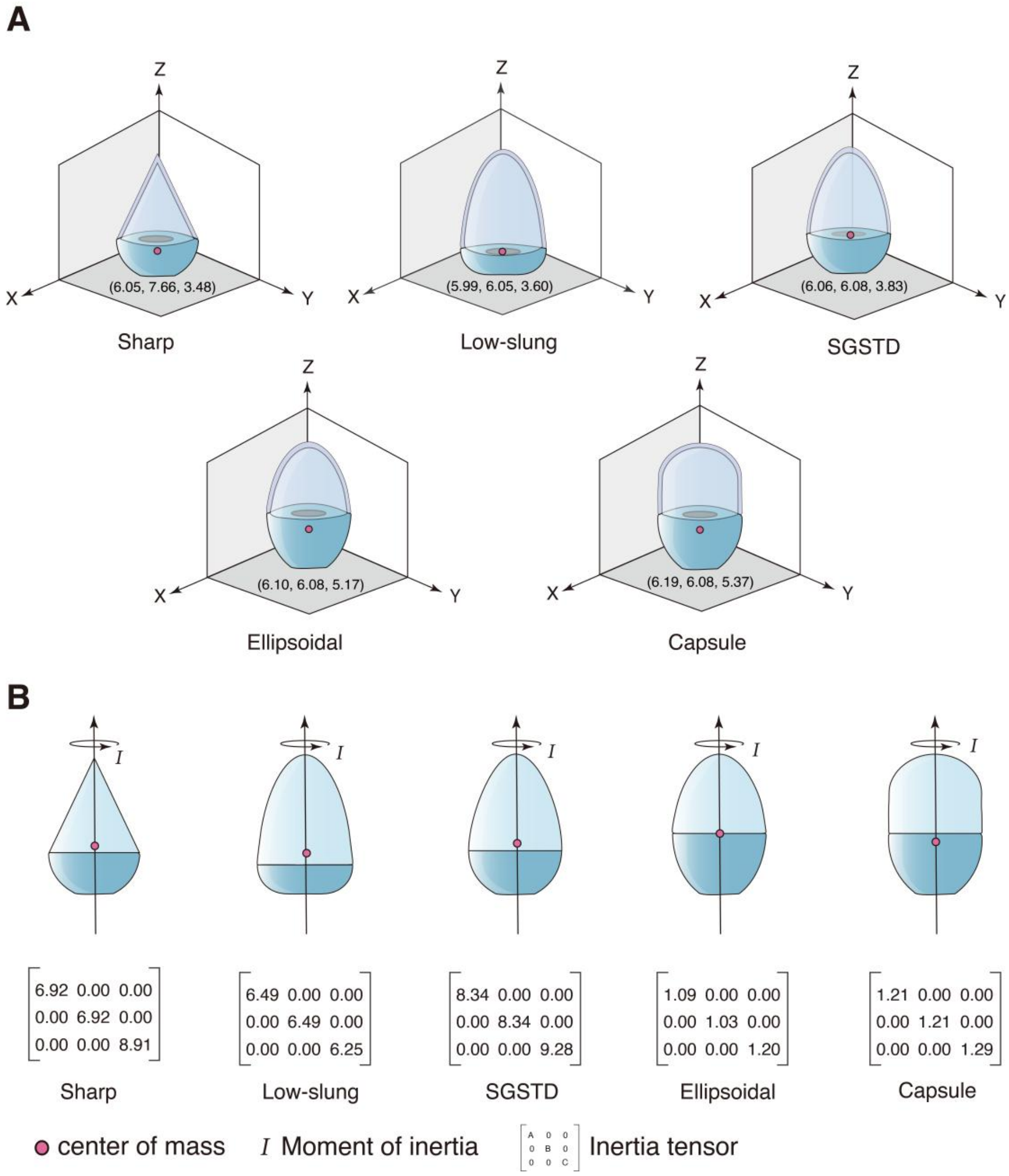

Fig. S3. Comprehensive analysis of the gravitational centers and moments of inertia for devices with varied shapes. (A) Spatial center of gravity distributions within a three-dimensional coordinate system. (B) Inertia tensor analysis for diverse device shapes, SGSTD is characterized by a substantial inertia tensor with diagonal values (I_{xx} = 8.34, I_{yy} = 8.34, I_{zz} = 9.28), indicating a high moment of inertia relative to the other device shapes. This enhanced moment of inertia implies that the SGSTD has a greater resistance to angular acceleration, providing it with superior stability. The as-designed model parameters in details are provided in Table S6.

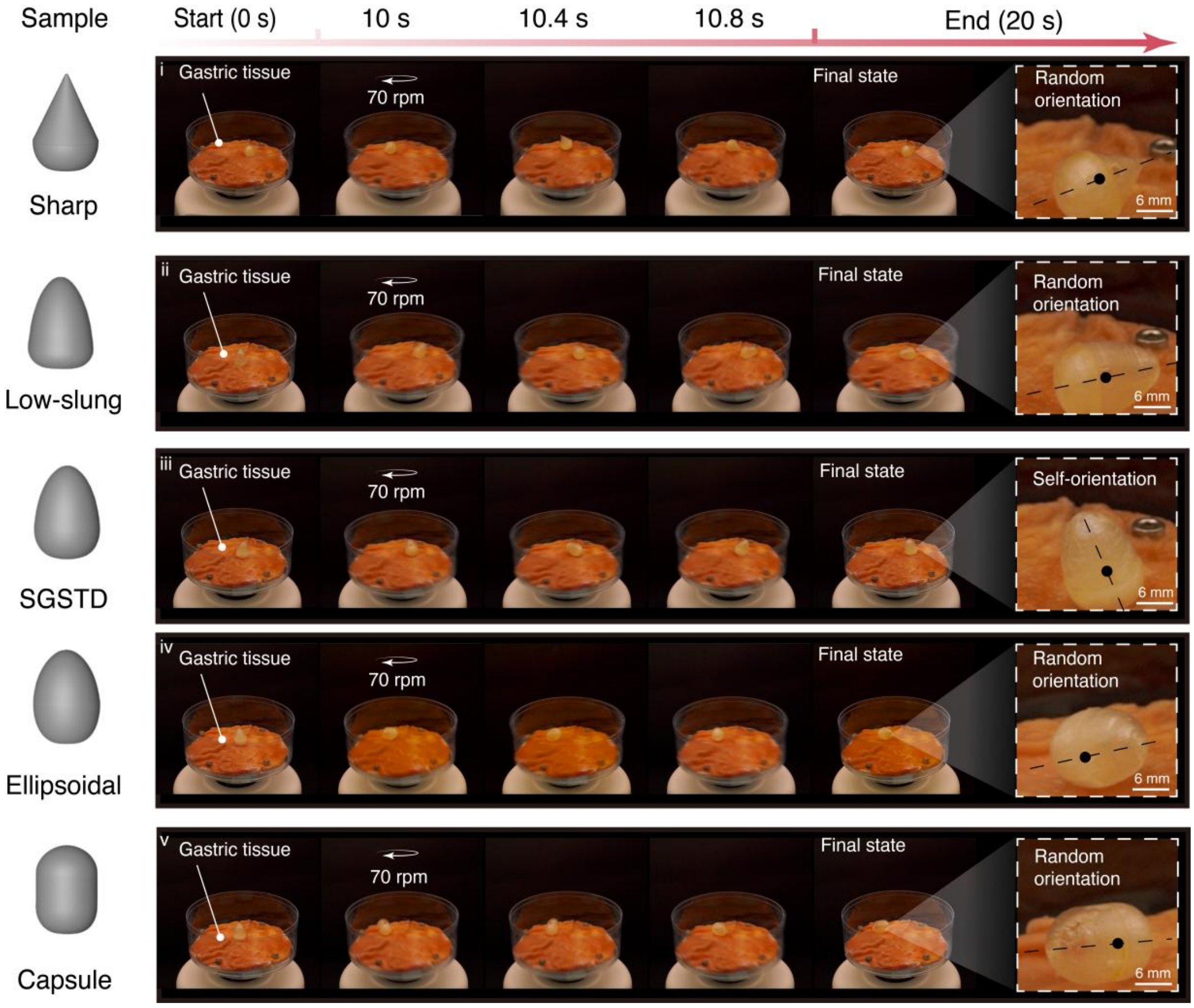

Fig. S4. Self-orientation performance of different geometries under dynamic gastric conditions. Representative time-lapse images showing the behavior of five sample geometries (Sharp, Low-slung, SGSTD, Ellipsoidal, Capsule) placed vertically at 0 s in a simulated gastric tissue environment rotating at 70 rpm. All samples were subjected to mechanical perturbation for 20 s. Among them, only the SGSTD geometry exhibited self-orientation, actively adjusting its posture to align a specific side upward. In contrast, the other geometries maintained random orientations, indicating a lack of self-alignment ability. Insets on the right show the final orientation of each sample at 20 s with directional markers. Scale bar: 6 mm.

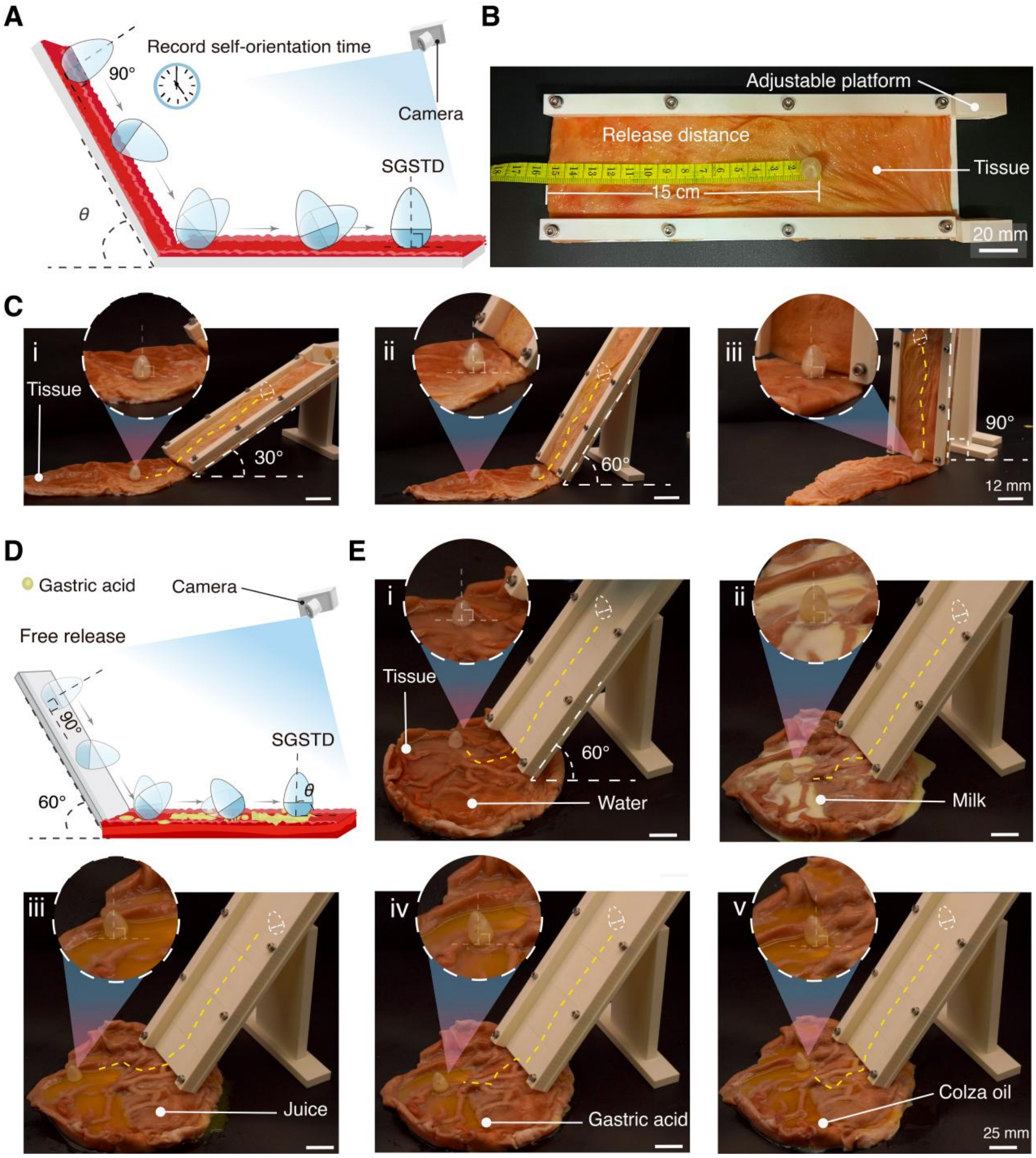

Fig. S5. Self-orientation effect of SGSTD in simulated gastric scenarios. (A) Self-orientation time test of SGSTD released from different platform angles onto tissue surface. (B) Preset release distance of 15 cm for SGSTD. (C) (i-iii) The final self-orientation state of SGSTD after being released from different platform angles (30°, 60°, and 90°). (D) Schematic representation of SGSTD being freely released onto tissue covered with gastric acid at a platform angle of 60°. (E) (i-v) The self-orientation of SGSTD as it rolls onto tissue covered with different liquids (water, milk, juice, gastric acid, and colza oil) from a platform angle of 60°, with the yellow line indicating the motion trajectory.

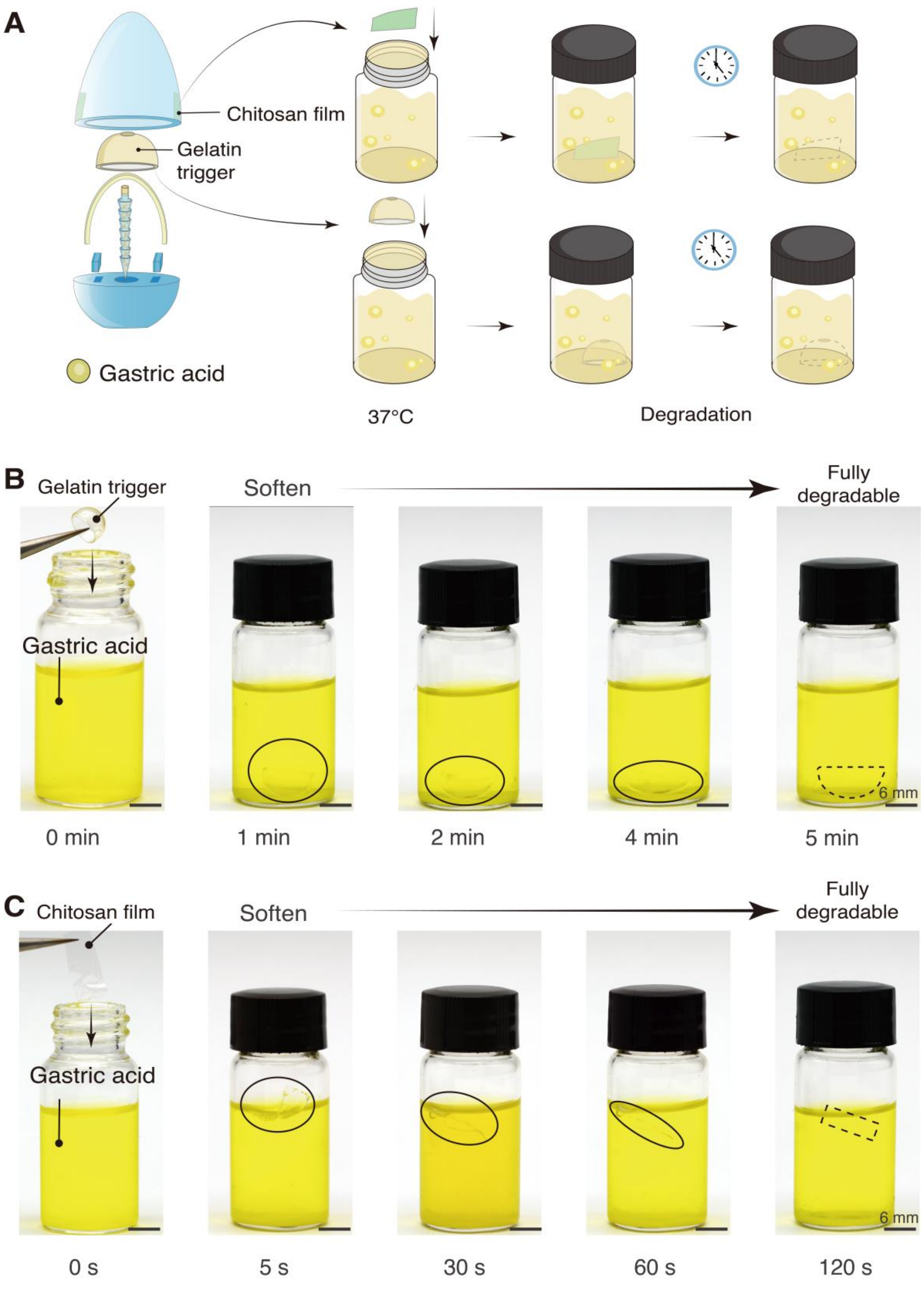

Fig. S6. Degradation of protective films and trigger in artificial gastric acid. (A) Schematic representation of the degradation of the trigger and protective film in artificial gastric acid. (B-C) Representative images of trigger and protective film after immersion in artificial gastric acid for different times, where the solid white coils are the incompletely degraded trigger and the protective film, and the white dashed coils indicate that both have completely disappeared in the artificial gastric acid. Scale bar: 6 mm.

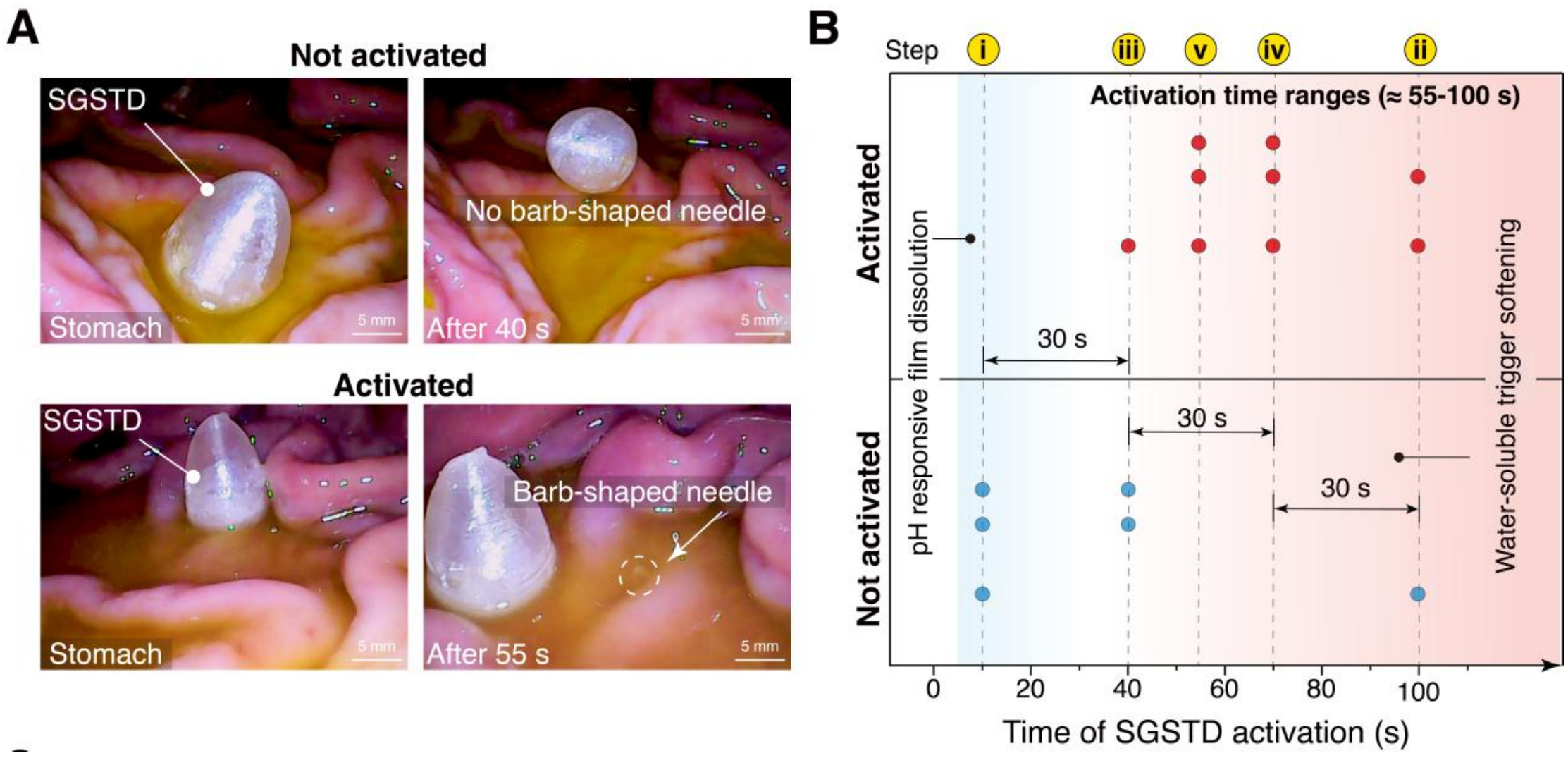

Fig. S7. Evaluation of SGSTD activation timing and gastric retention. (A) Representative endoscopic images showing unactivated and activated states of SGSTD in the stomach. (B) Interval-bracketing assessment of activation time. Based on stepwise endoscopic observations at predefined time points, SGSTD activation was estimated to occur mainly within 55–100 s after administration. The blue and red dots indicate non-activated and activated outcomes, respectively.

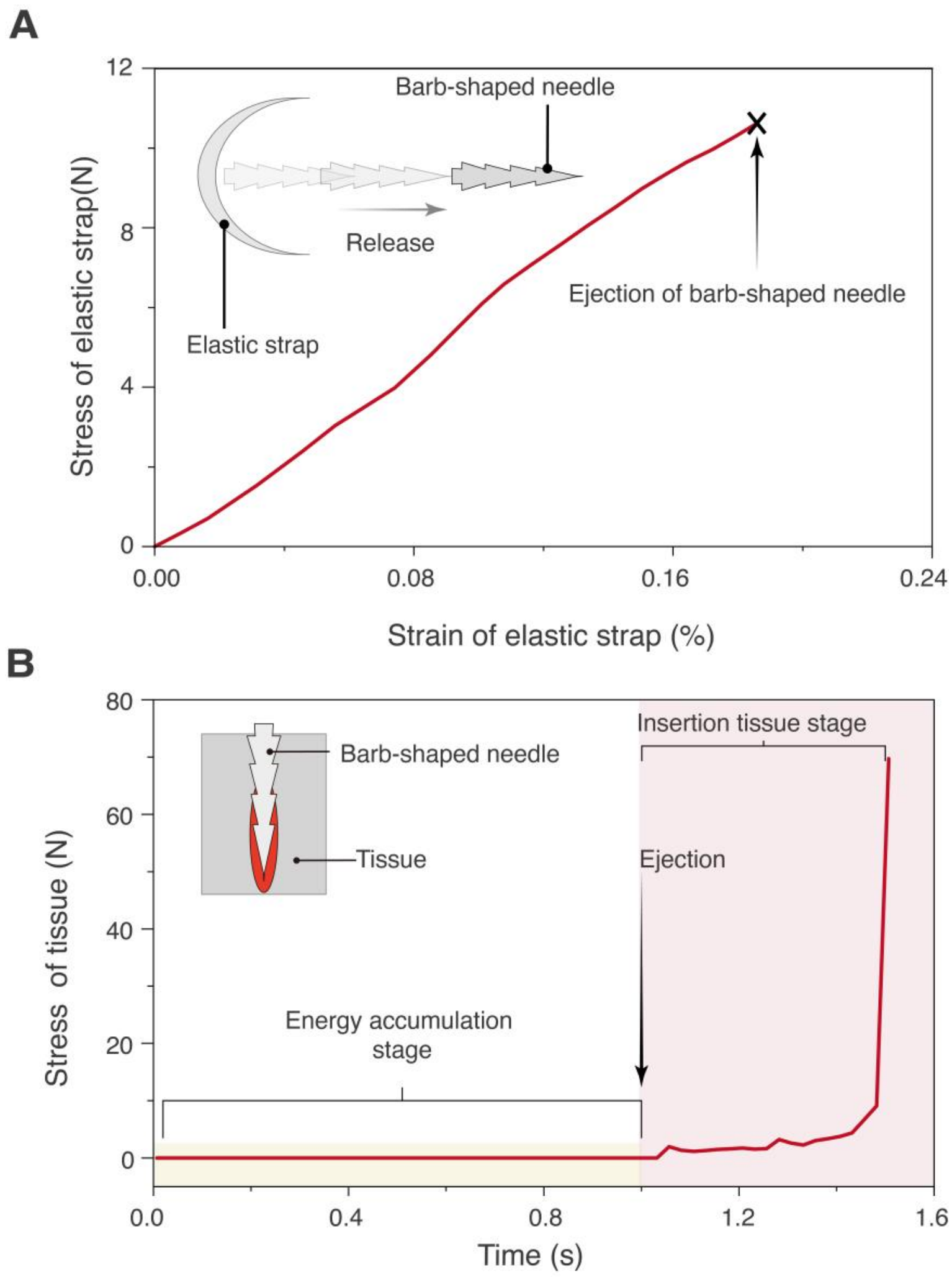

Fig. S8. Finite element simulation of barb-shaped needle ejection process. (A) Stress-strain curve of the elastic strap during the energy storage phase, with an inset illustrating the moment of barb-shaped needle release. (B) Stress evolution in the tissue after barb-shaped needle insertion, showing the different phases, including energy accumulation, insertion, and ejection stages. The inset provides a schematic representation of the barb-shaped needle insertion into the tissue, illustrating the resulting stress distribution during the simulated process.

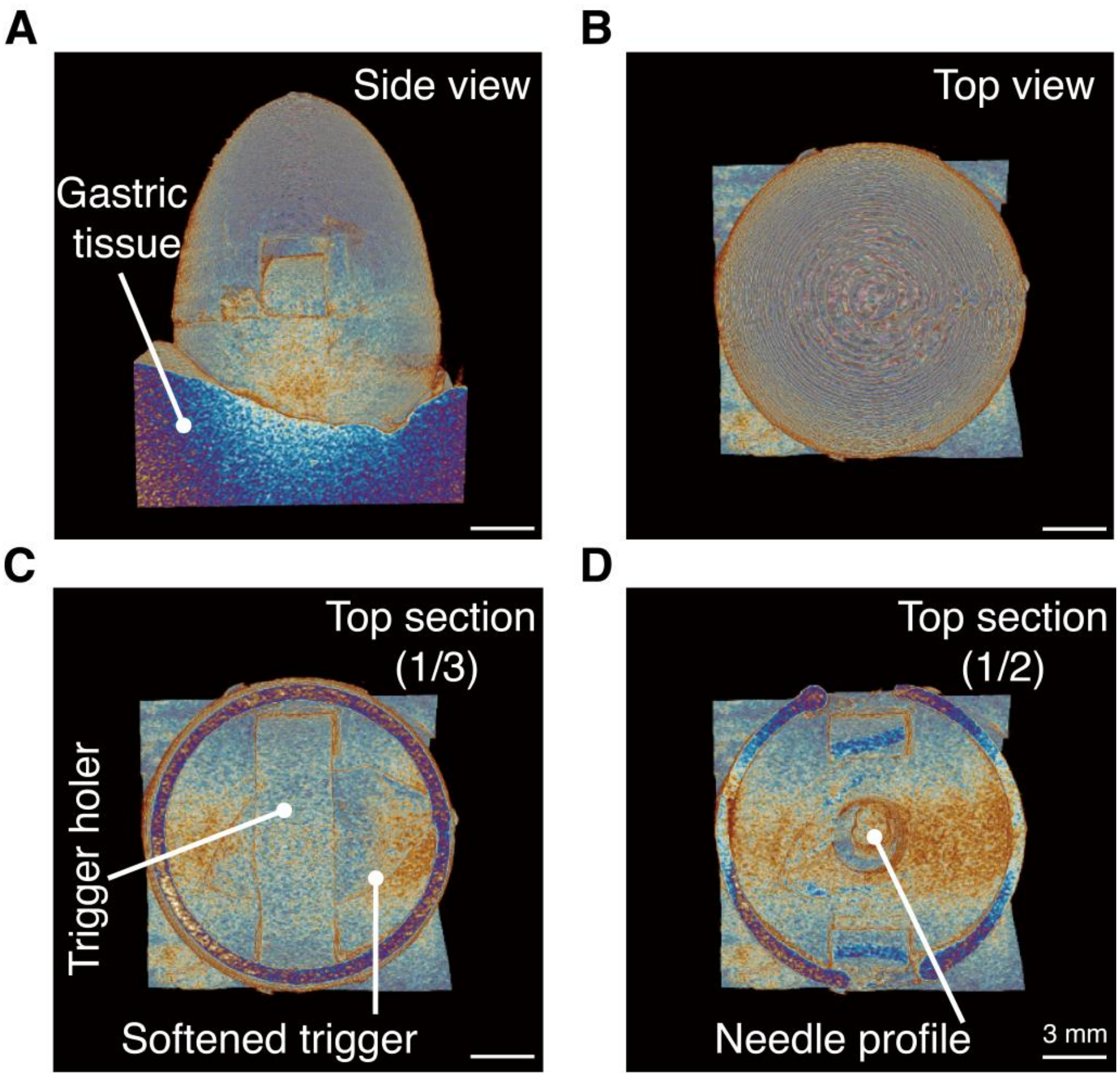

Fig. S9. High-resolution morphological analysis of SGSTD on gastric tissue using CT imaging. (A) Side-view of SGSTD on gastric tissue, showing the interaction between the barb-shaped needle and tissue. (B) Top-view of SGSTD showing the overall structure. (C) Cross-sectional top view at one-third depth, highlighting the softened trigger and trigger holder. (D) Cross-sectional top view at half-depth, showing the profile of the barb-shaped needle and its positioning relative to the gastric tissue. Scale bar: 3 mm.

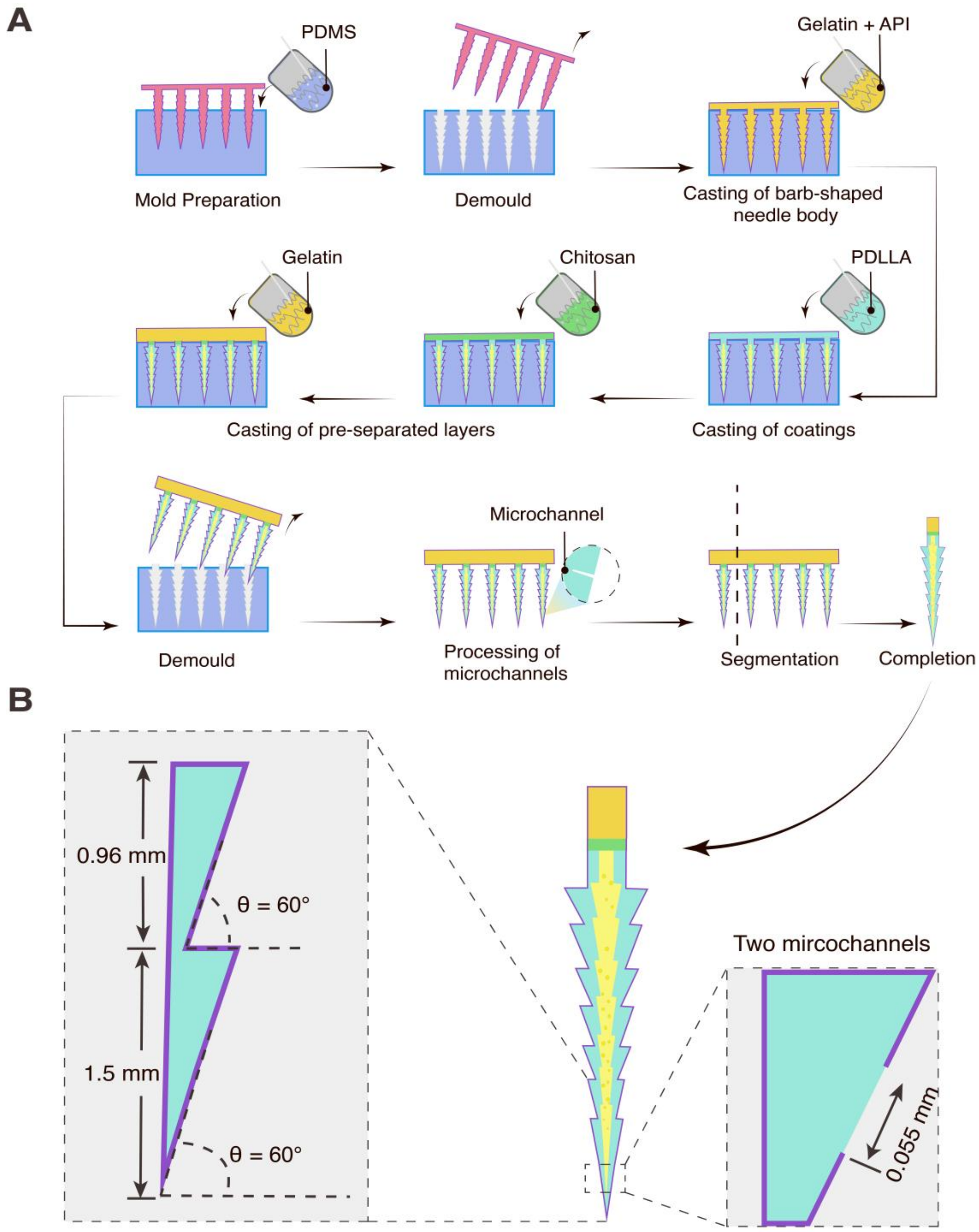

Fig. S10. Design parameters and fabrication process of barb-shaped needles. (A) Complete preparation process of drug-carrying needles with barbs. (B) Barb-shaped needle with two micro-channels in a diameter of 0.055 mm.

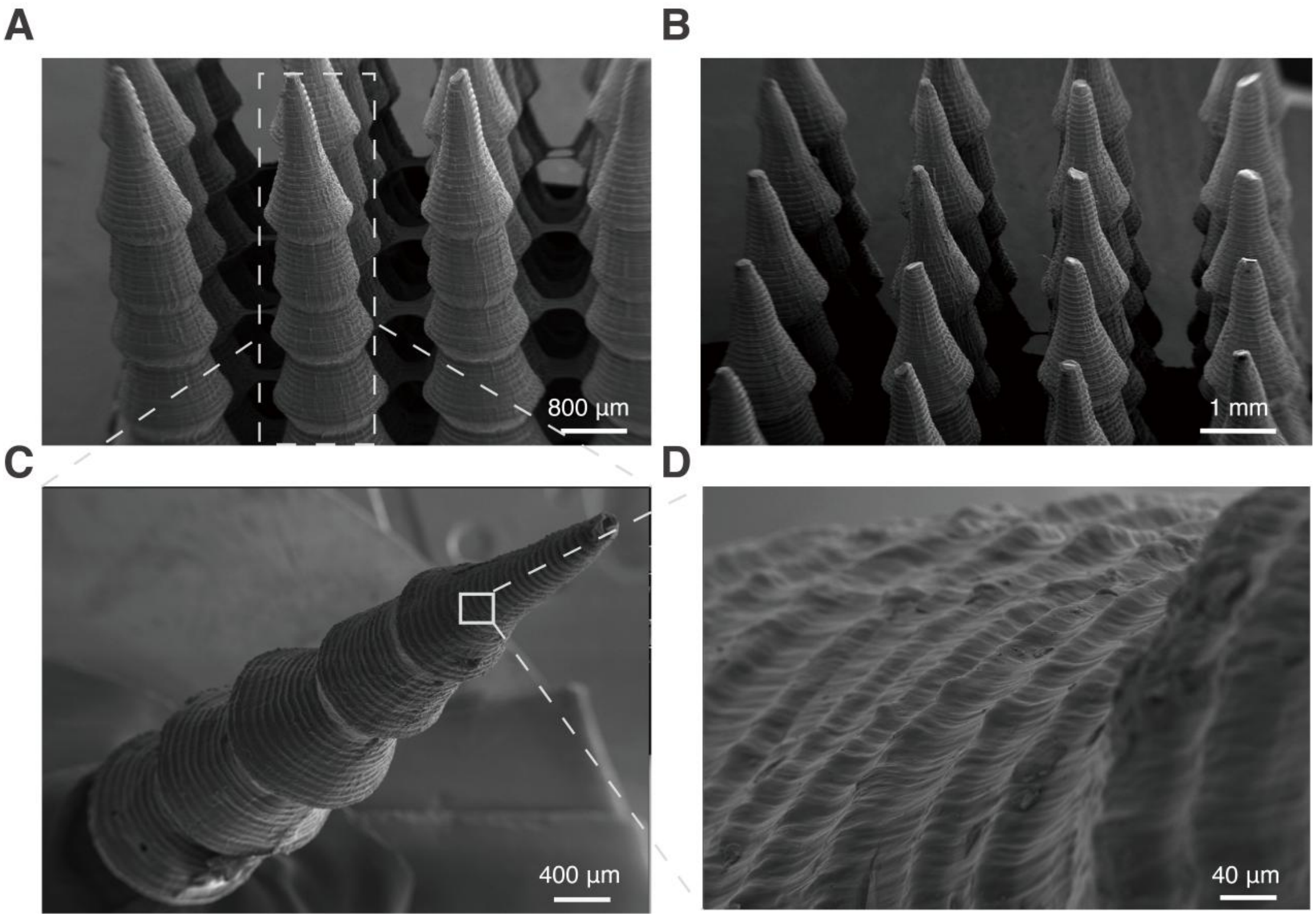

Fig. S11. SEM images of barb-shaped needles. (A) Oblique angle view and (B) the top of the barb-shaped needle array. (C) Barb-shaped design for effective skin retention. (D) High-magnification view of the barb-shaped needle surface.

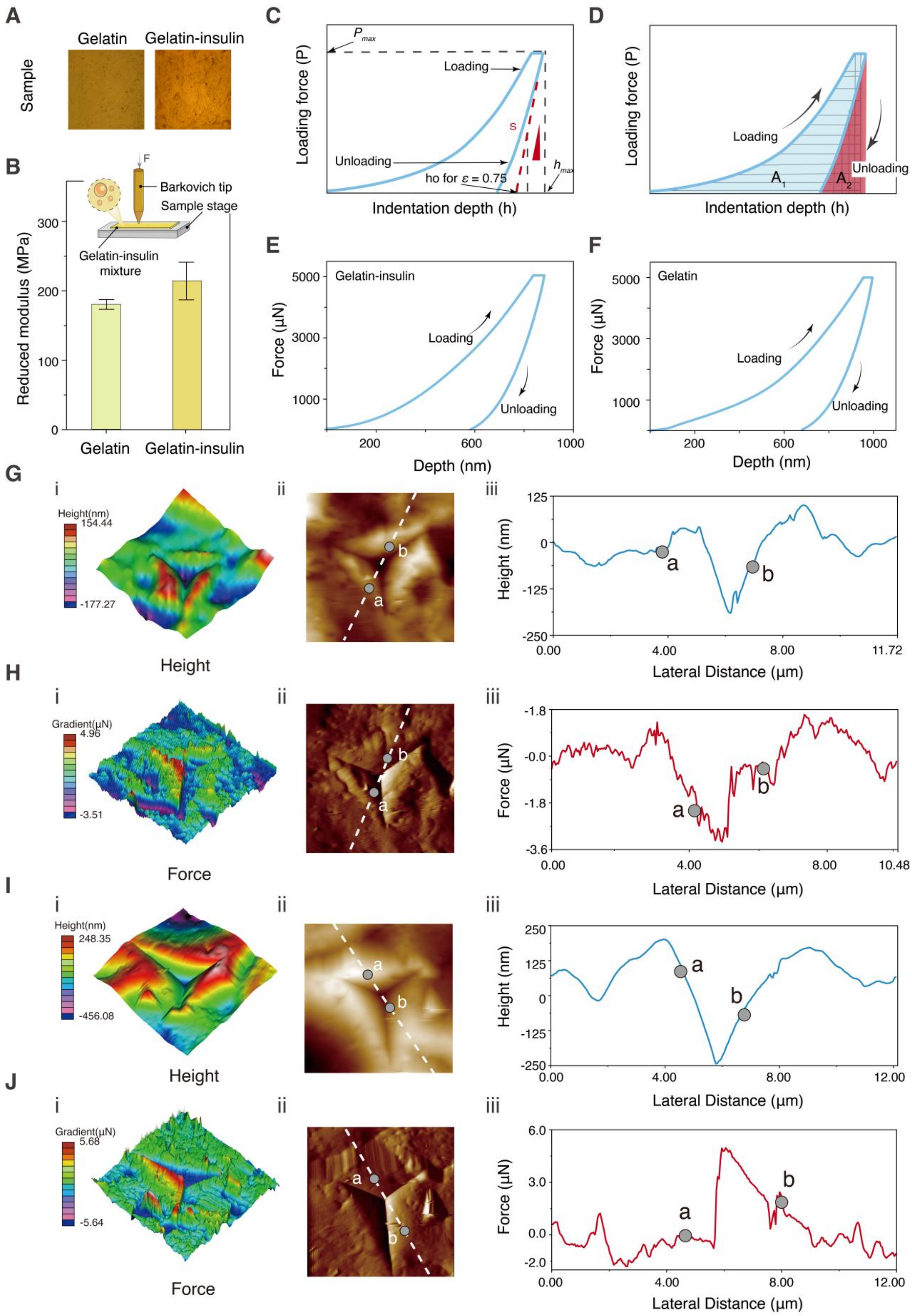

Fig. S12. Nanoindentation analysis of gelatin and gelatin-insulin mixture. (A-B) Morphology of gelatin and gelatin-insulin samples, with bar charts showing the reduced modulus (MPa) of both samples obtained from nanoindentation tests. (C-D) Evaluation of representative plasticity by nanoindentation curves, with insets depicting schematic representations of the indentation process. (E-F) Representative force-displacement

curves for gelatin and gelatin-insulin samples during Berkovich tip loading and unloading. (G-J) 3D height distribution maps and force distribution maps of the surfaces of gelatin and gelatin-insulin obtained from nanoindentation tests. Representative images of surface morphology and the corresponding height profiles along the constant tapping distance of 5 μm (marked by the white dashed line) are shown, along with their respective force mapping curves.

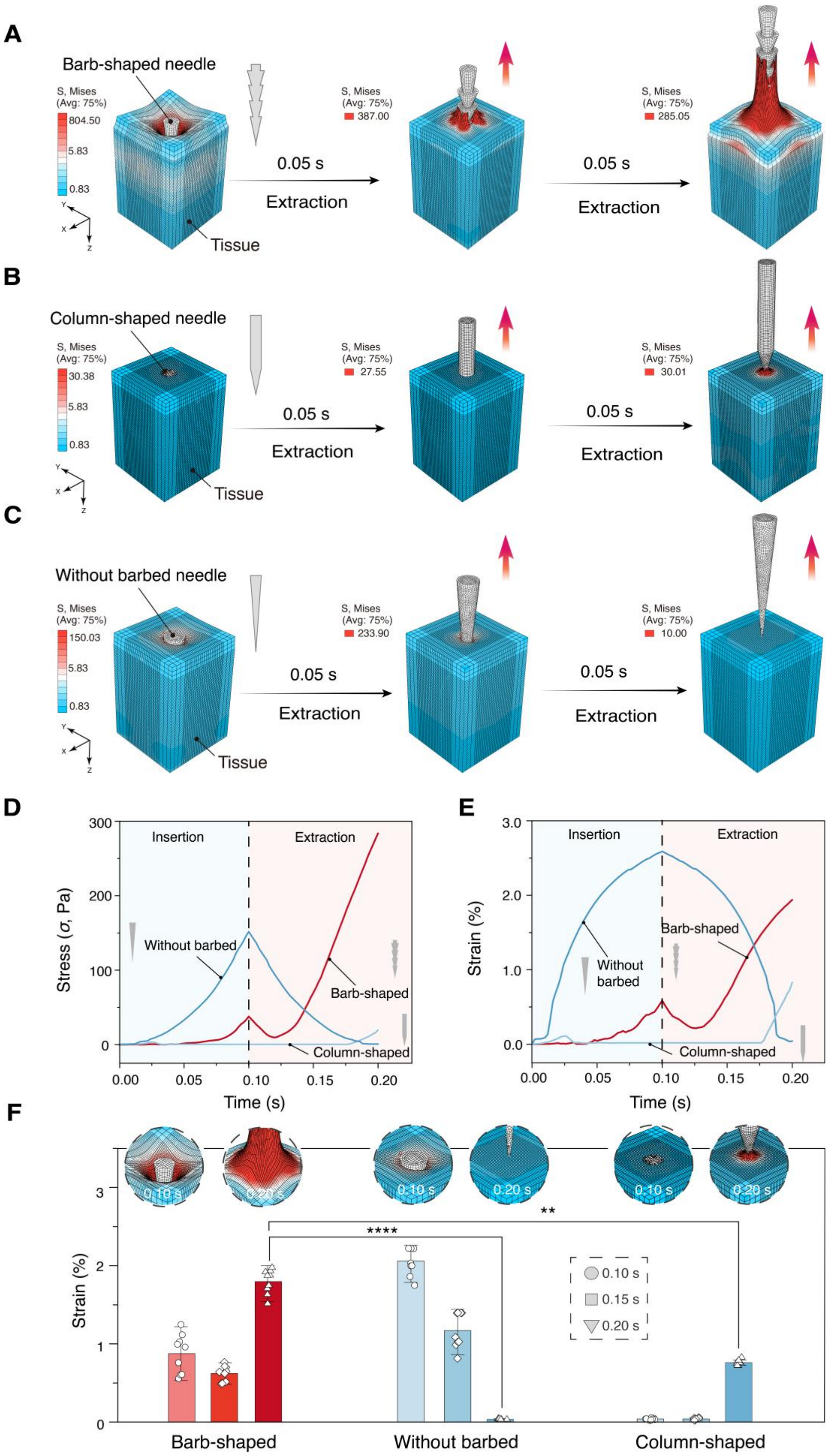

Fig. S13. Mechanical simulations of needles with different shapes (barb-shaped, without barbed and column-shaped) during insertion and extraction on gastric tissues. (A-C) Mechanical simulation results showed that barbless and columnar barb-

shaped needle has a slight hysteresis effect during tissue insertion and extraction, while barb-shaped needle has a significant retention effect. (D-E) Mechanical simulation of different shapes of needle insertion and extraction tissues with time-dependent stress and strain curves. (F) Summary of strains at critical times for different shaped needle extraction tissues, where the insets show the stress distribution of the tissues at the beginning and end of extraction. (n = 10).

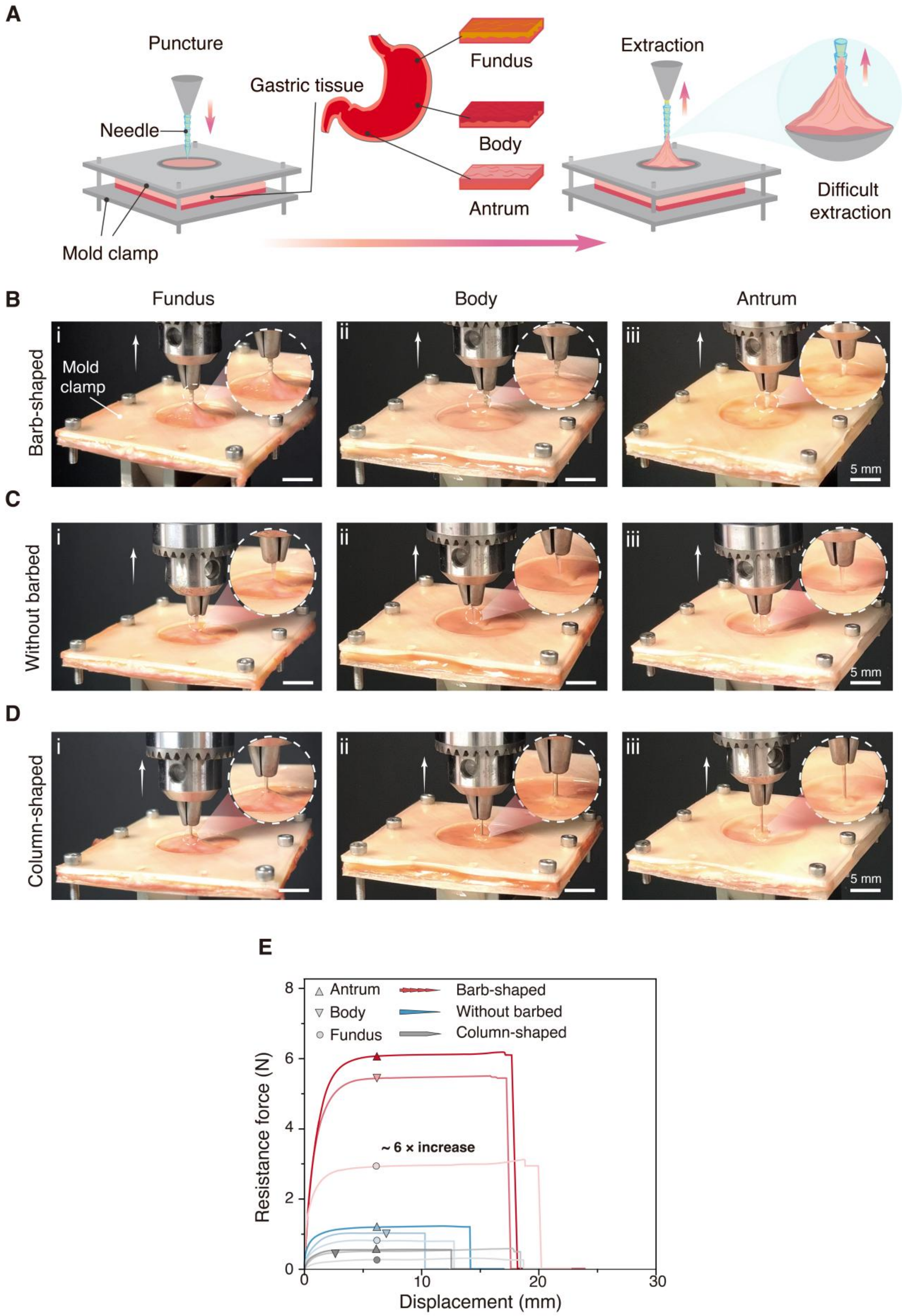

Fig. S14. Retention tests of barb-shaped needles in gastric tissue. (A-C) Schematic of barb-shaped needle insertion and extraction through gastric regions (fundus, body, antrum). (B-D) Comparison of barb-shaped, non-barbed, and column-shaped needle interactions across these regions. (E) Displacement-retention curves for different needle

shapes (barb-shaped, without barbed, and column-shaped) in different gastric tissue regions, showing that barb-shaped needles significantly increase resistance force (~6 ×) compared to control groups. Scale bar: 5 mm.

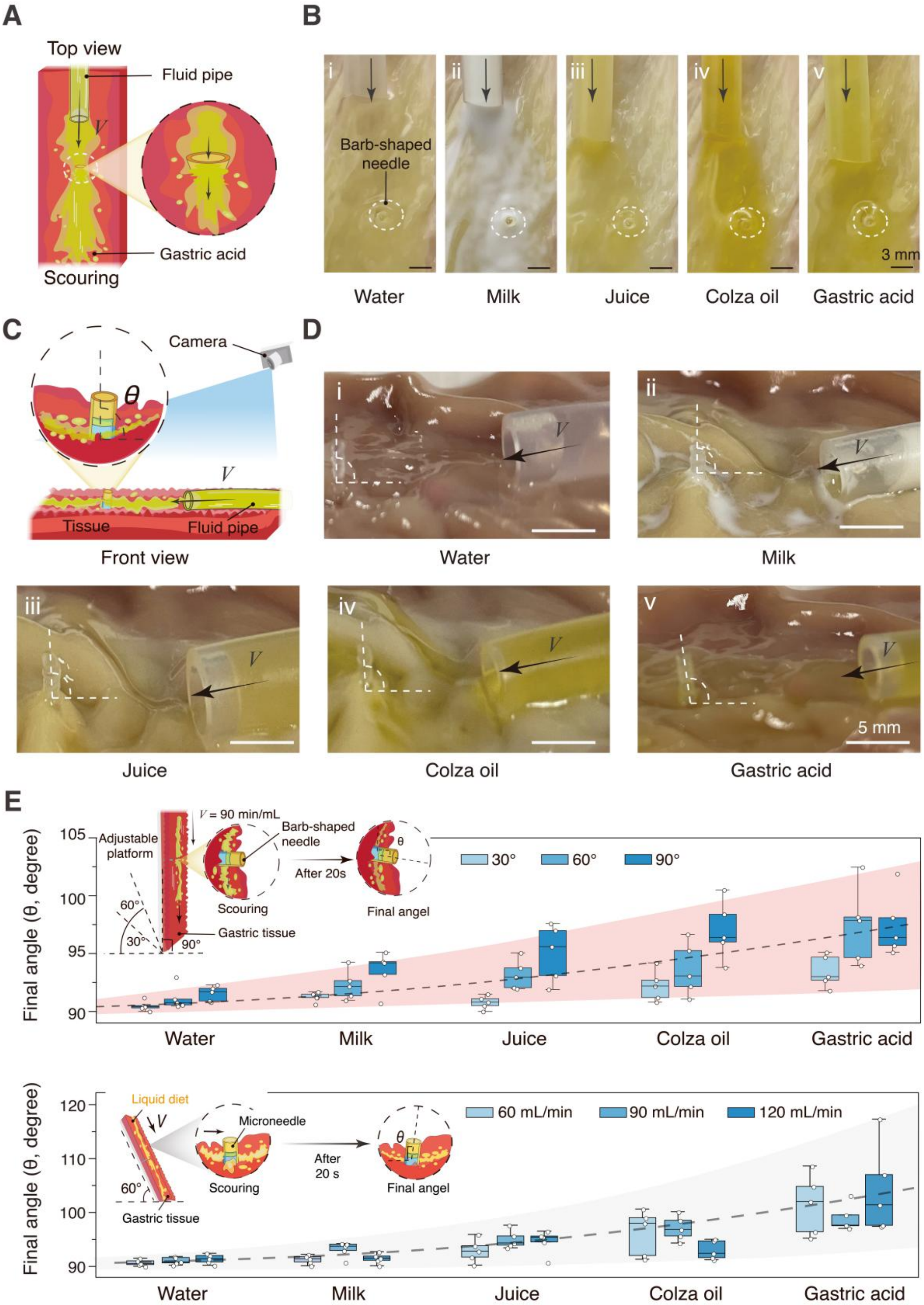

Fig. S15. Retention analysis of barb-shaped needles under scouring by different fluids. (A) Top view of the scouring experiment setup for the barb-shaped needle. (B) Scouring process of the barb-shaped needle using various test fluids, including water, milk, juice, colza oil, and gastric acid. (C) Overview of the complete experimental setup and procedure, with real-time recording of the scouring process. (D) Post-experimental state of the barb-shaped needle after exposure to each test fluid. (E) Final angle of the barb-shaped needle recorded after scouring at different initial angles (30°, 60°, and 90°)

with a constant flow rate for various common fluids, showing the effects of different fluids and angles on retention behavior. To assess the effect of the complex scouring environment in the stomach on the retention of a barb-shaped needle, different fluids and flow rates were used to assess needle movement while it was inserted vertically into *ex-vivo* tissue. (n = 5)

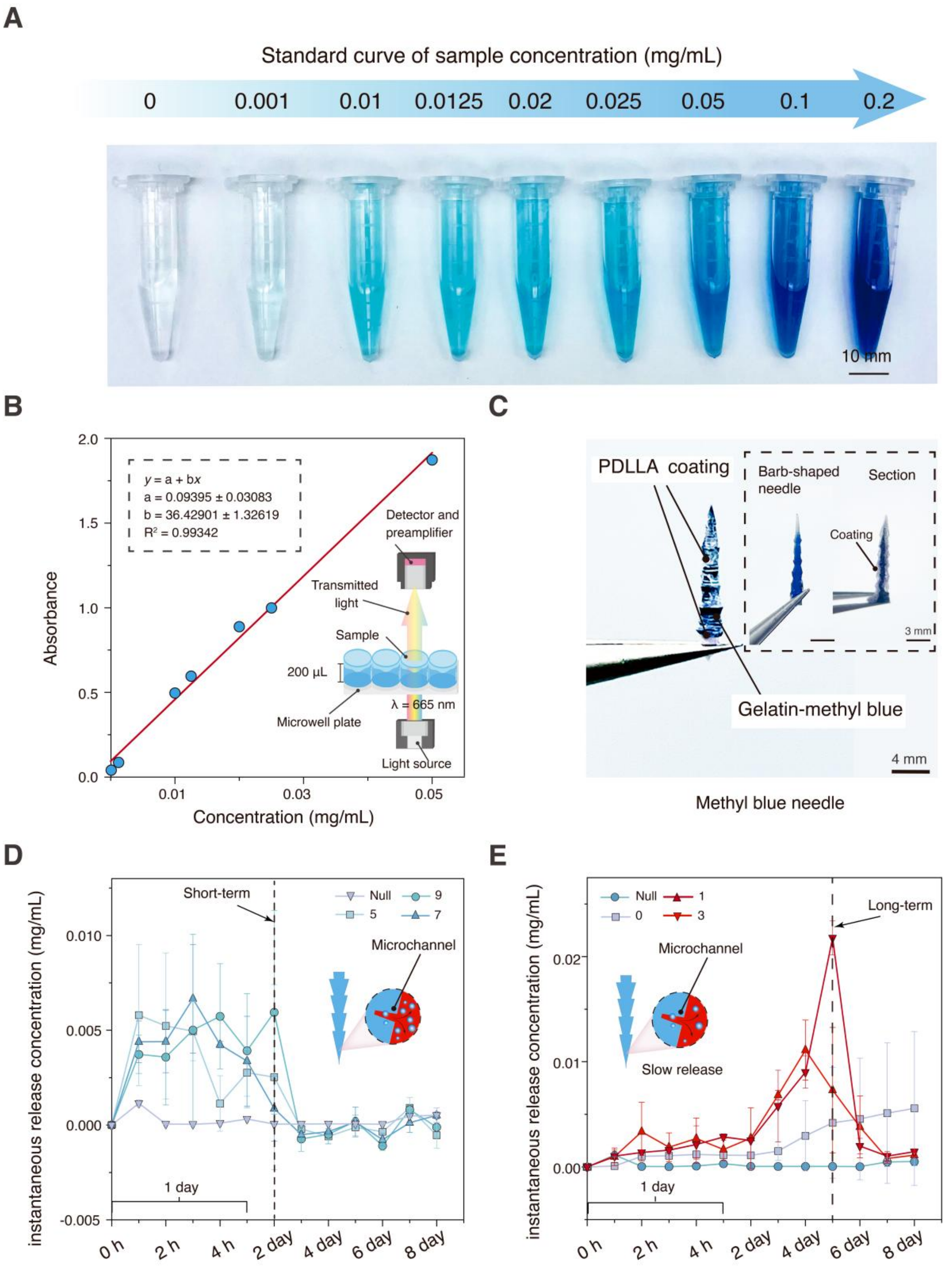

Fig. S16. ***Ex-vivo*** **drug release of barb-shaped needles.** (A) Preparation of gradient concentration methyl blue solutions for standard curve generation. (B) Calibration curve correlating methyl blue concentration with absorbance. (C) Barb-shaped needles modeled in methyl blue, uncoated needles, and coated section. (D-E) Comparative release kinetics of methyl blue from needles with different micro-channel counts; the effect instantaneously released barb-shaped needle of the micro-channel number 5, 7, 9 in short-term (D) and the barb-shaped needle with 0, 1, 3 micro-channel in long-term (E). (n = 3)

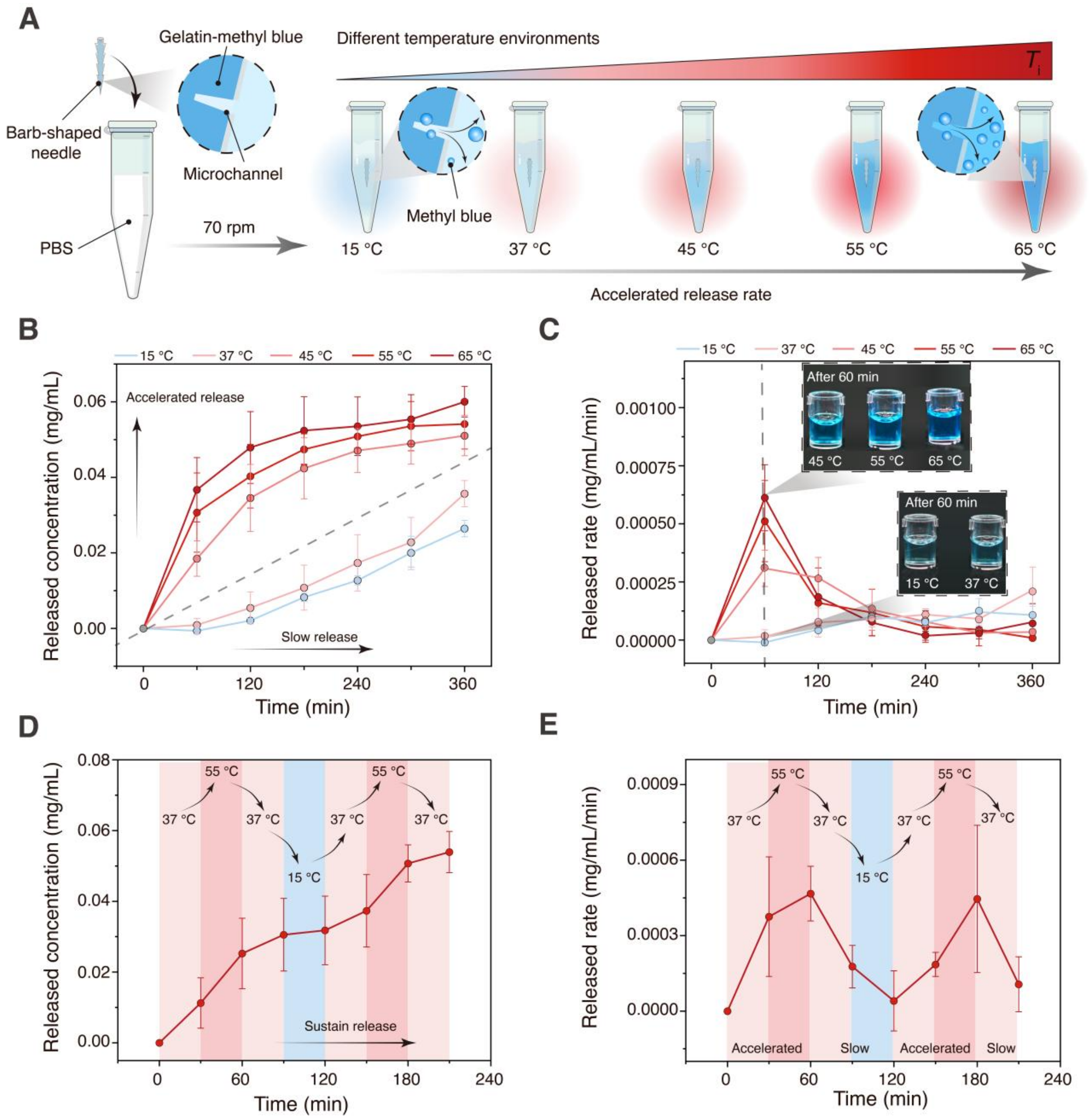

Fig. S17. Temperature-dependent release of gelatin-methyl blue in PBS solution under different temperature conditions. (A) Schematic illustration of the experiment, where the gelatin-methyl blue-loaded microchannel structures were exposed to different temperature environments (15 °C, 37 °C, 45 °C, 55 °C, and 65 °C) under 70 rpm shaking. (B-C) Cumulative released concentration and release rate of gelatin-methyl blue over time at different temperatures. (D-E) Cumulative released concentration and released rate under cyclic temperature changes, demonstrating alternating accelerated and slowly released phases. All data are presented as mean ± SD (n = 5).

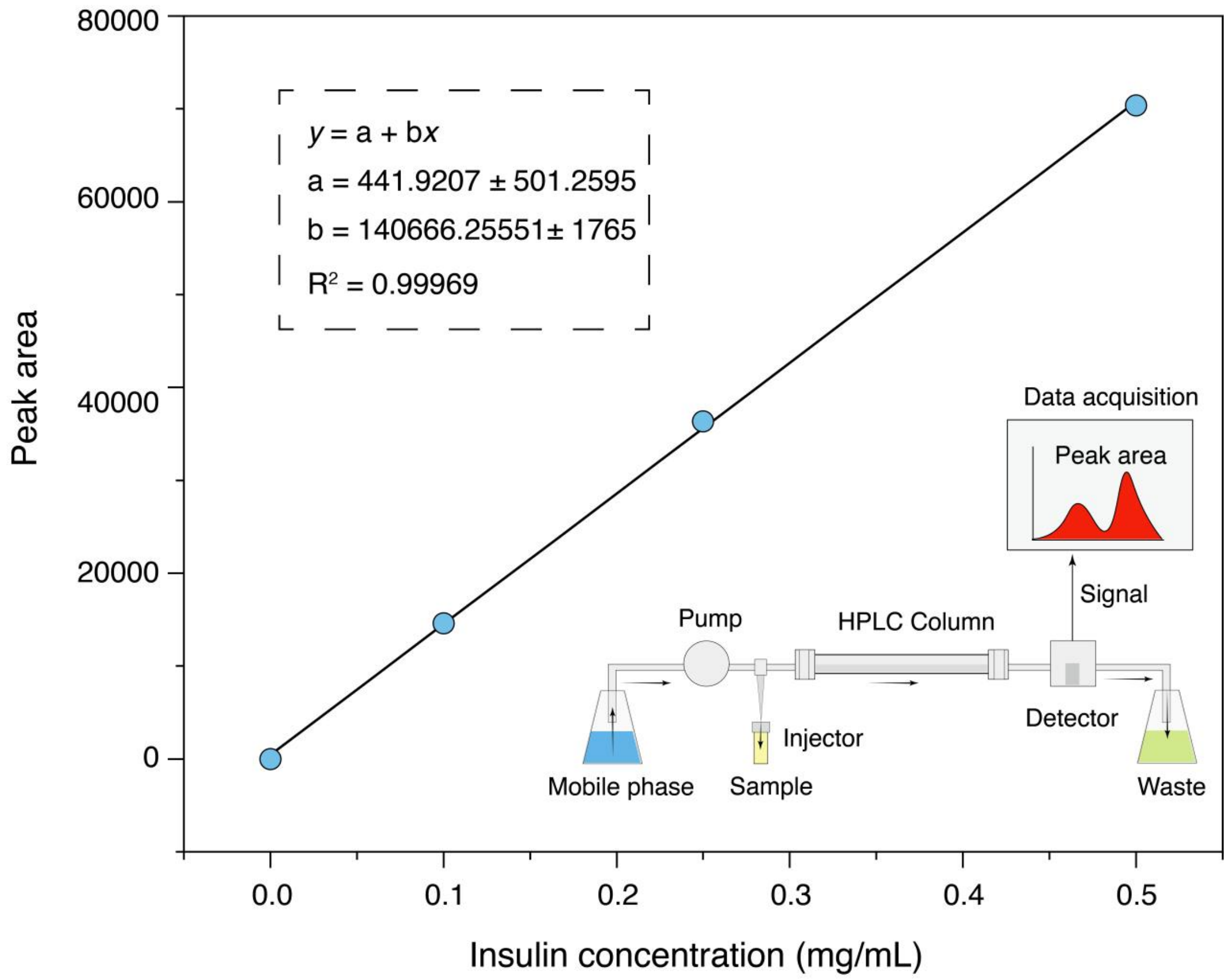

Fig. S18. HPLC analysis. The standard curve illustrates the peak areas corresponding to different concentrations of insulin solutions measured using High-Performance Liquid Chromatography (HPLC). The illustration depicts the fundamental principles of the HPLC testing process.

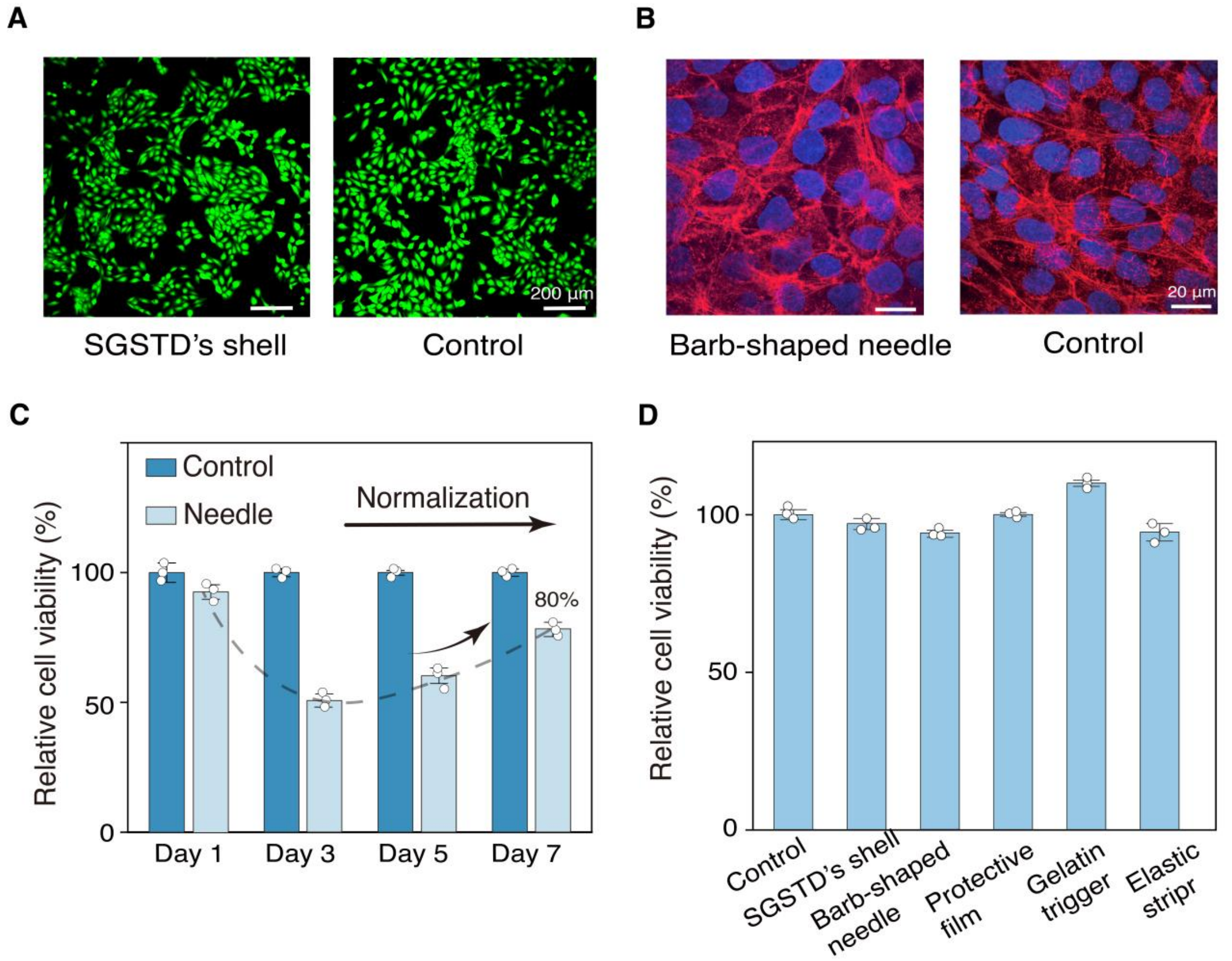

Fig. S19. Cell viability assessment of *GES-1* cells exposed to different SGSTD components and materials. (A) Live/dead staining of *GES-1* cells after incubation with SGSTD's shell for 7 days, showing high cell viability comparable to the control. Green fluorescence indicates live cells. Scale bar: 200 μm. (B) Fluorescence imaging of *GES-1* cells after exposure to the barb-shaped needle and control. Red fluorescence marks the cytoskeleton, and blue fluorescence marks the nucleus. Scale bar: 20 μm. (C) Relative cell viability over time (1, 3, 5, and 7 days) for *GES-1* cells incubated with barb-shaped needles compared to the control, showing gradual normalization and recovery to ~80% viability. (D) On the eighth day of contact with the SGSTD shell, serrated needles, protective film, gelatin trigger, and elastic strip, *GES-1* cells exhibited cell viability comparable to that of the control group, indicating acceptable cytocompatibility of the individual SGSTD components. (n = 3).

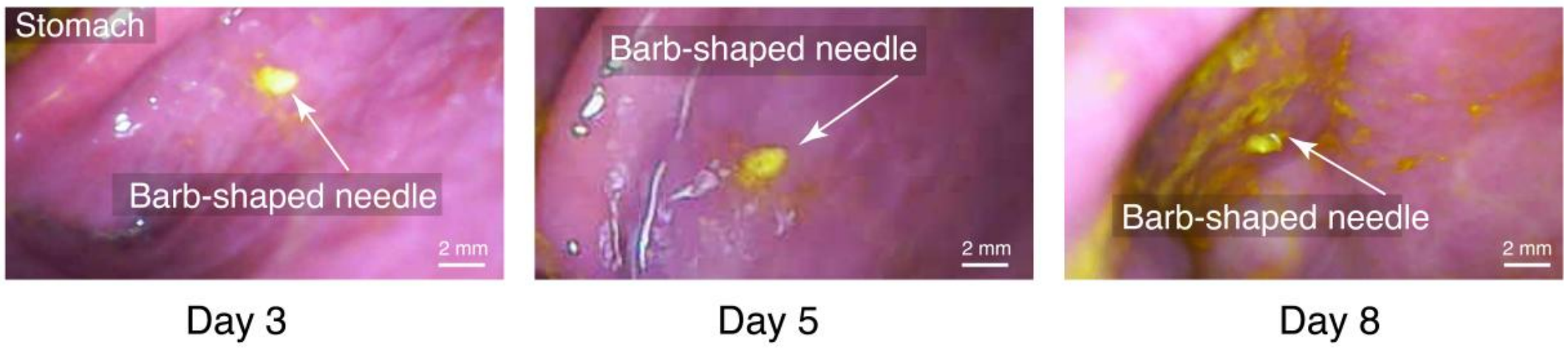

Fig. S20. Periodic endoscopic observation of gastric retention of the barb-shaped needle on Days 3, 5, and 8.

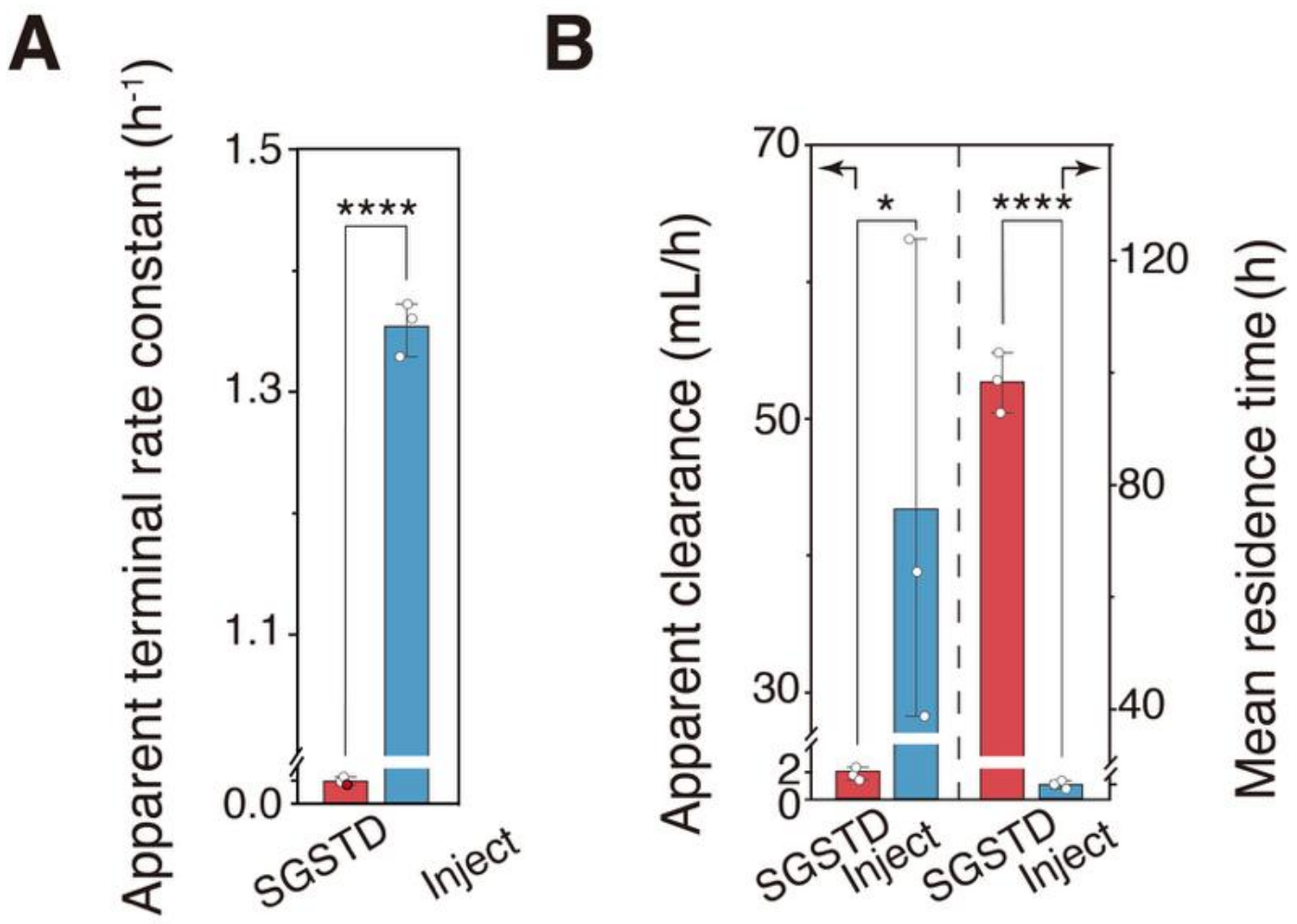

Fig. S21. Pharmacokinetic analysis of SGSTD and subcutaneous injection. (A) Apparent terminal rate constant of insulin concentration–time profiles after SGSTD administration and subcutaneous injection. (B) Apparent clearance and mean residence time calculated from the measured insulin concentration–time curves. Because both SGSTD and subcutaneous injection are non-intravenous administration routes, clearance is reported as apparent clearance. Data are presented as mean ± SD (n = 3).

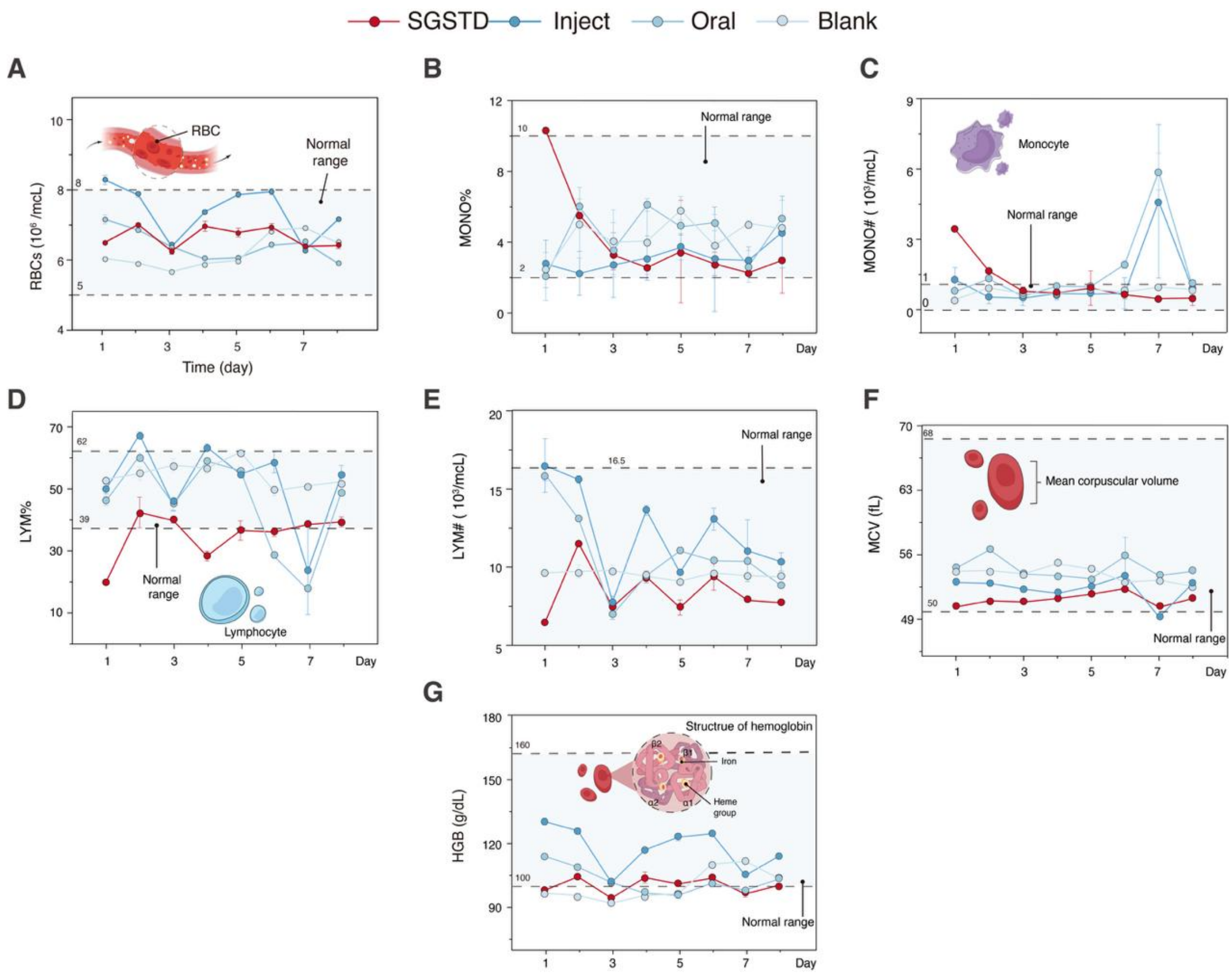

Fig. S22. Biochemical parameter dynamics in response to varied administration regimens. (A) The fluctuations in RBC (red blood cell count) over time indicate that the red blood cell count consistently remained within the normal range. The inset depicts red blood cells within the bloodstream. (B-C) The temporal changes in MONO% (monocyte percentage) and MONO# (monocyte count) demonstrate that, throughout the observation period, both metrics in the SGSTD group consistently remained within normal limits, showing no significant increases or fluctuations. (D-E) The variations in LYM% (lymphocyte percentage) and LYM# (lymphocyte count) over time indicate that both metrics stayed within the normal range and returned to baseline levels during the observation period, suggesting no significant health risks. (F) The trends in MCV (mean corpuscular volume) over time illustrate that the mean corpuscular volume remained stable throughout the observation period. (G) The variations in HGB (hemoglobin) over time show that hemoglobin levels consistently remained within the normal range, indicating no significant health risks. The inset illustrates the structure of hemoglobin. (n = 3)

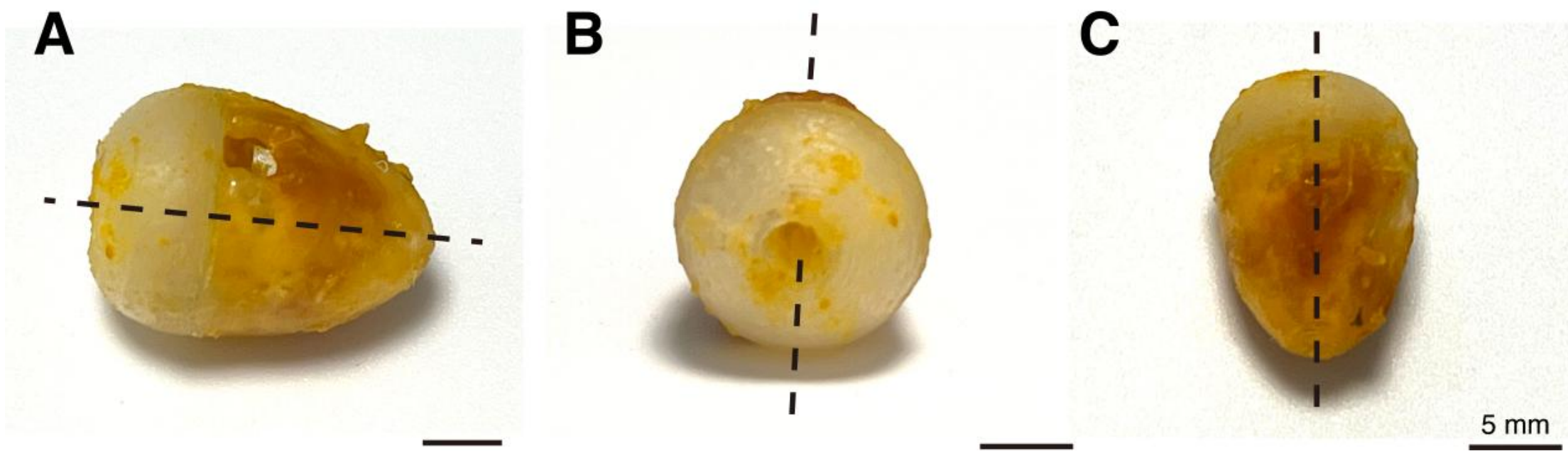

Fig. S23. SGSTD for internal excretion after 24 h post-operation. (A) Side view of SSGD. (B) Bottom of SGSTD after the needle with drug is detached. (C) Another side view of SGSTD.

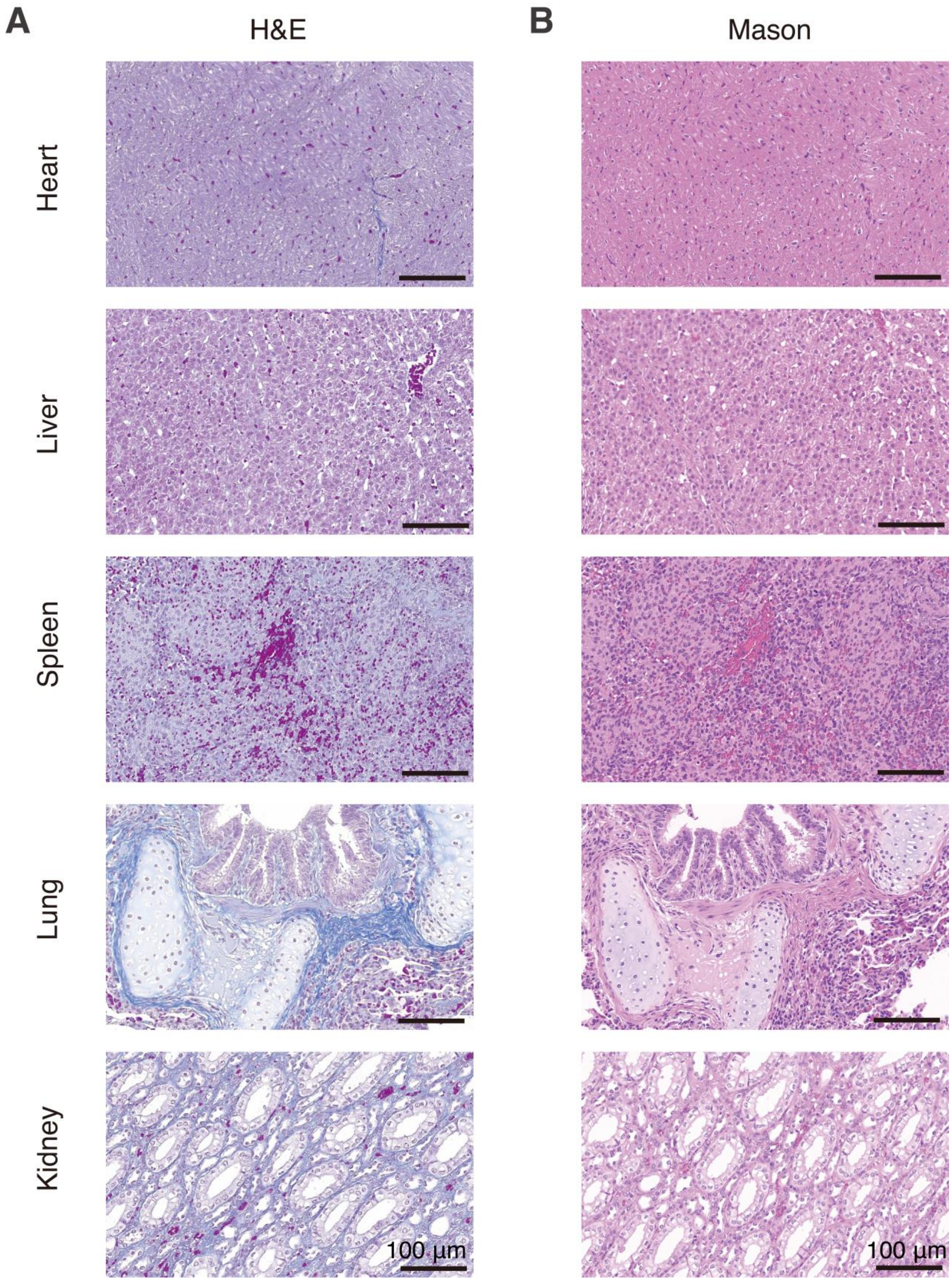

Fig. S24. Assessment of SGSTD's toxicity. H&E staining (A) and Mason staining (B) of principal organs (heart, liver, spleen, lung, kidney) of experimental pig model following an administration of device. Scale bar: 100 µm.

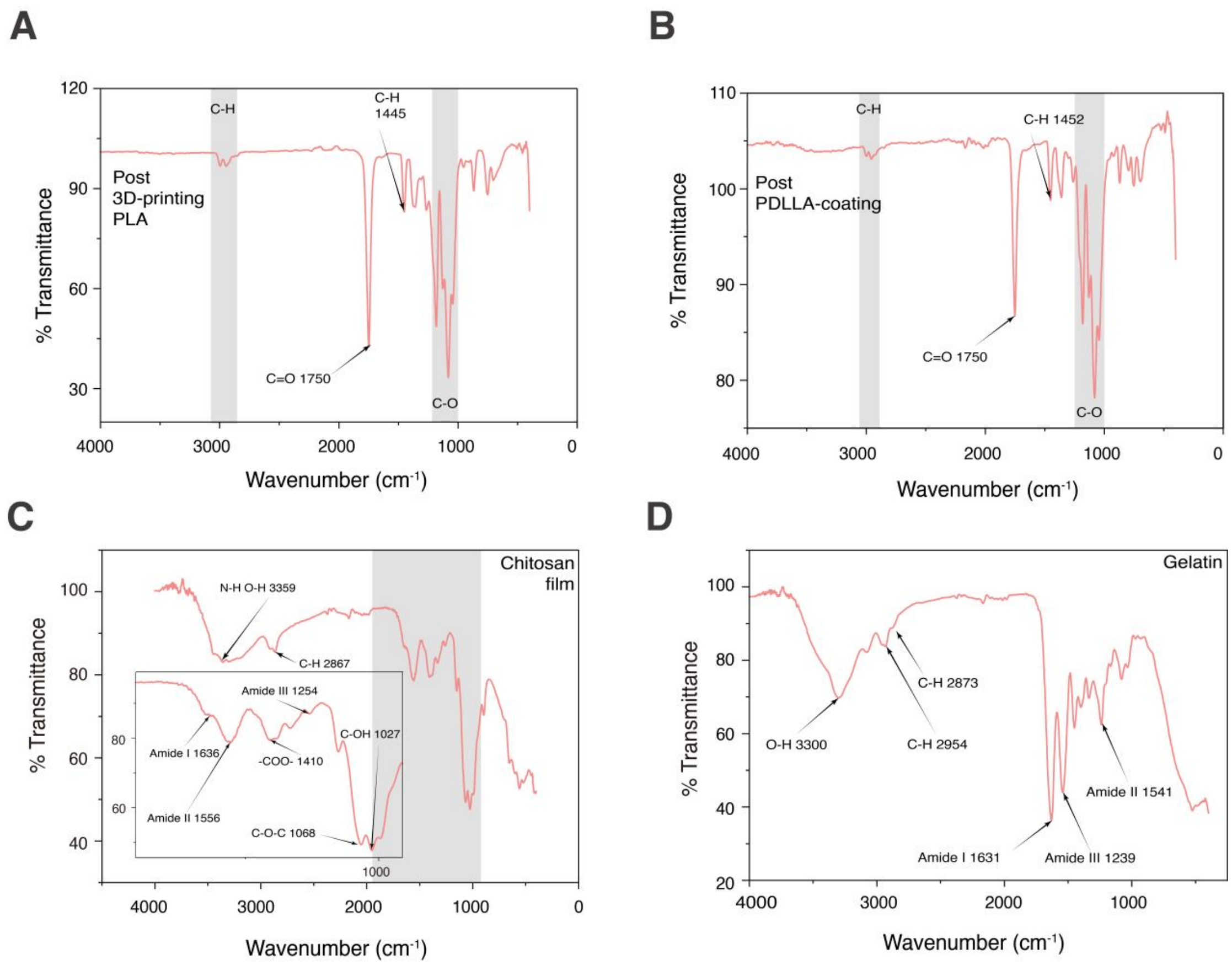

Fig. S25. Infrared spectroscopic analysis of SGSTD materials after processing. (A-B) The IR spectrum of the PLA shell and PDLLA coating surface after 3D-printing showed that the characteristic peaks of C=O stretching and significant C-H stretching vibration appeared at 1750 cm^{-1}, confirming the retention of the structural integrity of PDLLA after processing (*82*). (C) Post-processed chitosan film reveals an IR spectrum with characteristic peaks of chitosan, notably the N-H and O-H stretching vibrations at 3359 cm^{-1}, the amide bands (Amide I at 1636 cm^{-1} and Amide III at 1254 cm^{-1}), and the saccharide structure's C-O-C stretching vibration at 1068 cm^{-1} (*83*). (D) The processed gelatin barb-shaped needles showed an infrared spectrum typical of gelatin, including the broad O-H stretching near 3300 cm^{-1}, Amide I at 1631 cm^{-1}, Amide II at 1541 cm^{-1}, and a distinct Amide III band at 1239 cm^{-1}, reflecting the protein's composition (*84*).

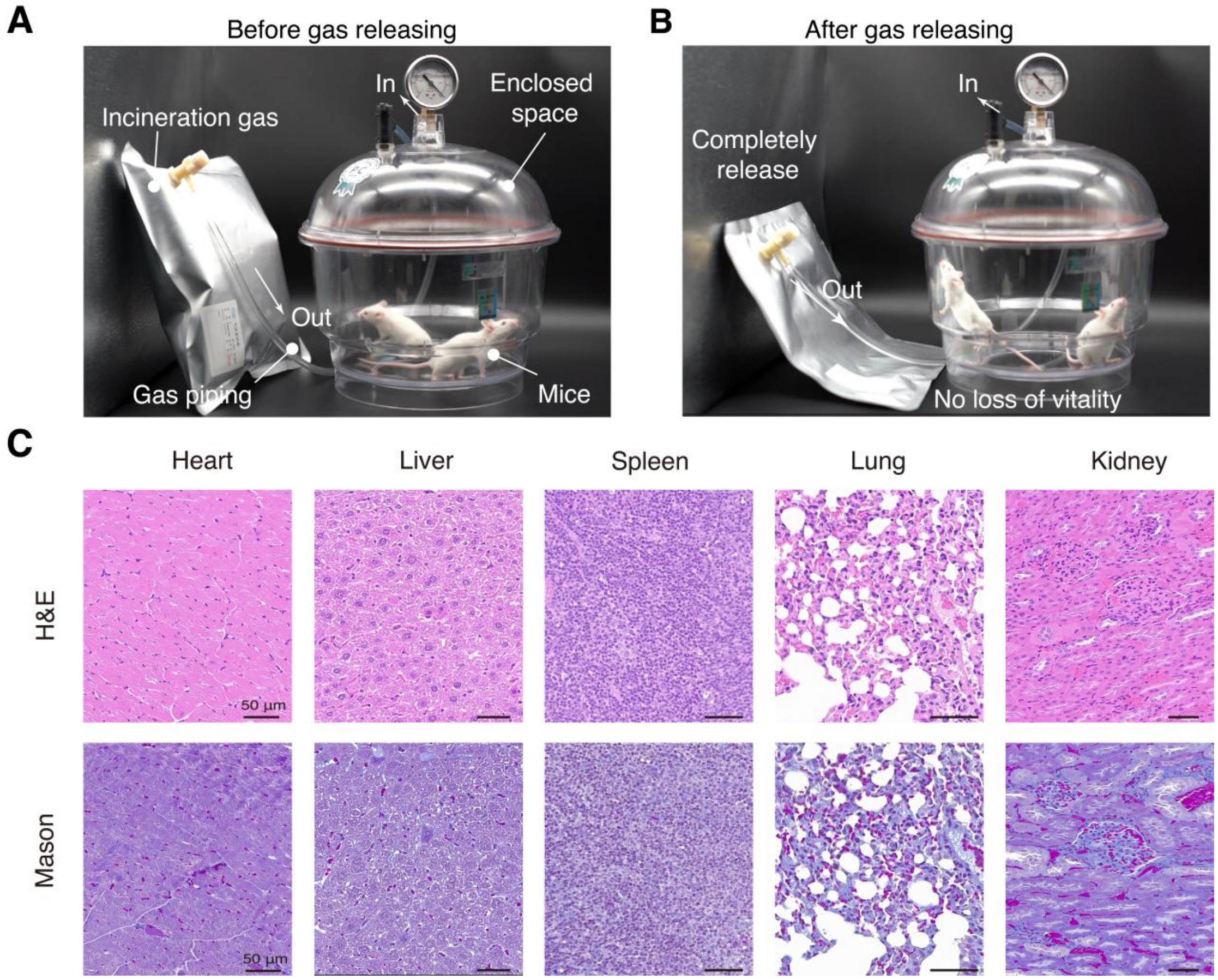

Fig.S26. Toxicity testing of gases from SGSTD incineration. (A-B) The experimental mice demonstrated excellent physiological tolerance during the incinerator exhaust gas inhalation test conducted in a closed environment. The mice did not exhibit any biological markers of toxicity following their intake of exhaust gases, and they remained normal both before and after the experiment, as indicated by behavioral toxicological assessment indicators (Table S7). (C) Mason staining and H&E staining of principal organs (heart, liver, spleen, lung, kidney) of experimental mice on thirty days after inhalation. Scale bar: 50 µm.

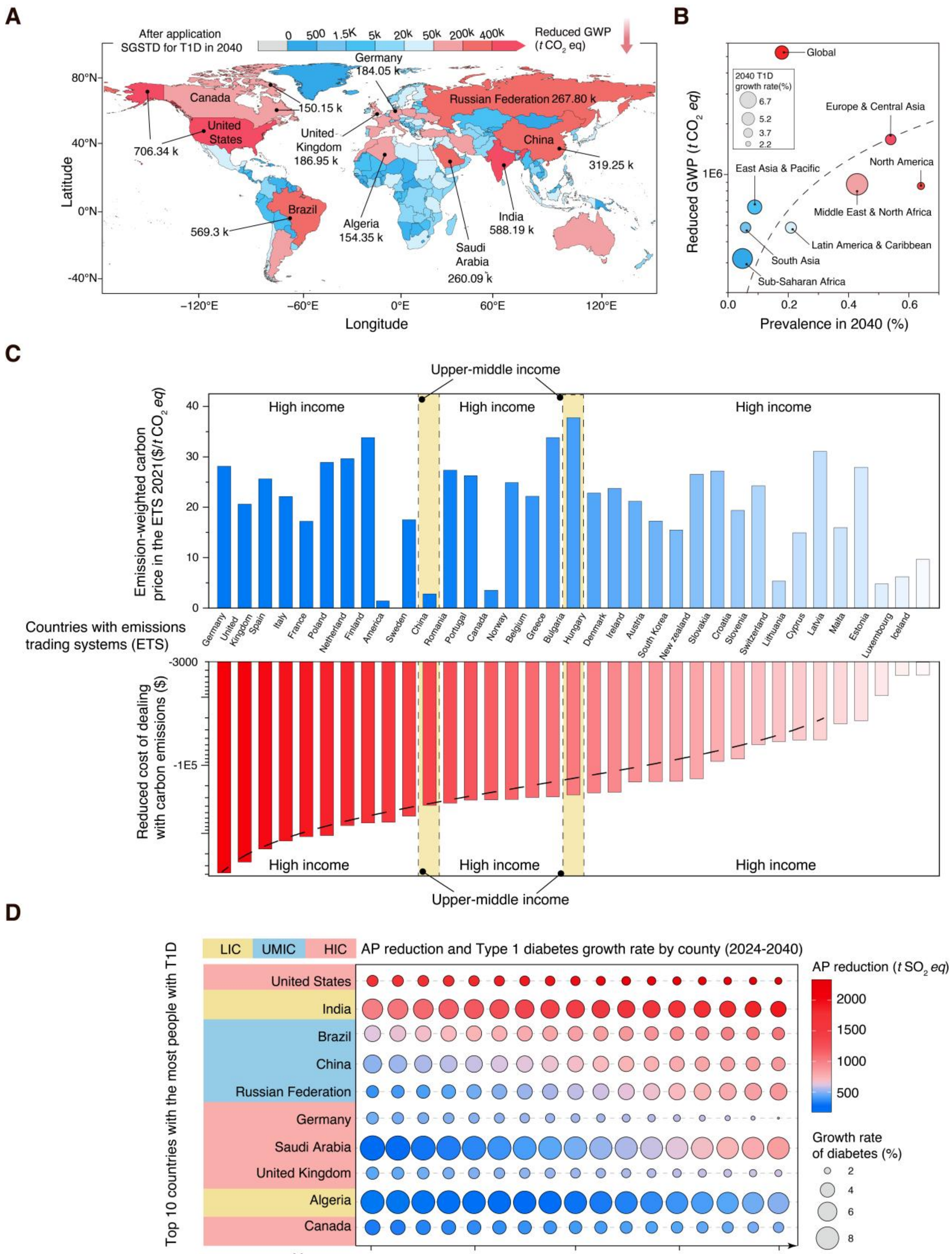

Fig. S27. Impact of SGSTD application in 2021 on countries with Emission Trading Systems (ETS). (A) The top panel shows the emission-weighted carbon price per ton of CO_2 *equivalent* for countries with ETS, while the bottom panel depicts the corresponding reduction in the cost of managing carbon emissions. (C)Countries are categorized by income level (high-income and upper-middle-income), highlighting the economic benefits of reduced emissions management

costs under an ETS framework. (D) **Projected acidification potential (AP) reduction and Type 1 diabetes (T1D) growth rate by country after SGSTD implementation (2024-2040).** The figure represents the top 10 countries with the highest number of individuals with T1D, categorized by income levels (LIC, UMIC, HIC). The color scale indicates the reduction in AP in tons of SO_2 *equivalent*, while the size of the circles represents the T1D growth rate (%).

Table S1. Comparative analysis of the key factors of SGSTD versus traditional diabetes treatment (injection and oral administration).

Performance metrics	SGSTD	Injection	Oral	Reference
**User-friendly**	Painless (Similar to taking *Omega* fish oil)	Painful	Painless	-
**Adherence**	～52 (Times/year)	～730-1460 (Times/year)	～1095 (Times/year)	*(52, 54)*
**Programmability**	Microchannel control, responsive material control, adjustable drug load	Non-programmable; injection only	Non-programmable; fixed dissolution kinetics	-
**Bioavailability**	～63.8%	～100%	< 1%	*(9, 10, 53)*
**Duration**	168 h	2-36 h	5-6 h	*(52)*
**Sustainability**	100% degradable	Non-biodegradable plastics (PE, PP, PC, *etc.*), metal needles, etc.	100% degradable	*(50, 51)*
**Applicability**	Macromolecular drugs, small molecule drugs, *etc*	Macromolecular drugs, small molecule drugs, *etc*	Small molecule drugs	*(7)*

Table S2. Fitting functions for different upper shell appearance device models.

**Sample**	**Fit function**
**Sharp**	$f(x) = 1.72 - (2.07) \times \mathrm{x}, f(x) = (2.07) \times \mathrm{x}$
**Low-slung**	$f(\mathrm{x}) = -0.04 + 11.19 \times \mathrm{x} - 83.18 \times \mathrm{x}^2 + 442.79 \times \mathrm{x}^3 - 1501.29 \times \mathrm{x}^4 + 3141.14 \times \mathrm{x}^5 - 3942.33 \times \mathrm{x}^6 + 2715.41 \times \mathrm{x}^7 - 788.53 \times \mathrm{x}^8$
**SGSTD**	$f(\mathrm{x}) = 0.03 + 7.00 \times \mathrm{x} - 38.97 \times \mathrm{x}^2 + 150.26 \times \mathrm{x}^3 - 320.59 \times x^4 + 332.70 \times x^5 - 133.59 \times x^6$
**Ellipsoidal**	$f(x) = 0.01 + 6.88 \times x - 40.11 \times x^2 + 147.23 \times x^3 - 299.24 \times x^4 + 301.72 \times x^5 - 119.23 \times x^6$
**Capsule**	$f(\mathrm{x}) = 0.63 + 3.23 \times \mathrm{x} - 18.72 \times \mathrm{x}^2 + 67.71 \times \mathrm{x}^3 - 137.04 \times \mathrm{x}^4 + 139.06 \times \mathrm{x}^5 - 55.73 \times \mathrm{x}^6$

Table S3. First-order derivatives for different upper shell appearance device models.

**Sample**	**First-order derivative**
**Sharp**	-
**Low-slung**	$f^{'}(x) = -6308.24 \times x^7 + 19007.87 \times x^6 - 23653.98 \times x^5 + 15705.7 \times x^4 - 6005.16 \times x^3 + 1328.37 \times x^2 - 166.36 \times x + 11.19$
**SGSTD**	$f^{'}(x) = -801.54 \times x^5 + 1663.5 \times x^4 - 1282.36 \times x^3 + 450.78 \times x^2 - 77.94 \times x + 7$
**Ellipsoidal**	$f^{'}(x) = -715.38 \times x^5 + 1508.6 \times x^4 - 1196.96 \times x^3 + 441.69 \times x^2 - 80.22 \times x + 6.88$
**Capsule**	$f^{'}(x) = -334.38 \times x^5 + 695.3 \times x^4 - 548.16 \times x^3 + 203.13 \times x^2 - 37.44 \times x + 3.23$

Table S4. Second-order derivatives for different upper shell appearance device models.

Sample	Second-order derivative
**Sharp**	-
**Low-slung**	$f''(x) = -44157.68 \times x^6 + 114047.22 \times x^5 - 118269.9 \times x^4 + 62822.8 \times x^3 - 18015.48 \times x^2 + 2656.74 \times x - 166.36$
**SGSTD**	$f''(x) = -4007.7 \times x^4 + 6654 \times x^3 - 3847.08 \times x^2 + 901.56 \times x - 77.94$
**Ellipsoidal**	$f''(x) = -3576.9 \times x^4 + 6034.4 \times x^3 - 3590.88 \times x^2 + 883.38 \times x - 80.22$
**Capsule**	$f''(x) = -1671.9 \times x^4 + 2781.2 \times x^3 - 1644.48 \times x^2 + 406.26 \times x - 37.44$

Table S5. Summarization of tangent points (X, Y), tangents ($Y = AX + B$), curvature (K), and radius of curvature (R) of the fitted functions for different upper shell appearance device models.

Sample	Tangent points (X, Y)	Tangents ($Y = AX + B$)	Curvature (K)	Radius of curvature (R)
**Sharp**	(0.40, 0.80)	$Y = 0.8$	-	-
**Low-slung**	(0.44, 0.99)	$Y = 0.99 - 0.02 \times X$	6.25	0.16
**SGSTD**	(0.43, 0.87)	$Y = 0.87 - 0.03 \times X$	10	0.1
**Ellipsoidal**	(0.42, 0.71)	$Y = 0.71 + 0.02 \times X$	7.14	0.14
**Capsule**	(0.42, 0.95)	$Y = 0.95 - 0.01 \times X$	2.70	0.37

Table S6. Summarization of volume (V), surface area (S), center of gravity (X,Y,Z), and principal moments of inertia (I_1, I_2, I_3) for devices of different shapes.

Sample	Volume (V, mm^3)	Surface area (S, mm^3)	Center of gravity (X, Y, Z, mm)	Principal moment of inertia (I_1, I_2, I_3, $g \times mm^3$)
**Sharp**	4.52	7.70	6.06, 5.77, 3.48	6.92, 6.92, 8.91
**Low-slung**	2.99	9.48	6.99, 6.05, 3.61	6.25, 6.49, 6.49

**SGSTD**	4.48	9.27	6.06, 6.08, 3.82	8.34, 8.34, 9.28
**Ellipsoidal**	5.97	8.72	6.10, 6.08, 5.17	1.03, 1.03, 1.20
**Capsule**	6.13	10.22	6.29, 6.08, 5.37	1.21, 1.21, 1.29

Table S7. Behavioral toxicity assessment of drug delivery devices.

Level	Behavioral Observation	Additional Notes
**Non-toxic**	Normal activity	No observable stress or discomfort
**Mild toxicity**	Temporary reduction in movement, slight tremors	Symptoms reversible, no long-term effect
**Moderate toxicity**	Dyspnea, decreased voluntary movement, eye Reduced activity, signs of respiratory distress	Non-lethal symptoms, but observable impact
**Severe toxicity**	Severe dyspnea, muscle Significant lethargy, lack of response to stimuli.	Significant weight loss, signs of dehydration
**Death**	Complete lack of movement, unresponsive	Incompatible with life-sustaining function

Table S8. Summary of drug delivery device.

**Project**	**Device**	**Way of taking**	**Disease model**	**API**	**Site of action**	**Drug duration**	**Biodegradability**	**Ref.**
**SGSTD**	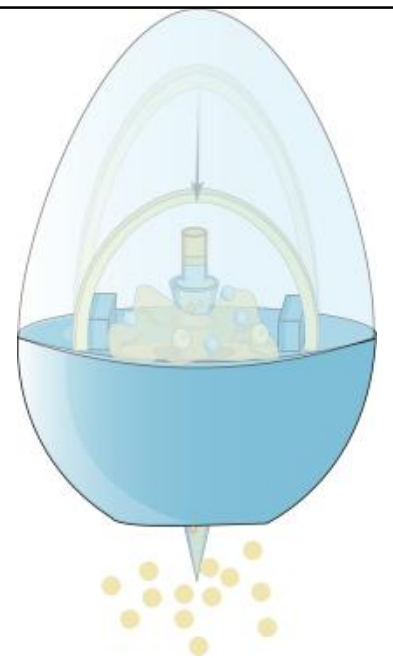	Ingest	Diabetes	Insulin	Stomach	192 h	Fully	-
**SOMA**	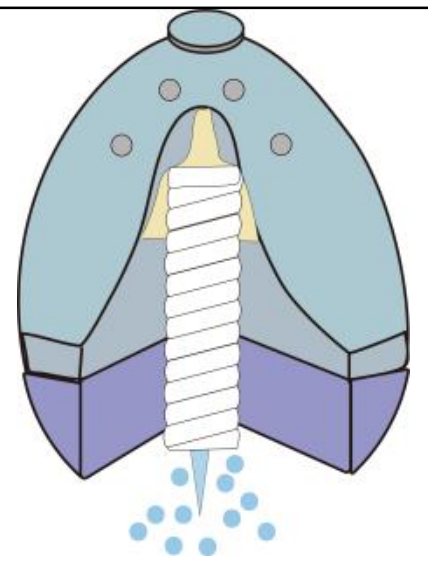	Ingest	Diabetes	Insulin	Stomach	30 h	Partially	*(5)*

**L-SOMA**	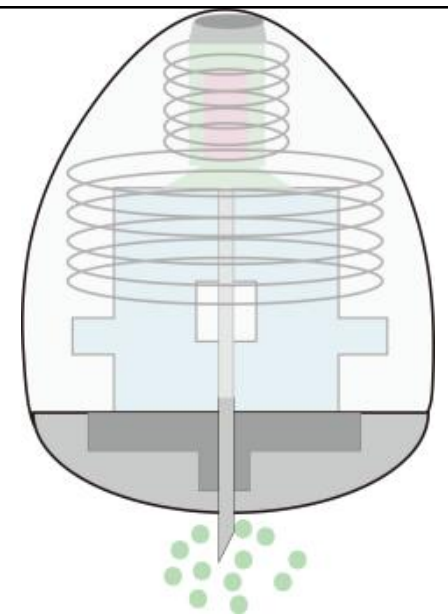	Ingest	Diabetes	Monoclonal antibodies (e.g. adalimumab), GLP-1 analogues, insulin and adrenaline	Stomach	2 h	Partially	*(56)*
**LUMI**	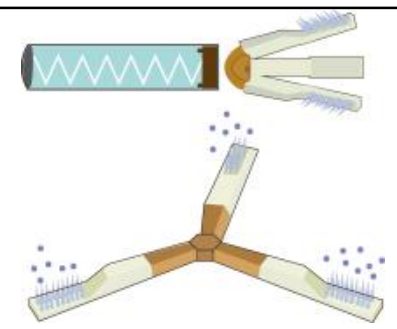	Ingest	Diabetes	Insulin	Enteric cavity	4 h	Partially	*(11)*
**DOAMS**	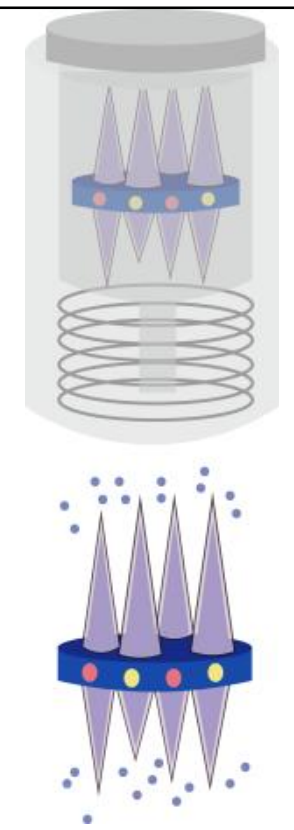	Ingest	Diabetes	Semaglutide	Stomach	72 h	Partially	*(12)*

**IPMR**	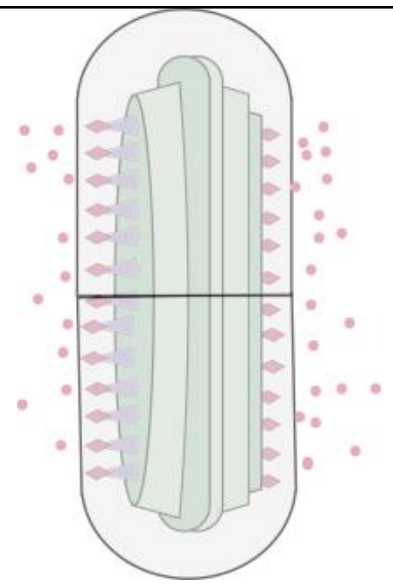	Ingest	Diabetes	Insulin	Enteric cavity	4 h	Fully	*(13)*
**MMR**	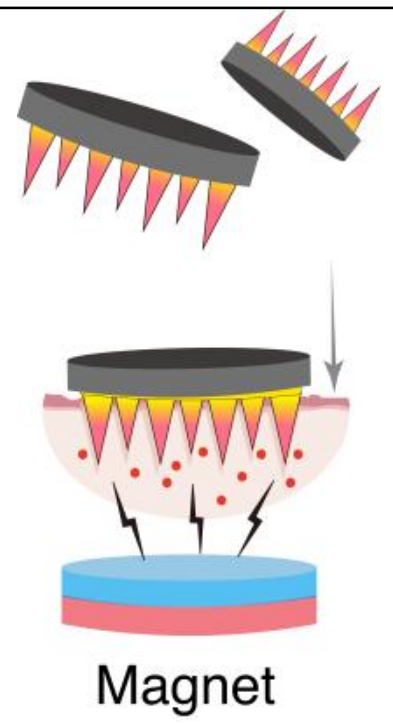	Ingest + applied magnetic field	Diabetes	Insulin	Enteric cavity	24 h	Partially	*(57)*
**RIEM**	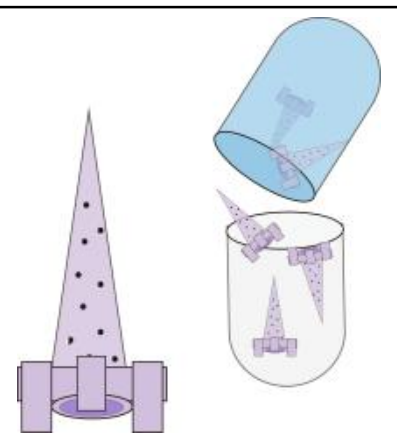	Ingest	Diabetes	Insulin	Enteric cavity	3.67 h	Fully	(*64*)
**RoboCap**	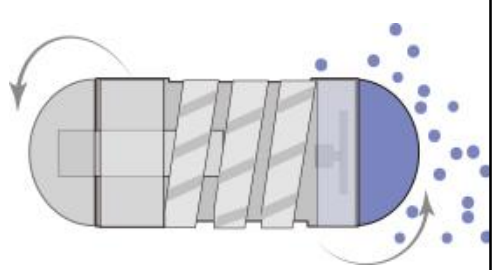	Ingest	Diabetes	Insulin and vancomycin	Enteric cavity	1 h	Non	*(58)*

**Theragrippers**	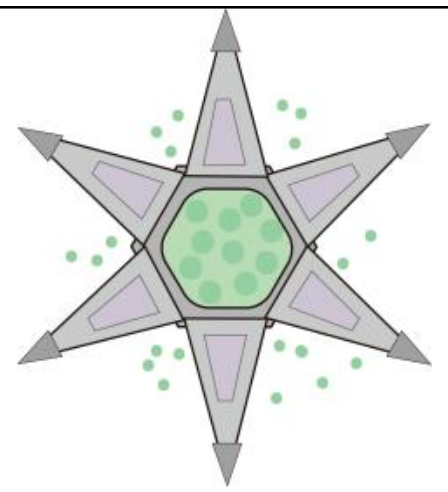	pneumatic delivery system (Fluigent)	Acute pain disorder	Ketorolac	Enteric cavity	24 h	Partially	*(59)*
**GSMD**	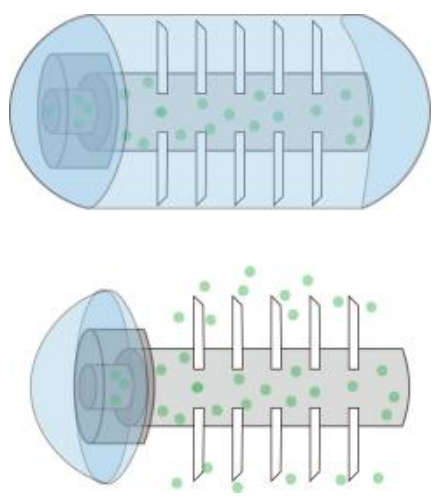	Ingest	Diabetes	Insulin	Enteric cavity	0.5 h	Partially	*(30)*
**LIFT**	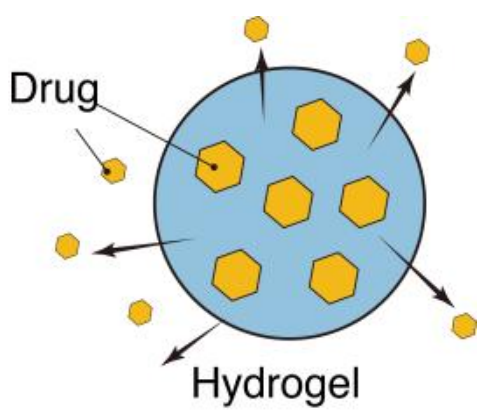	Ingest	Malarial	Lumefantrine	Stomach	48 h	Fully	(*65*)
**Algae-Np (Dox) motors**	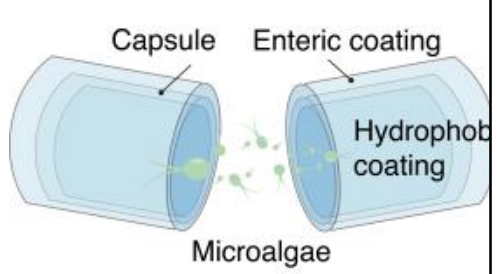	Ingest	Cancer	Doxorubicin	Stomach	18 h	Fully	(*66*)

**TIMCR**	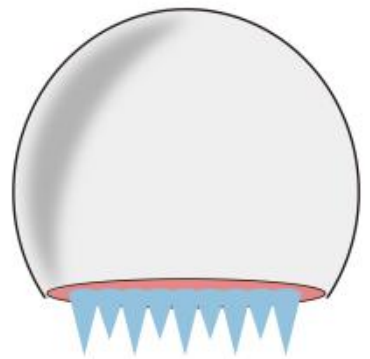	Ingest	-	Chitosan	Enteric cavity	48 h	Partially	*(55)*
**OODDS**	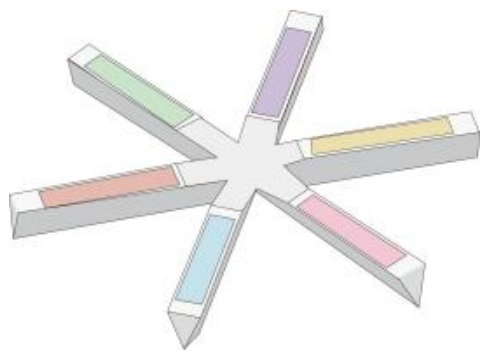	Ingest	HIV	Dolutegravir (DTG), cabotegravir (CAB), tenofovir alafenamide and rilpivirine (RPV)	Stomach	192 h	Partially	*(60)*
**IntelliCap^{R}**	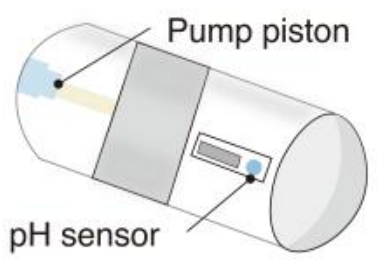	Ingest	Treat hypertension, angina pectoris, etc.	Diltiazem	Stomach	24 h	Partially	*(61)*

**AUM**	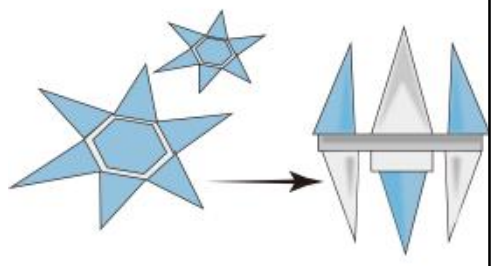	Ingest	Diabetes	Insulin	Enteric cavity	4 h	Partially	*(28)*
**ODP**	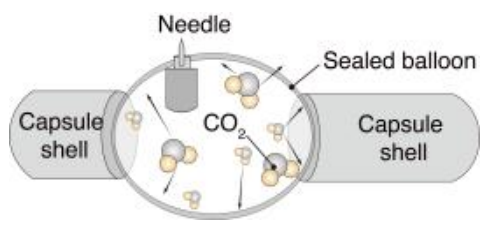	Ingest	Diabetes	Insulin	Enteric cavity	7.08 h	Partially	*(62)*
**RaniPill**	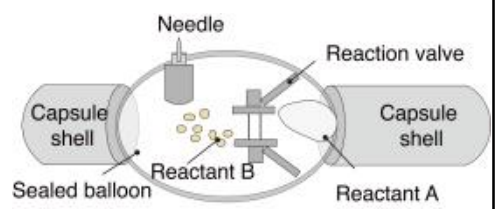	Ingest	Diabetes	Human parathyroid hormone analog, Insulin and Octreotide	Enteric cavity	4 h	Partially	(*67*)
**PDMS foil**	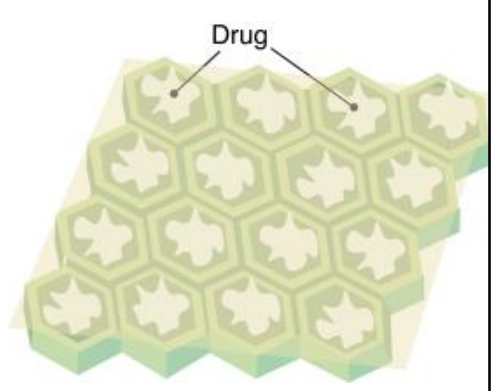	Ingest	Diabetes	Insulin	Enteric cavity	3 h	Partially	*(63)*

Other Supplementary Material for this manuscript includes:

Movie S1. The process of rapid customized fabrication of SGSTD. Using rapid 3D printing technology, the SGSTD's body and base have been completed.

Movie S2. The self-orienting behavior of SGSTD on the plane. By applying external pressure, the SGSTD is made to tip over, and upon the instant of pressure release, it rapidly self-orients on the plane. In addition, a free-fall test was carried out from a fixed height of 10 cm at different angles (30°, 90° and 180°) to observe the self-orientation effect of the device when dropped on the plane.

Movie S3. Assessment of the self-orientation phenomenon in-vitro using various shapes of free-falling devices. In comparison to the other shapes, SGSTD exhibits an excellent self-orientation effect in the in-vitro stomach model.

Movie S4. Simulate the self-orientation behavior of SGSTD as it rolls along tissue surfaces at different angles during the swallowing process, while recording the duration of its self-orientation.

Movie S5. The ability of devices of different shapes to orient themselves after experiencing a disturbance and quantifies the duration required to achieve this self-orientation. A disturbance was applied to the devices using a rotary shaker operating at a speed of 70 rpm for a duration of 20 s. After the shaking stopped, the angles of self-orientation were measured.

Movie S6. The effect of different shaped devices after free-fall onto the surface of stomach tissue with artificial gastric fluid. Different angles (30°, 90°, 180°) of release at the same fixed height of 15 cm were performed to observe the state of the device after dropping onto the surface of the isolated tissue.

Movie S7. The experiments that investigate the self-orientation reactions of devices with different shapes in a simulated intricate gastrointestinal setting. The devices were released from fixed positions at angles (30°, 60°, 90°), freely descending onto *ex-vivo* porcine gastric tissue. This tissue was coated with liquids of varying viscosities, including water, milk, juice, canola oil, and gastric fluid.

Movie S8. The process of energy accumulation using an elastic strap, followed by the ejection of a barb-shaped needle and its subsequent insertion into tissue. Finite element analysis (FEA) is used to simulate the stress distribution during the release and insertion stages.

Movie S9. The activation of SGSTD within the stomach over a fixed period of time, as observed via gastroscopy.

Movie S10. The insertion and subsequent extraction process of needles with different geometries (column-shaped, without barbed, and barb-shaped) in tissue. Finite element analysis simulates the stress distribution during both insertion and extraction phases.

Movie S11. The effect of different needles being extra from different parts of the pig stomach tissue. For three different areas of tissue, barb-shaped needle has a significant tissue retention effect.

Movie S12. The effect of simulating the retention of barb-shaped needles in the stomach under different conditions. Different flow rates with different viscosities of liquids were used to flush them to verify the long-lasting retention effect.

Movie S13. The effect of simulating the retention of a bark-shaped needle in the stomach under conditions of peristalsis and fluid erosion. The long-term retention effect was verified by rinsing with liquids of different viscosities and solid-liquid mixtures (simulating chyme).

Movie S14. The hydrodynamic simulation that illustrates the variations in drug concentration over time in a barb-shaped needle that carries the drug and has a barb-shaped construction.

Movie S15. The placement of experimental mice in a confined area and their exposure to gases obtained through burning. Following the gas emission, the mice exhibited no signs of adverse physiological reactions, remained relatively vigorous and could carry out normal activities.

REFERENCES

1. S. Mitragotri, P. A. Burke, R. Langer, Overcoming the challenges in administering biopharmaceuticals: Formulation and delivery strategies. *Nat. Rev. Drug Discov.* **13**, 655–672 (2014).

2. R. Langer, Drug delivery and targeting. *Nature* **392**, 5–10 (1998).

3. T. H. Baryakova, B. H. Pogostin, R. Langer, K. J. McHugh, Overcoming barriers to patient adherence: The case for developing innovative drug delivery systems. *Nat. Rev. Drug Discov.* **22**, 387–409 (2023).

4. World Health Organization, *Adherence to Long-Term Therapies: Evidence for Action* (World Health Organization, 2003); https://iris.who.int/handle/10665/42682.

5. J. N. Chu, G. Traverso, Foundations of gastrointestinal-based drug delivery and future developments. *Nat. Rev. Gastroenterol. Hepatol.* **19**, 219–238 (2022).

6. B. Homayun, X. Lin, H.-J. Choi, Challenges and recent progress in oral drug delivery systems for biopharmaceuticals. *Pharmaceutics* **11**, 129 (2019).

7. M. Goldberg, I. Gomez-Orellana, Challenges for the oral delivery of macromolecules. *Nat. Rev. Drug Discov.* **2**, 289–295 (2003).

8. N. G. Lamson, A. Berger, K. C. Fein, K. A. Whitehead, Anionic nanoparticles enable the oral delivery of proteins by enhancing intestinal permeability. *Nat. Biomed. Eng.* **4**, 84–96 (2020).

9. E. Mathiowitz, J. S. Jacob, Y. S. Jong, G. P. Carino, D. E. Chickering, P. Chaturvedi, C. A. Santos, K. Vijayaraghavan, S. Montgomery, M. Bassett, C. Morrell, Biologically erodable microspheres as potential oral drug delivery systems. *Nature* **386**, 410–414 (1997).

10. D. J. Brayden, M. J. Alonso, Oral delivery of peptides: Opportunities and issues for translation. *Adv. Drug Deliv. Rev.* **106**, 193–195 (2016).

11. A. Abramson, E. Caffarel-Salvador, V. Soares, D. Minahan, R. Y. Tian, X. Lu, D. Dellal, Y. Gao, S. Kim, J. Wainer, J. Collins, S. Tamang, A. Hayward, T. Yoshitake, H.-C. Lee, J.

Fujimoto, J. Fels, M. R. Frederiksen, U. Rahbek, N. Roxhed, R. Langer, G. Traverso, A luminal unfolding microneedle injector for oral delivery of macromolecules. *Nat. Med.* **25**, 1512–1518 (2019).

12. W. Chen, J. Wainer, S. W. Ryoo, X. Qi, R. Chang, J. Li, S. H. Lee, S. Min, A. Wentworth, J. E. Collins, S. Tamang, K. Ishida, A. Hayward, R. Langer, G. Traverso, Dynamic omnidirectional adhesive microneedle system for oral macromolecular drug delivery. *Sci. Adv.* **8**, eabk1792 (2022).

13. X. Gao, J. Li, J. Li, M. Zhang, J. Xu, Pain-free oral delivery of biologic drugs using intestinal peristalsis–actuated microneedle robots. *Sci. Adv.* **10**, eadj7067 (2024).

14. B. Ying, K. Nan, Q. Zhu, T. Khuu, H. Ro, S. Qin, S. Wang, K. Jiang, Y. Chen, G. Bao, J. Jenkins, A. Pettinari, J. Kuosmanen, K. Ishida, N. Fabian, A. Lopes, F. Codreanu, J. Morimoto, J. Li, A. Hayward, R. Langer, G. Traverso, An electroadhesive hydrogel interface prolongs porcine gastrointestinal mucosal theranostics. *Sci. Transl. Med.* **17**, eadq1975 (2025).

15. H. Huang, Y. Fan, Z. Chen, H. Hu, Y. Lyu, J. Xu, J. Xu, D. Wu, Y. Liang, Q. Mao, J. Wang, H. Zhang, H. Fu, Y. Guan, J. Chen, K. Xu, H. U. Chung, X. Yu, K. Nan, A gastric retentive robotic capsule enables emergency-prepared and responsive oral drug delivery in canine models. *Sci. Adv.* **11**, eady6745 (2025).

16. G. Traverso, R. Langer, Perspective: Special delivery for the gut. *Nature* **519**, S19 (2015).

17. Z. Kang, J. A. Gomez, A. M. Ross, A. R. Kirtane, M. Zhao, Y. Cai, F. X. Chen, C. L. Chen, I. D. Becdach, R. Dey, A. R. Ismael, I. Moon, Y. Yang, B. N. Muller, M. G. Say, A. Pettinari, J. Kobrin, J. Morimoto, T. Smierciak, A. Lopes, A. E. Erdogan, M. Murphy, N. Fabian, A. Guevara, B. Laidlaw, K. Schmidt, A. M. Hayward, A. H. Techet, C. P. Kenaley, G. Traverso, Mechanical underwater adhesive devices for soft substrates. *Nature* **643**, 1271–1280 (2025).

18. W. Zhang, W. Jiang, C. Zhang, X. Qin, H. Zheng, W. Xu, M. Cui, B. Wang, J. Wu, Z. Wang, Honeybee comb-inspired stiffness gradient-amplified catapult for solid particle repellency. *Nat. Nanotechnol.* **19**, 219–225 (2024).

19. K.-H. Jeong, J. Kim, L. P. Lee, Biologically inspired artificial compound eyes. *Science* **312**, 557–561 (2006).

20. S. H. Eraghi, A. Toofani, A. Khaheshi, M. Khorsandi, A. Darvizeh, S. Gorb, H. Rajabi, Wing coupling in bees and wasps: From the underlying science to bioinspired engineering. *Adv. Sci.* **8**, e2004383 (2021).

21. H. R. Hermann, Sting autotomy, a defensive mechanism in certain social hymenoptera. *Insectes Soc.* **18**, 111–120 (1971).

22. M. L. Winston, *The Biology of the Honey Bee* (Harvard Univ. Press, 1987).

23. P. K. Visscher, R. S. Vetter, S. Camazine, Removing bee stings. *Lancet* **348**, 301–302 (1996).

24. H. A. Dade, *Anatomy and Dissection of the Honeybee* (International Bee Research Association, 1977).

25. D. M. Bass, M. Prevo, D. S. Waxman, Gastrointestinal safety of an extended-release, nondeformable, oral dosage form (OROS®). *Drug Saf.* **25**, 1021–1033 (2002).

26. U.S. Food and Drug Administration, Medical Device Reporting (MDR): How to Report Medical Device Problems. FDA (2025); https://fda.gov/medical-devices/medical-device-safety/medical-device-reporting-mdr-how-report-medical-device-problems.

27. A. Abramson, E. Caffarel-Salvador, M. Khang, D. Dellal, D. Silverstein, Y. Gao, M. R. Frederiksen, A. Vegge, F. Hubálek, J. J. Water, A. V. Friderichsen, J. Fels, R. K. Kirk, C. Cleveland, J. Collins, S. Tamang, A. Hayward, T. Landh, S. T. Buckley, N. Roxhed, U. Rahbek, R. Langer, G. Traverso, An ingestible self-orienting system for oral delivery of macromolecules. *Science* **363**, 611–615 (2019).

28. R. Mundaca-Uribe, N. Askarinam, R. H. Fang, L. Zhang, J. Wang, Towards multifunctional robotic pills. *Nat. Biomed. Eng.* **8**, 1334–1346 (2024).

29. U.S. Food and Drug Administration, Size, Shape, and Other Physical Attributes of Generic Tablets and Capsules: Guidance for Industry (Final Level 2 Revised Guidance, Center for

Drug Evaluation and Research, FDA, 2022); https://fda.gov/regulatory-information/search-fda-guidance-documents/size-shape-and-other-physical-attributes-generic-tablets-and-capsules.

30. G. Traverso, C. M. Schoellhammer, A. Schroeder, R. Maa, G. Y. Lauwers, B. E. Polat, D. G. Anderson, D. Blankschtein, R. Langer, Microneedles for drug delivery via the gastrointestinal tract. *J. Pharm. Sci.* **104**, 362–367 (2015).

31. J. Wallace, D. Granger, The cellular and molecular basis of gastric mucosa defense. *FASEB J.* **10**, 731–740 (1996).

32. E. Kaffash, M.-A. Shahbazi, H. Hatami, A. Nokhodchi, An insight into gastrointestinal macromolecule delivery using physical oral devices. *Drug Discov. Today* **27**, 2309–2321 (2022).

33. S. Farah, D. G. Anderson, R. Langer, Physical and mechanical properties of PLA, and their functions in widespread applications—A comprehensive review. *Adv. Drug Deliv. Rev.* **107**, 367–392 (2016).

34. D. I. Soybel, Anatomy and physiology of the stomach. *Surg. Clin. North Am.* **85**, 875–894 (2005).

35. F. Brown, K. R. Diller, Calculating the optimum temperature for serving hot beverages. *Burns* **34**, 648–654 (2008).

36. H.-S. Lee, M. O'Mahony, At what temperatures do consumers like to drink coffee?: Mixing methods. *J. Food Sci.* **67**, 2774–2777 (2006).

37. S. Babaee, S. Pajovic, A. R. Kirtane, J. Shi, E. Caffarel-Salvador, K. Hess, J. E. Collins, S. Tamang, A. V. Wahane, A. M. Hayward, H. Mazdiyasni, R. Langer, G. Traverso, Temperature-responsive biometamaterials for gastrointestinal applications. *Sci. Transl. Med.* **11**, eaau8581 (2019).

38. P. Li, V. Kothari, B. S. Terry, Design and preliminary experimental investigation of a capsule for measuring the small intestine contraction pressure. *IEEE Trans. Biomed. Eng.* **62**, 2702–2708 (2015).

39. W. Heidemann, S. Jeschkeit, K. Ruffieux, J. H. Fischer, M. Wagner, G. Krüger, E. Wintermantel, K. L. Gerlach, Degradation of poly(D,L)lactide implants with or without addition of calciumphosphates in vivo. *Biomaterials* **22**, 2371–2381 (2001).

40. L. Wang, Q. Liang, Q. Chen, J. Xu, Z. Shi, Z. Wang, Y. Liu, H. Ma, Hydrolysis kinetics and radical-scavenging activity of gelatin under simulated gastrointestinal digestion. *Food Chem.* **163**, 1–5 (2014).

41. National Oceanic and Atmospheric Administration, The Atmosphere. JetStream (2024); https://noaa.gov/jetstream/atmosphere.

42. World Health Organization, *WHO Global Air Quality Guidelines: Particulate Matter (PM2. 5 and PM10), Ozone, Nitrogen Dioxide, Sulfur Dioxide and Carbon Monoxide* (World Health Organization, 2021).

43. J. Hong, S. Zhan, Z. Yu, J. Hong, C. Qi, Life-cycle environmental and economic assessment of medical waste treatment. *J. Clean. Prod.* **174**, 65–73 (2018).

44. I. Linkov, B. D. Trump, B. A. Wender, T. P. Seager, A. J. Kennedy, J. M. Keisler, Integrate life-cycle assessment and risk analysis results, not methods. *Nat. Nanotechnol.* **12**, 740–743 (2017).

45. V. Lopes, A. D. Sousa Lages, Waste associated to diabetes management: The elephant in the room of technology? *Diabetes Res. Clin. Pract.* **185**, 109780 (2022).

46. H. Frumkin, Health care waste and climate change. *Am. J. Public Health* **111**, e15 (2021).

47. International Diabetes Federation, *IDF Diabetes Atlas, 11th ed*. (International Diabetes Federation, 2025); https://diabetesatlas.org.

48. R. P. Feynman, There’s plenty of room at the bottom. *Resonance* **16**, 890–905 (2011).

49. L. van Meer, M. Moerland, J. Gallagher, M. B. van Doorn, E. P. Prens, A. F. Cohen, R. Rissmann, J. Burggraaf, Injection site reactions after subcutaneous oligonucleotide therapy. *Br. J. Clin. Pharmacol.* **82**, 340–351 (2016).

50. B. Quronfuleh, D. Sleath, S. Rahimifard, Circular economy for medical devices: A case study of syringes. *Procedia CIRP* **122**, 449–454 (2024).

51. N. S. de Souza Lima Cano, M. Marino, A. L. Brooks, M. J. Eckelman, M. M. Bilec, Advancing the circular economy of healthcare plastics: A systematic literature review. *Resour. Conserv. Recycl.* **219**, 108317 (2025).

52. R. Eldor, E. Arbit, A. Corcos, M. Kidron, Glucose-reducing effect of the ORMD-0801 oral insulin preparation in patients with uncontrolled type 1 diabetes: A pilot study. *PLOS ONE* **8**, e59524 (2013).

53. J. P. Griffin, *The Textbook of Pharmaceutical Medicine: 6th Edition* (Wiley-Blackwell Press, 2009).

54. American Diabetes Association, Insulin Routines. Diabetes.org; https://diabetes.org/health-wellness/medication/insulin-routines.

55. L. Huang, L. Li, Y. Jiang, J. Cai, S. Cai, Y. Ren, H. Xu, J. Luo, M. Wang, L. Ren, Tumbler-inspired microneedle containing robots: Achieving rapid self-orientation and peristalsis-resistant adhesion for colonic administration. *Adv. Funct. Mater.* **33**, 2304276 (2023).

56. A. Abramson, M. R. Frederiksen, A. Vegge, B. Jensen, M. Poulsen, B. Mouridsen, M. O. Jespersen, R. K. Kirk, J. Windum, F. Hubálek, J. J. Water, J. Fels, S. B. Gunnarsson, A. Bohr, E. M. Straarup, M. W. H. Ley, X. Lu, J. Wainer, J. Collins, S. Tamang, K. Ishida, A. Hayward, P. Herskind, S. T. Buckley, N. Roxhed, R. Langer, U. Rahbek, G. Traverso, Oral delivery of systemic monoclonal antibodies, peptides and small molecules using gastric auto-injectors. *Nat. Biotechnol.* **40**, 103–109 (2022).

57. X. Zhang, G. Chen, X. Fu, Y. Wang, Y. Zhao, Magneto-responsive microneedle robots for intestinal macromolecule delivery. *Adv. Mater.* **33**, e2104932 (2021).

58. S. S. Srinivasan, A. Alshareef, A. V. Hwang, Z. Kang, J. Kuosmanen, K. Ishida, J. Jenkins, S. Liu, W. A. M. Madani, J. Lennerz, A. Hayward, J. Morimoto, N. Fitzgerald, R. Langer, G. Traverso, RoboCap: Robotic mucus-clearing capsule for enhanced drug delivery in the gastrointestinal tract. *Sci. Robot.* **7**, eabp9066 (2022).

59. A. Ghosh, L. Li, L. Xu, R. P. Dash, N. Gupta, J. Lam, Q. Jin, V. Akshintala, G. Pahapale, W. Liu, A. Sarkar, R. Rais, D. H. Gracias, F. M. Selaru, Gastrointestinal-resident, shape-changing microdevices extend drug release in vivo. *Sci. Adv.* **6**, eabb4133 (2020).

60. A. R. Kirtane, O. Abouzid, D. Minahan, T. Bensel, A. L. Hill, C. Selinger, A. Bershteyn, M. Craig, S. S. Mo, H. Mazdiyasni, C. Cleveland, J. Rogner, Y.-A. L. Lee, L. Booth, F. Javid, S. J. Wu, T. Grant, A. M. Bellinger, B. Nikolic, A. Hayward, L. Wood, P. A. Eckhoff, M. A. Nowak, R. Langer, G. Traverso, Development of an oral once-weekly drug delivery system for HIV antiretroviral therapy. *Nat. Commun.* **9**, 2 (2018).

61. D. Becker, J. Zhang, T. Heimbach, R. C. Penland, C. Wanke, J. Shimizu, K. Kulmatycki, Novel orally swallowable IntelliCap® device to quantify regional drug absorption in human GI tract using diltiazem as model drug. *AAPS PharmSciTech* **15**, 1490–1497 (2014).

62. M. Hashim, R. Korupolu, B. Syed, K. Horlen, S. Beraki, P. Karamchedu, A. K. Dhalla, R. Ruffy, M. Imran, Jejunal wall delivery of insulin via an ingestible capsule in anesthetized swine—A pharmacokinetic and pharmacodynamic study. *Pharmacol. Res. Perspect.* **7**, e00522 (2019).

63. J. R. Jørgensen, L. H. E. Thamdrup, K. Kamguyan, L. H. Nielsen, H. M. Nielsen, A. Boisen, T. Rades, A. Müllertz, Design of a self-unfolding delivery concept for oral administration of macromolecules. *J. Control. Release* **329**, 948–954 (2021).

64. L. Cai, G. Chen, L. Sun, S. Miao, L. Shang, Y. Zhao, L. Sun, Rocket-inspired effervescent motors for oral macromolecule delivery. *Adv. Mater.* **35**, e2210679 (2023).

65. G. W. Liu, M. J. Pickett, J. L. P. Kuosmanen, K. Ishida, W. A. M. Madani, G. N. White, J. Jenkins, S. Park, V. R. Feig, M. Jimenez, C. Karavasili, N. B. Lal, M. Murphy, A. Lopes, J. Morimoto, N. Fitzgerald, J. H. Cheah, C. K. Soule, N. Fabian, A. Hayward, R. Langer, G.

Traverso, Drinkable in situ-forming tough hydrogels for gastrointestinal therapeutics. *Nat. Mater.* **23**, 1292–1299 (2024).

66. F. Zhang, Z. Li, Y. Duan, A. Abbas, R. Mundaca-Uribe, L. Yin, H. Luan, W. Gao, R. H. Fang, L. Zhang, J. Wang, Gastrointestinal tract drug delivery using algae motors embedded in a degradable capsule. *Sci. Robot.* **7**, eabo4160 (2022).

67. A. K. Dhalla, Z. Al-Shamsie, S. Beraki, A. Dasari, L. C. Fung, L. Fusaro, A. Garapaty, B. Gutierrez, D. Gratta, M. Hashim, K. Horlen, P. Karamchedu, R. Korupolu, E. Liang, C. Ong, Z. Owyang, V. Salgotra, S. Sharma, B. Syed, M. Syed, A. T. Vo, R. Abdul-Wahab, A. Wasi, A. Yamaguchi, S. Yen, M. Imran, A robotic pill for oral delivery of biotherapeutics: Safety, tolerability, and performance in healthy subjects. *Drug Deliv. Transl. Res.* **12**, 294–305 (2022).

68. S. P. Timoshenko, J. M. Gere, W. Prager, Theory of elastic stability, second edition. *J. Appl. Mech.* **29**, 220–221 (1962).

69. R. P. Feynman, R. B. Leighton, M. Sands, *The Feynman Lectures on Physics* (Addison-Wesley, 2018), vol. 1.

70. X. Liang, G. Chen, I. M. Lei, P. Zhang, Z. Wang, X. Chen, M. Lu, J. Zhang, Z. Wang, T. Sun, Y. Lan, J. Liu, Impact-resistant hydrogels by harnessing 2D hierarchical structures. *Adv. Mater.* **35**, e2207587 (2023).

71. R. W. Ogden, Large deformation isotropic elasticity: On the correlation of theory and experiment for compressible rubberlike solids. *Proc. R. Soc. A.* **328**, 567–583 (1972).

72. R. V. Mises, Mechanik der festen Körper im plastisch- deformablen Zustand. *Math. Phys. Klasse* **1913**, 582–592 (1913).

73. J. Crank, *The Mathematics of Diffusion* (Oxford Univ. Press, ed. 2, 1975).

74. A. J. Bailer, Testing for the equality of area under the curves when using destructive measurement techniques. *J. Pharmacokinet. Biopharm.* **16**, 303–309 (1988).

75. M. Gibaldi, D. Perrier, *Pharmacokinetics* (Marcel Dekker, ed. 2, 1982).

76. M. Vert, J. Mauduit, S. Li, Biodegradation of PLA/GA polymers: Increasing complexity. *Biomaterials* **15**, 1209–1213 (1994).

77..g mundarson, M. J. Herrgård, J. Forster, M. Z. Hauschild, P. Fantke, Addressing environmental sustainability of biochemicals. *Nat. Sustain.* **3**, 167–174 (2020).

78. International Diabetes Federation, *IDF Diabetes Atlas, 10th ed*. (International Diabetes Federation, 2021); https://diabetesatlas.org.

79. T1D Index, Dashboard for Type 1 Diabetes Global Data (2024); https://t1dindex.shinyapps.io/dashboard/.

80. F. M. Bass, A new product growth for model consumer durables. *Manage. Sci.* **15**, 215–227 (1969).

81. H. Ritchie, P. Rosado, M. Roser, "Emissions-weighted carbon price in emissions trading systems," in *CO_2 and Greenhouse Gas Emissions* (Resources for the Future, 2023); https://ourworldindata.org/grapher/weighted-carbon-price-ets.

82. D. Durgalakshmi, S. Balakumar, FTIR and Raman studies of PLA blends and composites. *Spectrochim. Acta A Mol. Biomol. Spectrosc.* **137**, 217–223 (2015).

83. M. Rinaudo, Chitin and chitosan: Properties and applications. *Prog. Polym. Sci.* **31**, 603–632 (2006).

84. J. H. Muyonga, C. G. B. Cole, K. G. Duodu, Fourier transform infrared (FTIR) spectroscopic study of acid soluble collagen and gelatin from skins and bones of young and adult Nile perch (*Lates niloticus*). *Food Chem.* **86**, 325–332 (2004).
